# Mechanical forces impair antigen discrimination by reducing differences in T‐cell receptor/peptide–MHC off‐rates

**DOI:** 10.15252/embj.2022111841

**Published:** 2022-12-09

**Authors:** Johannes Pettmann, Lama Awada, Bartosz Różycki, Anna Huhn, Sara Faour, Mikhail Kutuzov, Laurent Limozin, Thomas R Weikl, P Anton van der Merwe, Philippe Robert, Omer Dushek

**Affiliations:** ^1^ Sir William Dunn School of Pathology University of Oxford Oxford UK; ^2^ Laboratoire Adhesion et Inflammation Aix Marseille University UM 61, INSERM UMRS 1067, CNRS UMR 7333 Marseille France; ^3^ Institute of Physics Polish Academy of Sciences Warsaw Poland; ^4^ Max Planck Institute of Colloids and Interfaces Potsdam Germany; ^5^ Assistance Publique‐Hôpitaux de Marseille Marseille France

**Keywords:** antigen discrimination, molecular forces, T‐cell receptor, Immunology

## Abstract

T cells use their T‐cell receptors (TCRs) to discriminate between lower‐affinity self and higher‐affinity foreign peptide major‐histocompatibility‐complexes (pMHCs) based on the TCR/pMHC off‐rate. It is now appreciated that T cells generate mechanical forces during this process but how force impacts the TCR/pMHC off‐rate remains debated. Here, we measured the effect of mechanical force on the off‐rate of multiple TCR/pMHC interactions. Unexpectedly, we found that lower‐affinity TCR/pMHCs with faster solution off‐rates were more resistant to mechanical force (weak slip or catch bonds) than higher‐affinity interactions (strong slip bonds). This was confirmed by molecular dynamics simulations. Consistent with these findings, we show that the best‐characterized catch bond, involving the OT‐I TCR, has a low affinity and an exceptionally fast solution off‐rate. Our findings imply that reducing forces on the TCR/pMHC interaction improves antigen discrimination, and we suggest a role for the adhesion receptors CD2 and LFA‐1 in force‐shielding the TCR/pMHC interaction.

## Graphical abstract

Low‐affinity TCR‐pMHC interactions are less susceptible to disruption by mechanical force as compared to high‐affinity ones implicating a role for cell adhesion molecules in shielding TCR‐pMHC from applied force to promote antigen discrimination.

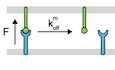

## Introduction

**Figure 1:**
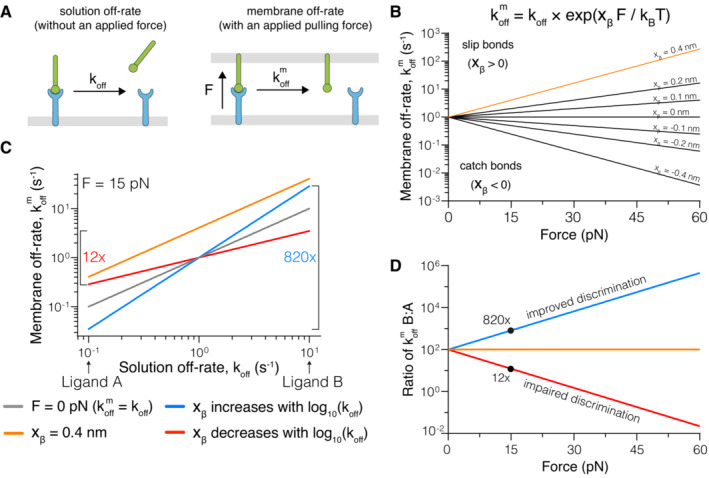
Modelling predicts that force can improve or impair antigen discrimination Depiction of (left) the dissociation of a soluble ligand and its solution off‐rate (k_off_) and (right) a membrane‐anchored ligand and its membrane off‐rate (k^m^_off_), where the interaction is exposed to a pulling force (F).The dependence of k^m^_off_ on the pulling force at the indicated force sensitivity parameters (x_β_) with k_off_ = 1 s^−1^.Dependence of k^m^_off_ on k_off_ at zero force (grey) or under an applied force of 15 pN (coloured lines), when x_β_ is constant (orange), positively (blue), or negatively (red) correlated with the solution k_off_. In this example a 100‐fold change in k_off_ (Ligand A vs. Ligand B) can be increased to a ~ 820‐fold or decreased to a ~ 12‐fold change in k^m^_off_, depending on whether x_β_ is positively or negatively correlated with the k_off_, respectively.The fold‐change in k^m^_off_ for Ligand B versus Ligand A over the applied force. Data information: All calculations are performed using the formula in (B) with x_β_ = 0.4 nm (orange), x_β_ = +0.3 log_10_(k_off_) (blue), and x_β_ = −0.3 log_10_(k_off_) (red) in panels C and D.

T cells use their T‐cell antigen receptors (TCRs) to recognize peptide antigens bound to major‐histocompatibility‐complexes (pMHCs), on the surface of antigen‐presenting‐cells (APCs). It is now well established that T cells discriminate between self and foreign pathogen (or cancer)‐derived pMHC based on the kinetic off‐rate of the interaction (Aleksic *et al*, 2010; Govern *et al*, 2010; Dushek *et al*, 2011; Pettmann *et al*, 2021). Consistent with this mechanism, the off‐rate (k_off_) measured in solution with purified TCR and pMHC can usually predict the T‐cell response. Recently, a number of studies have shown that T cells can generate forces of up to 150 pN as they probe surfaces for pMHC (Husson *et al*, 2011; Feng *et al*, 2017; Colin‐York *et al*, 2019; Ma *et al*, 2022) and can impose forces directly on TCR/pMHC interactions (Göhring *et al*, 2021; Ma *et al*, 2022). This observation is important because T cells discriminate antigens based on the off‐rate in the membrane, termed the membrane off‐rate (k^m^_off_, Fig 1A), and this can be affected by force (Zhu *et al*, 2019). Precisely how molecular forces on the TCR/pMHC interaction impact k^m^_off_, and antigen discrimination is therefore critically important.

It is widely believed that molecular force improves discrimination between low‐affinity self and high‐affinity foreign antigens (Zhu *et al*, 2019). This is based on experimental evidence that force increases k^m^_off_ of low‐affinity pMHC interactions (termed slip‐bonds) while decreasing k^m^_off_ of higher‐affinity pMHC interaction (catch bonds), thus magnifying differences in k_off_. Evidence for catch bonds has been obtained mainly using the biomembrane force probe (BFP) (Liu *et al*, 2014; Sibener *et al*, 2018; Wu *et al*, 2019; Zhao *et al*, 2022), which applies external forces to TCRs on the surface of T cells. It is notable that the magnitude of these catch bonds is appreciably reduced (Liu *et al*, 2015) or abolished (Limozin *et al*, 2019) when applying force to purified forms of the same TCRs. It follows that the catch bond behaviour observed using BFP may be secondary to TCR signalling rather than intrinsic to the TCR/pMHC interaction. While the correlation between the BFP‐measured catch bond behaviour and TCR signalling (Liu *et al*, 2014; Sibener *et al*, 2018; Wu *et al*, 2019; Zhu *et al*, 2019; Zhao *et al*, 2022) is consistent with catch bonds being required for TCR signalling, these data are also consistent with causality being reversed. Further studies on a wider range of TCR/pMHC interactions, and in the absence of TCR signalling, are required to fully elucidate the relationship between force, kinetics, and T‐cell activation.

Several mechanisms are likely to influence the forces experienced by TCR/pMHC interactions. Firstly, in order for TCRs to engage pMHC the T cell and APC membranes need to be brought to within 14 nm (van der Merwe & Dushek, 2011), which requires compression of the large molecules that form the glycocalyx, such as CD45 and CD43, which span 21–45 nm (Chang *et al*, 2016; Siller‐Farfán & Dushek, 2018). By resisting compression, these glycocalyx molecules would generate forces on the TCR/pMHC interaction that are predicted to be 20 pN (Allard *et al*, 2012). Secondly, the dynamics of actin‐based microvilli‐like protrusions that form close contacts between T cells and APC membranes may directly or indirectly pull on TCRs with forces as high as 150 pN (Sage *et al*, 2012; Cai *et al*, 2017; Colin‐York *et al*, 2019). Finally, adhesion receptor/ligand interactions are likely to modulate the forces experienced by TCR/pMHC interactions (Huse, 2017). For example, the adhesion receptors CD2 and LFA‐1 can improve antigen sensitivity (Siller‐Farfán & Dushek, 2018) and antigen discrimination (Pettmann *et al*, 2021), and it is plausible that they do so, at least in part, by influencing the forces experienced by TCR/pMHC interactions.

Here, using a cell‐free system which eliminates TCR signalling, we found that force increased the off‐rate of most TCR/pMHC interactions. Unexpectedly, lower‐affinity interactions were least sensitive to force and more likely to form catch bonds than higher‐affinity interactions, which showed the highest sensitivity to force and this was also observed with molecular dynamics simulations. We show that the best‐characterized catch bond, involving the OT‐I TCR, has an unusually low affinity and the fastest k_off_ yet reported for an agonist TCR/pMHC interaction. Our results imply that force will reduce differences between TCR/pMHC k^m^_off_ and therefore impair antigen discrimination. By incorporating force into the kinetic proofreading model, we show that force‐shielding can account for the ability of adhesion receptors to enhance antigen discrimination. Our study clarifies the role of force in T‐cell antigen recognition and reconciles apparently contradictory reports.

## Results

### Theoretical modelling predicts that mechanical forces can improve or impair antigen discrimination

To investigate how forces affect antigen discrimination, we used Bell's well‐established phenomenological model (Bell, 1978). It provides a simple relationship between the applied force and k^m^_off_, which depends on a receptor/ligand force sensitivity parameter (x_β_). When x_β_ > 0, force increases k^m^_off_, forming a slip‐bond and when x_β_ < 0, force decreases k^m^_off_ forming a catch bond (Fig 1B). In short, the sensitivity of the bond to force increases as the value of its force sensitivity parameter increases.

If all pMHCs that bind the same TCR had the same force sensitivity (i.e. x_β_ is constant), then an applied force will increase the k_off_ of all pMHCs by the same factor (Fig 1C, orange). Because of this, the ratio of the k^m^_off_ between any two pMHC ligands will be unaffected by force (Fig 1D). Given that antigen discrimination is dependent on the fold change in k^m^_off_ (van der Merwe & Dushek, 2011; Zhu *et al*, 2019), it follows that, if force sensitivity is constant, an applied force would not impact antigen discrimination.

We next explored how force would affect antigen discrimination if the force sensitivity is not constant but varies with k_off_ (or affinity, if k_on_ remains constant). It has been proposed that higher‐affinity TCR ligands form catch bonds whereas lower‐affinity ligands form slip‐bonds (Liu *et al*, 2014; Das *et al*, 2015), implying that the force sensitivity increases as the k_off_ increases. We confirmed this using our model, which shows that applied force amplifies the fold change in k_off_ to produce larger fold changes in k^m^_off_ (Fig 1C and D blue). Conversely, if the force sensitivity decreases as the k_off_ increases then applied force dampens differences in k_off_ producing smaller fold changes in k^m^_off_ (Fig 1C and D red). This is illustrated with two test ligands that differ in k_off_ by 100‐fold showing that the fold‐change in k^m^_off_ is > 100‐fold when x_β_ increases with k_off_ (i.e. improved discrimination) but < 100‐fold when x_β_ decreases with k_off_ (i.e. impaired discrimination) under an applied force (Fig 1D).

### The force sensitivity of TCR/pMHC interactions decreases with off‐rate or dissociation constant

**Figure EV1:**
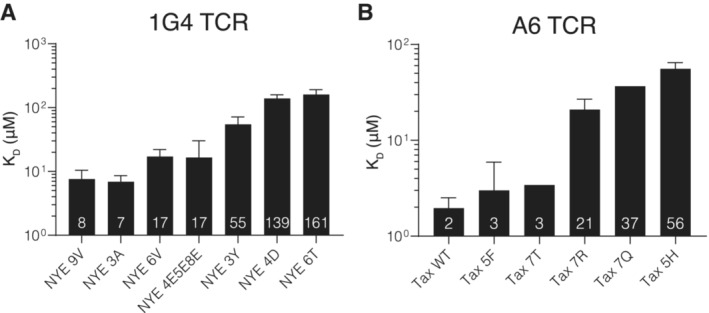
Dissociation constants of TCR/pMHC bond measured by SPR SPR dissociation constants for the 1G4 TCR (*N* = 2–10).SPR dissociation constants for the A6 TCR (*N* = 1–6). Data information: Shown are geometric means with geo. SDs from the *N* independent experiments conducted on different days with the numbers in the bar indicating the geometric mean. Data is partially reproduced from Fig 1G and H of our previous measurements (Pettmann *et al*, 2021) with additional repeats and pMHC variants. Source data are available online for this figure.

**Figure 2:**
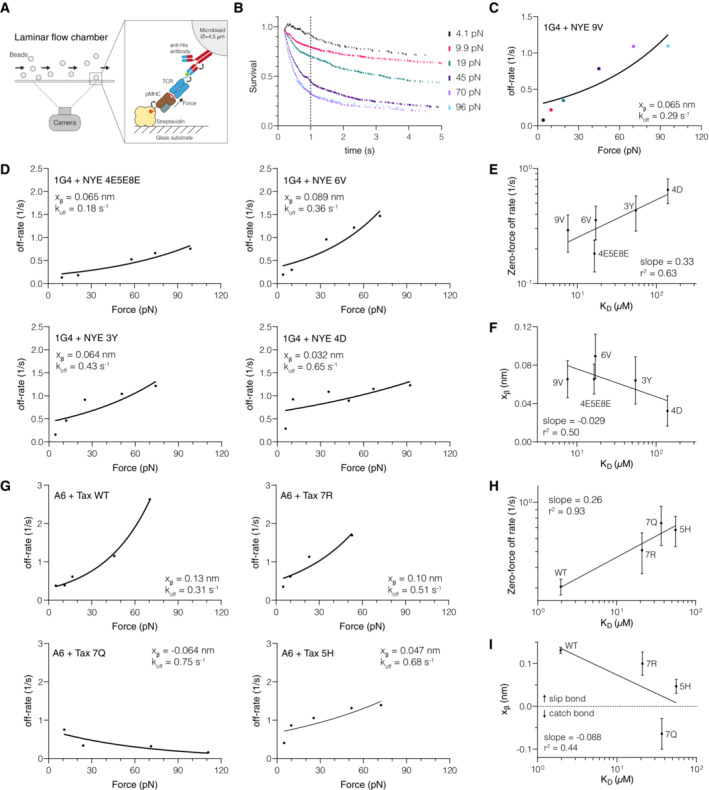
Direct measurements of force sensitivity (x_β_) for the 1G4 and A6 TCRs reveal a negative correlation with K_D_ (k_off_/k_on_) Schematic of the laminar flow chamber assay. All experiments were performed at physiological temperatures (37°C) and forces (< 120 pN). The biotinylated pMHC is anchored to the streptavidin‐coated surface by a flexible linker and the TCR contains a flexible linker with a his‐tag that allows for coupling to the bead (see Fig EV2 for details).Example of bead survival distributions for the 1G4 TCR binding NYE 9V. The survival probability at 1 s is shown as a dotted vertical line and is used to calculate the off‐rate under force: −ln (survival at 1 s)/1 s.Off‐rates under force obtained from (B) are fitted with Bell's model (solid line).Off‐rates under force for additional pMHCs interacting with the 1G4 TCR fitted to Bell's model (solid line).Correlation of the dissociation constant (KD) measured previously by SPR and the extrapolated zero‐force flow chamber off‐rate (k^0^_off_) from fitting Bell's model for the 1G4 TCR.Correlation of the SPR dissociation constant (K_D_) with the fitted force sensitivity parameter (x_β_) for the 1G4 TCR.Off‐rates under force for pMHCs interacting with the A6 TCR fitted to Bell's model (solid line).Correlation of the dissociation constant (K_D_) measured previously by SPR and the extrapolated zero‐force flow chamber off‐rate (k^0^_off_) from fitting Bell's model for the A6 TCR.Correlation of the SPR dissociation constant (K_D_) with the fitted force sensitivity parameter (x_β_) for the A6 TCR. Data information: Error bars (SE) in E, F, H, I are obtained from the fit. The number of independent experiments performed on different days that were combined to produce the estimated off‐rates is 7 (1G4/9V), 8 (1G4/6V), 8 (1G4/4E5E8E), 10 (1G4/3Y), 6 (1G4/4D), 9 (A6/Tax WT), 11 (A6/5F), 9 (A6/7Q), 8 (A6/7R), 8 (A6/5H). Source data are available online for this figure.

**Figure EV2:**
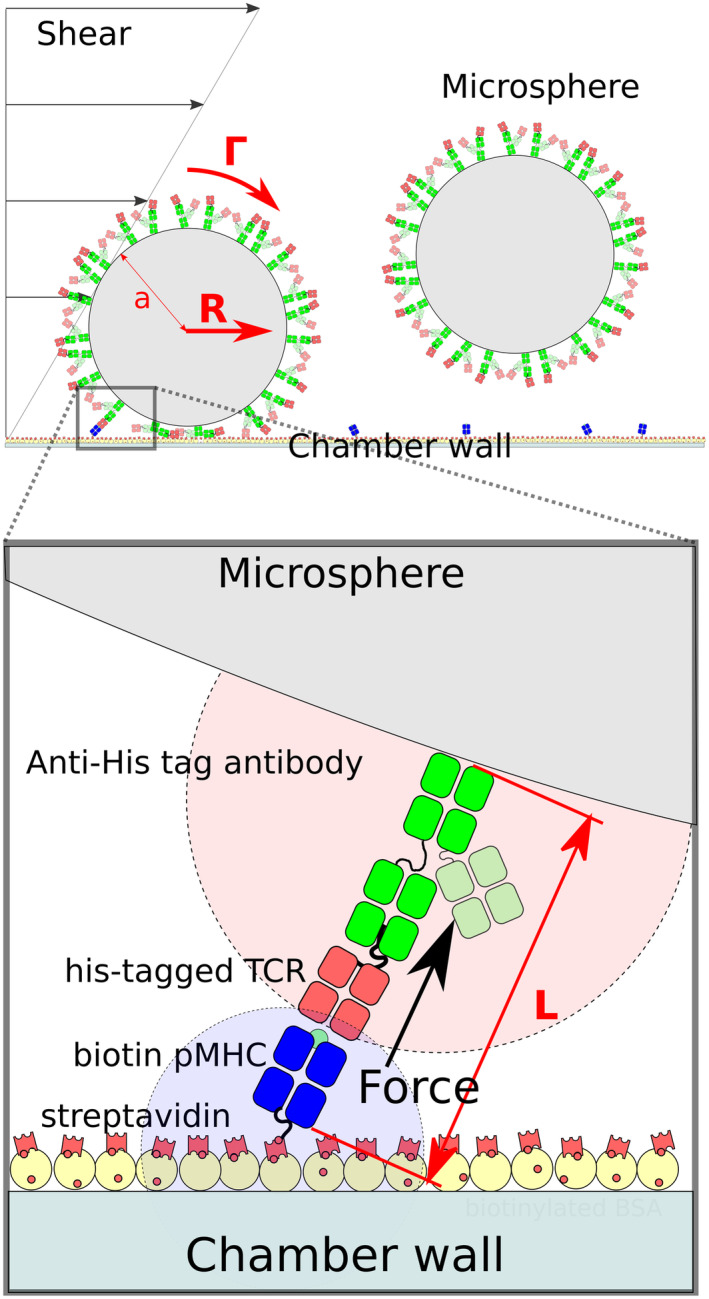
Flow velocity resolves into a normal force on the TCR/pMHC as a result of anchoring flexibility Biotinylated pMHC and antibody‐bound TCR have rotational freedom as a result of a flexible linker between the avi‐tag and the pMHC and between the TCR and the his‐tag that the antibody binds. This allows their binding site to diffuse in volumes represented by potions of disc in light blue for the pMHC and light red for TCR. Near to the surface of the chamber, the flow velocity is given by a first‐order approximation: v(z) = Gz, where z is the distance to the surface in nm and G is the shear rate constants in s^−1^. The shear stress T_s_ (in N/m^2^ or pascals) applied corresponds to the force applied by the flow per surface unit and is calculated by the product between G and the viscosity of the medium (μ) in Pa/s: T_s_ = μG. Taking into account the dimensions of the LFC section lxH, shear rate can be determined for a given flow Q using the formula: G ≈ 6Q/H^2^. When the microsphere makes a link with the surface the flow creates a hydrodynamic force (R) given by the equation: R ≈ 32μa^2^G =1.7005 × 6πμa^2^G where a is the radius of the microsphere. The microsphere is also subjected to a torque force (Γ): Γ ≈ 0.9440 × 4μa^3^G. In addition, a lever effect increases the force applied to the interaction, so the total force applied (F) is given by the equation: F ≈ (R + Γ/a)√(a/2L) where L is the length of the formed bond, that is, the length of the link. Additional details can be found in (Pierres *et al*, 1995).

Given that the impact of force on antigen discrimination critically depends on the relationship between k_off_ and x_β_, we set out to directly measure it. We selected the 1G4 and A6 TCRs for this study because we recently obtained accurate solution affinities for these TCRs binding a panel of peptide ligands presented on HLA‐A*02:01 (Pettmann *et al*, 2021) (Fig EV1) and used our laminar flow chamber (LFC) apparatus to examine how force impacts bond duration (Robert *et al*, 2012; Limozin *et al*, 2019). In this assay, beads coated with TCR flow over a low density of pMHC while a camera records bead motion (Fig 2A, Movie EV1). As a result of the anchoring flexibility of the TCR on the bead and the pMHC on the surface, the flow velocity resolves into a pulling force along the TCR/pMHC bond axis (Fig EV2) (Pierres *et al*, 1995; Robert *et al*, 2012). The duration of TCR/pMHC binding is determined by an automated image analysis algorithm that detects the duration of bead arrests. This automated workflow allowed us to perform a large number of experiments (*N* = 113) using different flow velocities and different pMHC surface densities measuring a total of 13 different TCR/pMHC interactions.

**Figure EV3:**
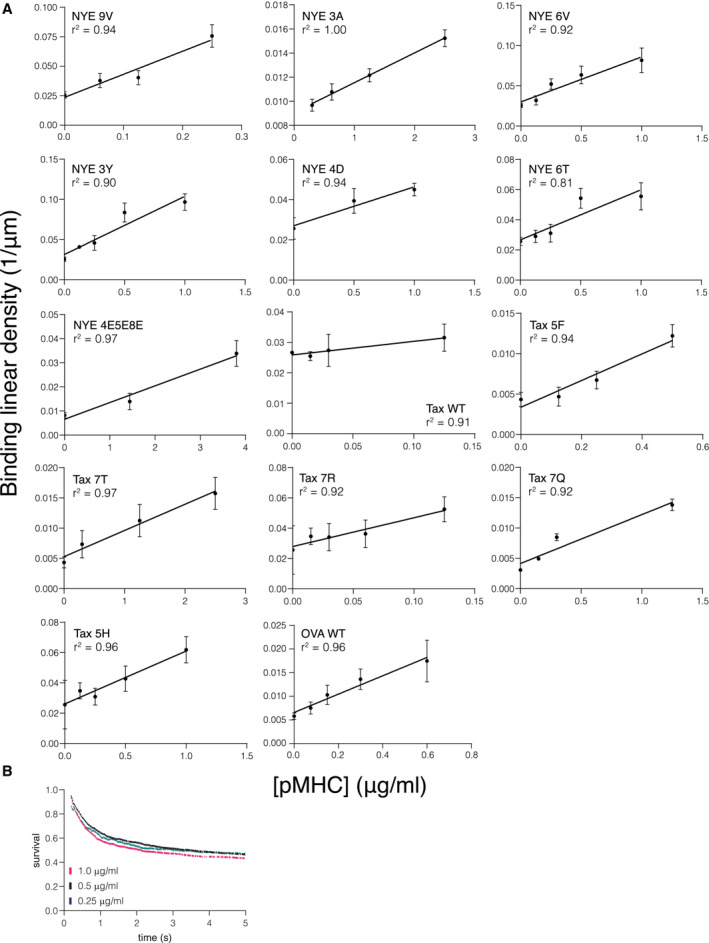
Linear binding density correlation indicates assay operation in single molecule bond regime Linear binding densities for all tested TCR/pMHC combinations. A linear correlation indicates a single‐molecule binding regime.Example of survival curves of the same TCR/pMHC interaction with different pMHC immobilization levels. Shown is 1G4/NYE 6T. Data information: Data points in (A) represent mean and SD from independent experiments using 1G4 TCR binding NYE 9V (*N* = 7), NYE 3A (*N* = 9), NYE 6V (*N* = 8), NYE 3Y (*N* = 10), NYE 4D (*N* = 6), NYE 6T (*N* = 9), NYE 4E5E8E (*N* = 8) and A6 TCR binding Tax WT (*N* = 9), Tax 5F (*N* = 11), Tax 7T (*N* = 11), Tax 7R (*N* = 8), Tax 7Q (*N* = 9), Tax 5H (*N* = 8), and the OT‐I TCR binding OVA (*N* = 11). Source data are available online for this figure.

**Figure EV4:**
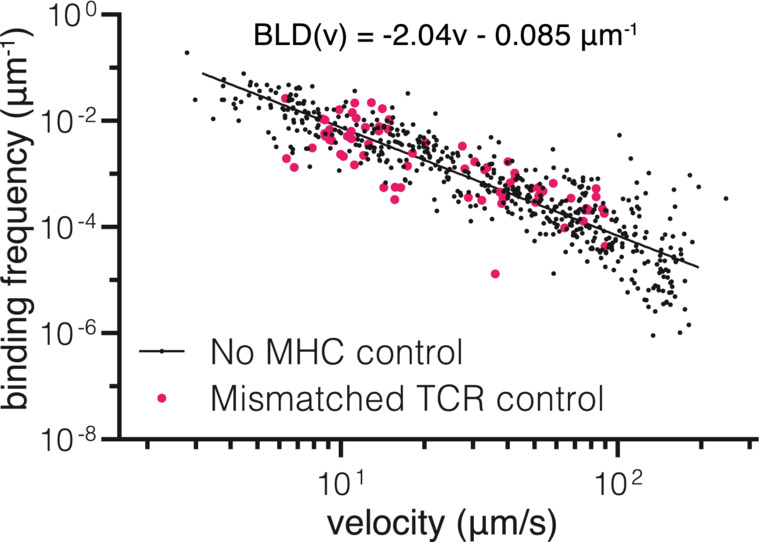
Binding linear density for control surfaces over flow velocity Binding linear density (BLD) of TCR‐coated beads over surfaces without pMHC (black – no MHC control) or surfaces with a pMHC that does not match the TCR on the bead (pink – mismatch). Each dot represents one experiment/flow cell. Mismatched control is pooled data from experiments with 1G4/OVA, 1G4/Tax WT, 1G4/Tax 7R, A6/NYE 4D, A6/NYE 6V, and A6/OVA. No pMHC control was fitted with a linear curve in loglog space.Source data are available online for this figure.

**Figure EV5:**
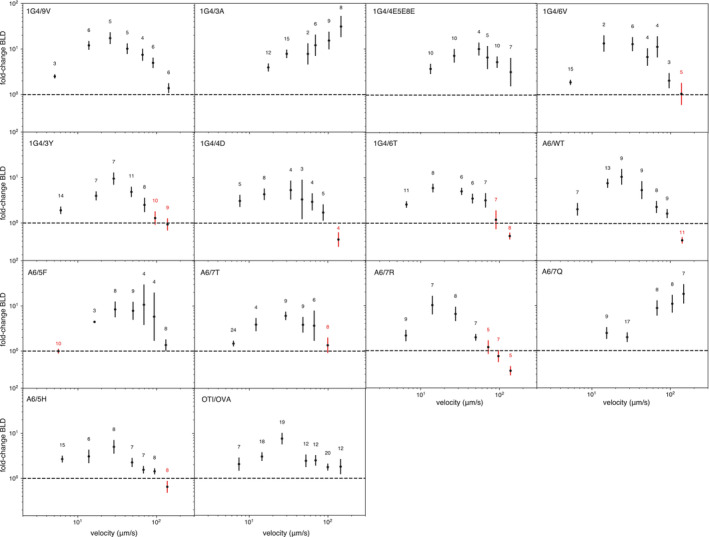
Specific TCR/pMHC interactions show higher binding linear densities at most flow velocities The ratio of BLD between the indicated samples and the no pMHC control. Points mark the geometric mean with geo. SEM at different velocity bins. Velocity categories where the lower error bar < 1 were excluded (red). The number of experiments binned in each velocity category is indicated above each data point.Source data are available online for this figure.

We first confirmed that bead arrests were mediated by single bonds. In this regime, the density of arrested beads or the binding linear density (BLD) is expected to linearly increase with the density of pMHC on the surface and the survival distribution is expected to be independent of pMHC density (Pierres *et al*, 1995; Robert *et al*, 2012; Limozin *et al*, 2019) and we confirmed this for our data (Fig EV3). Moreover, the fitted zero‐force off‐rate from the LFC was similar to measurements using surface plasmon resonance (see next paragraph). We next confirmed that the BLD was similar between surfaces without pMHC or those with an irrelevant pMHC (Fig EV4) and that surfaces with a specific pMHC exhibited a larger BLD across different flow velocities (Fig EV5). As expected, at fast flow velocities we observe a more similar BLD because the short encounter duration between specific TCR and pMHC prevented binding and hence bead arrest (Limozin *et al*, 2019) and in the limit of very slow flow velocities it is not possible to distinguish bead arrest from the slow bead motion. These constraints limit the LFC to a range of flow velocities that translate into pulling forces between ~ 5–120 pN with the upper bound determined, in part, by the binding rate and strength of the specific TCR/pMHC interaction. Finally, survival distributions were generated using the duration of individual beads for each TCR/pMHC pair and corrected by the corresponding distribution of arrests without pMHC for each paired velocity. We focus on the first 5 s of the distribution because on longer timescales measurement of bead arrest is often interrupted by a collision from a second bead.

We found that increasing force decreased survival for 1G4 binding the agonist 9V peptide from the NY‐ESO‐1 cancer testis antigen, which is indicative of a slip bond (Fig 2B). We calculated off‐rates based on the survival fraction at 1 s as in our previous work (Robert *et al*, 2012) and fit the data using Bell's model to determine the force sensitive and the zero‐force off‐rate (Fig 2C). The zero‐force off‐rate (k^0^_off_ = 0.29 s^−1^) agreed with the published solution k_off_ obtained using SPR (0.33 s^−1^) (Aleksic *et al*, 2010). We found that four additional ligands also formed slip bonds (Fig 2D), and as expected, the extrapolated zero‐force off‐rate correlated with the K_D_ values measured by SPR (Fig 2E). We used K_D_ values because they correlate with the solution off‐rates and are easier to measure accurately, especially for weakly binding pMHCs.

We noted that the force sensitivity x_β_ varied between the pMHCs (Fig 2C and D) and, surprisingly, displayed a negative correlation with their K_D_ (Fig 2F). This means that an applied force disproportionately accelerates the off‐rate of higher‐affinity interactions. This can be observed by comparing the slopes on the off‐rate curves (Fig 2D) where the higher‐affinity 6V pMHC (top right panel) shows a much larger increase in off‐rate with applied force than the lower affinity 4D pMHC (bottom right panel).

We next investigated whether the negative correlation could be observed for a different TCR. We found that the A6 TCR formed a slip bond with its wild‐type Tax peptide from Human T‐lymphotropic virus (HTLV), slip‐bonds with three additional ligands (5F, 7R, 5H), and one catch bond (7Q) (Fig 2G). The estimated zero‐force off‐rate correlated with K_D_ measured by SPR (Fig 2H). As seen with the 1G4 TCR, we observed a negative correlation between the force sensitivity x_β_ and the K_D_ (Fig 2I).

The above analysis relied on estimating off‐rates based on the survival fraction at 1 s as in our previous work (Robert *et al*, 2012). This method was used in part because the survival distributions did not follow a single exponential decay and this has been termed “history dependence of bond dissociation,” which may suggest multiple bond states (Robert *et al*, 2012). To confirm the general validity of our results, we repeated the analysis by calculating the off‐rate based on the survival fraction at 2 s or by directly fitting an exponential function that included a baseline plateau parameter to the survival distribution (Appendix Figs S1 and S2). We fit Bell's model to estimate the zero‐force off‐rate and the force sensitivity x_β_ (Appendix Figs S3 and S4) and found, as before, that they exhibited a positive and negative correlation, respectively, with K_D_ measured by SPR (Appendix Figs S5 and S6). Lastly, we also performed the analysis on the raw data prior to correcting for non‐specific binding and again, found the same results (Appendix Figs S7–S12). Together, this suggests that our conclusions are robust to our data analysis methods.

We found three interactions (the 1G4 TCR binding 3A and 6T, and the A6 TCR binding 7T) that did not display canonical slip or catch bonds and could not be accurately fit with Bell's model (Appendix Fig S13). These interactions displayed the expected binding linear densities (Appendix Fig S3). Importantly, these bonds were slip bonds at forces thought to be more physiologically relevant (< 20 pN) (Göhring *et al*, 2021). Interestingly, 3A and 6T did display canonical slip and catch bonds, respectively, at 25°C (Robert *et al*, 2012), suggesting that some TCR/pMHC interactions can have a more complex unbinding pathway at 37°C.

We previously measured force sensitivity for the 1G4 TCR at 25°C using larger forces (> 30 pN) (Robert *et al*, 2012). In that study, which used a different definition of the force sensitivity parameter (F_0_ = *k*_B_T/ x_β_), it was reported that no significant (linear) correlation was observed between k_off_ and F_0_. However, when we plot the force sensitivity using the definition in the present study (x_β_) against the log of k_off_, we observed a striking negative correlation (Appendix Fig S14A). Therefore, our previous results are consistent with the present study.

We identified only one other study that measured force sensitivity and affinity. In that study, x_β_ was measured for a panel of nine antibodies binding fluorescein using atomic force microscopy (Schwesinger *et al*, 2000). Interestingly, a striking negative correlation is also observed between force sensitivity and off‐rate, which we reproduce by directly plotting x_β_ over the log of k_off_ (Appendix Fig S14B). This suggests that a negative correlation between force sensitivity and off‐rate (or K_D_) may be a general feature of antigen receptor interactions.

### Molecular dynamic simulation predicts a negative correlation between force sensitivity and off‐rate

**Figure 3:**
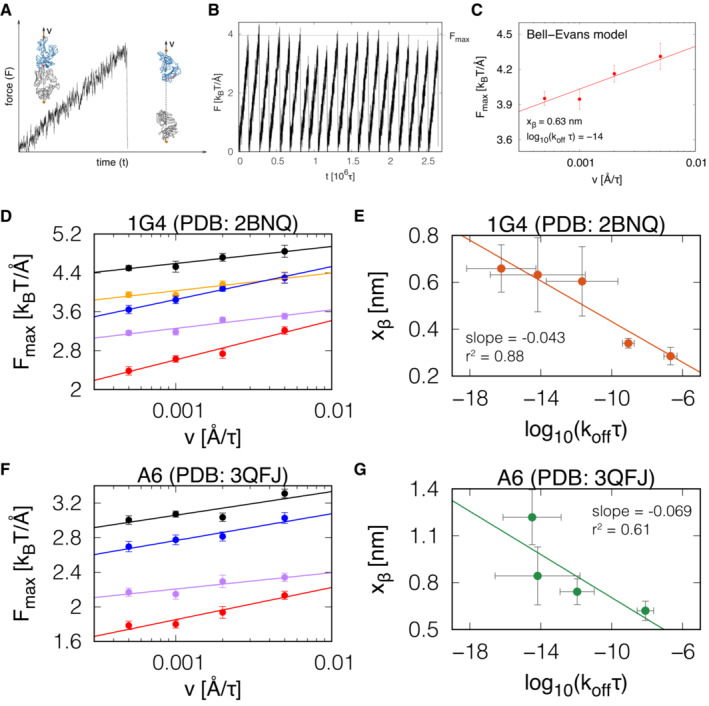
Structure‐based coarse‐grained molecular dynamics simulations support a negative correlation between x_β_ and k_off_ The response force (F) on the TCR/pMHC interaction when  the C‐terminus of the MHC  is pulled with constant velocity (ν) and the C‐terminus of the TCRβ is fixed in space. Insets: the TCR is shown in grey, the MHC in blue, and the peptide in red. The sites where the springs are attached are marked in orange. The direction of pulling is marked by a dashed line.A plot of F(t) from *N* = 20 independent simulation trajectories with the same value of the pulling speed (ν). The grey horizontal line indicates the average force (F_max_) at which the TCR‐pMHC complex dissociates.The dependence of F_max_ (*N* = 20) on ν from the simulations follows the Bell‐Evans model (solid line), which produces estimates of k_off_ and x_β_.The dependence of F_max_ on ν obtained from simulations of five complexes for the 1G4 TCR‐pMHC complex (PDB: 2BNQ): (i) with all native contacts between the peptide and the TCR (black), (ii) without the native contacts between the peptide residues 4 to 8 and the TCR (orange), (iii) only with the native contacts between the peptide and the TCRα (blue), (iv) only with the native contacts between the peptide and the TCRβ (purple), and (v) with no native contacts between the peptide and the TCR (red) included in the coarse‐grained model. Solid lines are the fit of the Bell‐Evans model.The values of k_off_ and x_β_ from the fit of the Bell‐Evans model to the simulation data shown in panel D. The solid line is a linear fit on log‐transformed x‐axis values.Analogous to panel D but for the A6 TCR‐pMHC complex (PDB: 3QFJ). The colour code is as in panel D.The values of k_off_ and x_β_ from the fit of the Bell‐Evans model to the simulation data shown in panel F. The solid line is a linear fit on log‐transformed x‐axis values. Data information: Error bars in C, D, and F represent SEM of the *N* = 20 independent simulations and error bars in E, G represent SEM from the least‐square fit of the data in D, F, respectively. Source data are available online for this figure.

We next determined whether the negative correlation between k_off_ and x_β_ can also be observed using molecular dynamics simulations. We employed a structure‐based coarse‐grained model (Różycki *et al*, 2014) where the C‐terminus of the TCRβ was fixed in space and the C‐terminus of the MHC was moved with constant speed (ν) to generate a response force (F) on the TCR/pMHC interaction. Unbinding could readily be observed by F dropping from its maximal value to zero (Fig 3A). The maximum force (F_max_) was determined from 20 independent simulations (Fig 3B) and repeated for four different values of ν (Fig 3C). Fitting the Bell‐Evans formula (equation 2 in Schwesinger *et al*, 2000) provides estimates of k_off_τ and x_β_, where τ is the simulation timestep (~ 1 ns) and is constant for all calculations.

To generate TCR/pMHC complexes with different affinities, we performed simulations using the 1G4 TCR/pMHC complex where all native contacts between the peptide and TCR were included or where certain contacts were excluded (Fig 3D). We fitted the Bell‐Evans model and consistent with our experimental data, a plot of x_β_ over k_off_ from these simulations revealed a negative correlation with a dimensionless slope of −0.043 (Fig 3E), which is similar to the experimental slope of −0.029 observed for 1G4 (Fig 2F). We repeated the analysis for A6 TCR/pMHC complexes (Fig 3F) also finding a negative correlation with a dimensionless slope of −0.069 (Fig 3G), which again was similar to the experimental slope of −0.088 (Fig 2I).

Lastly, we repeated these simulations using two additional TCRs. When using the F24 TCR interacting with an HIV Gag‐derived peptide presented on HLA‐DR11 (Galperin *et al*, 2018), we found the same negative correlation with a dimensionless slope of −0.041 (Appendix Fig S15A and B). When using the TK3 TCR interacting with an EBV peptide presented on HLA‐B*35:01 (Gras *et al*, 2010), we found a more modest negative slope of −0.024 (Appendix Fig S15C and D).

Taken together, we performed a total of 400 independent molecular simulations across four different TCRs/pMHCs complexes that support a negative correlation between x_β_ and k_off_ with the quantitative slope varying across different TCR/pMHC complexes. As with our experimental data, this result implies that pulling forces will impair antigen discrimination by reducing fold‐differences in off‐rates because higher‐affinity interactions will be more sensitive to force compared to lower‐affinity interactions.

### The OT‐I TCR binds OVA pMHC with a low‐affinity and an exceptionally fast off‐rate

Previous studies used the biomembrane force probe to report a catch bond for the OT‐I TCR binding its agonist OVA pMHC ligand (Liu *et al*, 2014, 2015). We confirmed this in our laminar flow chamber assay using the survival at 1 s to calculate off‐rates (Appendix Figs S16 and S17A and B) and similar results were obtained when using survival at 2 s or when directly fitting an exponential to the data (Appendix Fig S18). Since the negative correlation between x_β_ and k_off_ predicts that lower‐affinity interactions with fast off‐rates are likely to be resistant to or even benefit from force, we considered whether the OT‐I/OVA interaction may have an unusually low affinity and fast off‐rate. While early studies reported high affinities and slow off‐rates at 37°C for the OT‐I/OVA interaction (k_off_ ~ 0.02 s^−1^) (Rosette *et al*, 2001), more recent measurements reported lower affinities and faster off‐rates, even though they were performed at 25°C (Stepanek *et al*, 2014; Liu *et al*, 2015). This discrepancy prompted us to repeat these measurements using an SPR protocol optimized for low affinities at 37°C (Pettmann *et al*, 2021).

We injected purified OT‐I over a surface with low levels of purified OVA pMHC and obtained an affinity of K_D_ = 34 μM (Appendix Fig S17C–F), which is unusually weak for TCR interactions with an MHC‐I restricted agonist (Cole *et al*, 2007). The dissociation phase produced very fast off‐rates that were at the SPR instrument limit and therefore, we repeated the measurements using a different instrument based on Grating‐Coupled Interferometry (GCI) (Appendix Fig S17F–H). Both GCI and SPR produced comparably fast off‐rates of 4.3 and 5.7 s^−1^, respectively (Appendix Fig S17I). Using the measured off‐rate and affinity, we calculated that the on‐rate for this interaction was 0.13 μM^−1^ s^−1^ (Appendix Fig S17J), which is within the range obtained with other TCRs (Cole *et al*, 2007).

Our analysis shows that the OT‐I/OVA interaction, which forms a catch bond, has an unusually fast off‐rate for an agonist TCR/pMHC interaction (Cole *et al*, 2007), 13‐17‐fold faster than the 1G4/9V interaction (Aleksic *et al*, 2010). This is consistent with our findings that TCR/pMHC interactions with fast off‐rates (lower‐affinity) are more likely to be resistant to forces (weak slip bonds) or benefit from them (catch bonds).

### Enhanced antigen discrimination by adhesion interactions can be explained by “force‐shielding” TCR/pMHC interactions

It is well established that engagement of the T‐cell adhesion receptors CD2 (which binds CD58) and LFA‐1 (which binds ICAM‐1) improve the sensitivity of T cells to antigens (Huse, 2017; Siller‐Farfán & Dushek, 2018; Zhu *et al*, 2019; preprint: Burton *et al*, 2021). Recently, we reported that this improvement in sensitivity was progressively lost as the antigen affinity was lowered and consequently, engagement of these receptors increased the ability of T cells to discriminate antigens (Pettmann *et al*, 2021) but the mechanism remains unclear.

**Figure 4:**
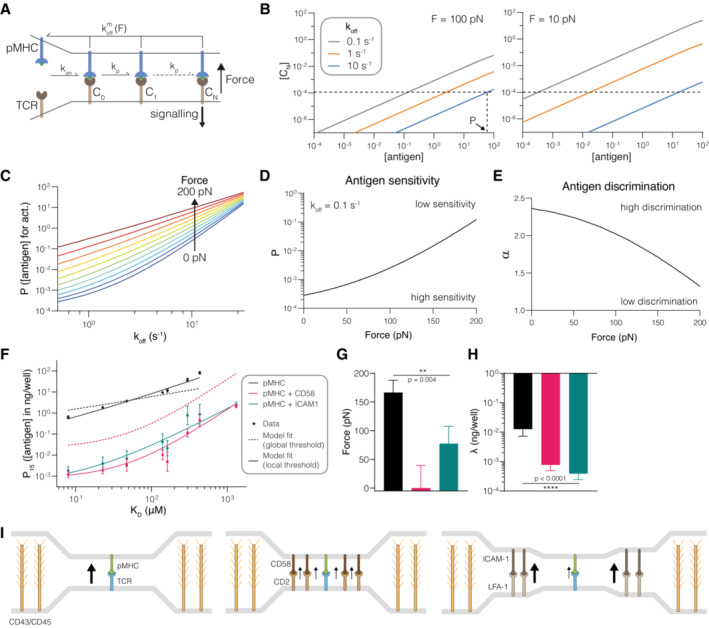
The ability of adhesion receptors to enhance T‐cell antigen discrimination and sensitivity can be explained by them shielding the TCR/pMHC interaction from mechanical force Schematic of the kinetic proofreading model modified to allow molecular forces to impact the membrane off‐rate (k^m^_off_).The concentration of productively signalling TCRs (C_N_) over the antigen concentration for antigens with the indicated zero‐force off‐rates (k_off_). Shown is the effect of forces at 100 pN (left) and 10 pN (right). The horizontal dashed line is the threshold value of C_N_ required for activation in the model (λ). The vertical dashed line shows the antigen concentration (P) required to elicit activation by the antigen with the largest value of k_off_.The antigen concentration required to elicit activation (P) over k_off_ for the indicated molecular forces on the TCR/pMHC interaction.Antigen sensitivity quantified as the value of P from (C) over the applied force for a higher‐affinity antigen with k_off_ = 0.5 s^−1^.Antigen discrimination quantified by the discrimination power (α) over the applied force. Discrimination power is defined as the ratio of the logarithm of the fold‐change in P over the fold‐change in k_off_ for a higher‐affinity ligand (k_off_ = 0.5 s^−1^) and a lower‐affinity ligand (k_off_ = 10^1.5^ s^−1^) from panel (C).Experimental value of P (symbols) over antigen affinity fitted by the kinetic proofreading model with force (dashed and solid lines). The value of P is defined as the concentration of pMHC required for 15% upregulation of CD69 above baseline and is shown as the mean with SEM from 4 independent experiments. All experimental data are taken from fig 4 in (4).The fitted value of the applied force with SEM.The fitted value of λ with SEM.Proposed mechanisms by which adhesion receptors can reduce pulling forces on TCR/pMHC interactions (black arrow). Molecular forces on individual TCR/pMHC interactions can be produced by multiple mechanisms, including a mismatch in size between the short TCR/pMHC interaction (~ 14 nm) and larger surface molecules, such as CD43 and CD45 that extend 21–45 nm (left panel). These forces may be reduced by force‐sharing with the CD2/CD58 adhesion molecules that span the same distance and can therefore closely colocalize (middle panel). Alternatively, the larger LFA‐1/ICAM‐1 adhesion interaction may reduce forces by acting as a diffusional barriers to maintain CD43 and CD45 further away from TCR/pMHC interactions, and tether and stabilize cellular processes such as microvilli, thereby reducing the forces imposed on all interactions within an area of close contact surrounded by LFA‐1/ICAM interactions (right panel) Data information: An F‐test is used to produce a *P*‐value for the null hypothesis that the applied force or λ is the same across the three conditions. All calculations (B–E) and data fitting (F‐H) used *N* = 2.46 and k_p_ = 2.15 s^−1^ (average of CD58 and ICAM‐1 conditions) taken from fig 5 in (4). Source data are available online for this figure.

To address this, we first modified the standard kinetic proofreading model by including the impact of force (Fig 4A). In the standard model, the duration of pMHC binding to the TCR, which critically determines whether binding is translated to a productive TCR signal, is determined by the solution off‐rate. Here, we were able to replace the solution off‐rate with the predicted membrane off‐rate (k^m^_off_) using the force sensitivity relationship that we had measured for the 1G4 TCR (Fig 2F, x_β_ = −0.029log_10_ (K_D_)‐0.11). We found that reducing molecular forces from 100 to 10 pN increased differences in signalling TCRs between three test ligands (Fig 4B). This effect is illustrated when the concentration of ligand (P) required to produce a threshold level of TCR signal is plotted over the zero‐force koff (Fig 4C). It is evident that P increases faster for lower values of k_off_ compared to higher values under an applied force. Consequently, the model predicts that increasing force on the TCR/pMHC interaction decreases antigen sensitivity (Fig 4D) and reduces antigen discrimination (Fig 4E).

To understand how adhesion receptors impact the force on the TCR/pMHC interaction, we next fitted the modified model to data we previously generated (Pettmann *et al*, 2021). Briefly, primary human CD8^+^ T cells expressing the 1G4 TCR were stimulated with eight antigens either alone or in combination with CD58 or ICAM‐1, and the concentration of antigen required to elicit activation (P) was plotted against the solution affinity (Fig 4F). We fixed the number (*N*) and rate (k_p_) of each step to those we previously identified (Pettmann *et al*, 2021) to obtain a mechanical proofreading model with only two fitting parameters: the force applied on the TCR/pMHC interaction (F) and a quantity that is proportional to the threshold concentration of productively signalling TCRs (λ). Intuitively, lower values of λ mean that a lower TCR signal is sufficient to activate a cellular response.

We first asked whether differences in force are sufficient to explain the changes in P for all antigens. To do this we fitted F while constraining λ to a single “global” value (Fig 4F, dashed line). We found that, while the fit qualitatively reproduced the data, in that ligation of CD2 or LFA‐1 increased both antigen sensitivity and discrimination, it failed to quantitatively fit the data. Inspection of the fits suggests that force shielding by CD2 and LFA‐1 (dashed lines) can fully account for their ability to enhance antigen discrimination (changes in slope) but only partially account for their ability to enhance antigen sensitivity (vertical shifts/changes in potency). When we also allowed λ to vary, we observed an excellent fit (Fig 4F, solid line) with reduced TCR/pMHC forces (Fig 4G) and reduced TCR signalling thresholds (Fig 4H) upon ligation of adhesion receptors.

In summary, these results suggest that force‐shielding by CD2 and LFA‐1 can fully account for their ability to improve antigen discrimination and partially account for their ability to enhance sensitivity, suggesting that sensitivity is further enhanced by processes, presumably signalling by CD2 and LFA‐1, that amplify TCR signals.

## Discussion

We have used a modified form of Bell's model to explore how molecular forces might impact antigen discrimination. This revealed that discrimination would be impaired by force if the sensitivity of TCR/pMHC interactions to force (x_β_) increases as k_off_ decreases (i.e. as their affinity increases). Using cell‐free experiments, we showed that higher‐affinity TCR/pMHC interactions are indeed more susceptible to force than lower‐affinity interactions. In other words, we show that x_β_ is negatively correlated with k_off_, such that low‐affinity interactions are more likely to form catch bonds (x_β_ < 0). Consistent with this, we show that the best‐characterized catch bond (OT‐I/OVA) has an unusually low affinity and the fastest k_off_ yet reported for an agonist TCR/pMHC interaction.

Using structure‐based coarse‐grained molecular dynamics simulations of four TCRs, including 1G4 and A6, we confirmed the negative correlation between x_β_ and k_off_, suggesting that this may be a general structural feature of TCR/pMHC interaction. The large number of independent simulations required to generate a single x_β_ vs. k_off_ plot (~ 100) meant that it was not feasible to perform computationally intensive all‐atom simulations. Moreover, they typically allow for only a few hundred nanoseconds of simulation, which is appreciably shorter than typical TCR/pMHC lifetimes under physiologically relevant forces. To obtain unbinding on the nanosecond timescale using all‐atom simulations, other studies have imposed high pulling speeds that can lead to unphysiologically high rupture forces (Sibener *et al*, 2018; Wu *et al*, 2019). For example, Wu *et al* (Wu *et al*, 2019) have used pulling speeds of 0.1 nm/ns, which is 200 to 2000 times larger than those used in the present study. It will be important to reproduce our results using all‐atom simulations when it becomes computationally feasible under physiological pulling speeds.

Our findings using purified TCR and pMHC in a cell‐free system show that high‐potency pMHC ligands that activate T cells usually form slip bonds. In contrast, assays using live T cells have found that all high‐potency pMHC ligands formed catch bonds (Kim *et al*, 2009; Liu *et al*, 2014; Das *et al*, 2015; Wu *et al*, 2019). A plausible explanation for these differences is the additional effect of TCR signalling when using live cells. It has been shown that TCR triggering induces cellular responses that enhance TCR/pMHC binding and prolong half‐life, such as coreceptor recruitment, TCR clustering, and changes in the cytoskeleton or membrane (Varma *et al*, 2006; Dushek & van der Merwe, 2014; Pielak *et al*, 2017). In the biomembrane force probe, initial TCR binding enhances subsequent binding events (Zarnitsyna *et al*, 2007) and CD8 recruitment induced by TCR triggering increases TCR/pMHC binding (Jiang *et al*, 2011). We suggest that engagement of activatory pMHC with TCR induces TCR signalling which in turn induces processes that enhance pMHC binding, generating catch bonds. This is plausible in BFP assays because the TCR/pMHC bond duration is measured multiple times using the same T cell, which allows TCR signalling induced by initial pMHC binding to enhance subsequent binding. Thus, the catch bond observed using live T cells may be a consequence of TCR triggering, accounting for correlation between catch bond behaviour and TCR triggering. In support of this, the magnitude of the catch bond was much lower when the same TCR/pMHC interaction was examined using purified TCR instead of native TCR on live T cells (Liu *et al*, 2015). Furthermore, high‐affinity ligands for the 1G4 TCR displayed slip bonds using purified TCR (present study) and a catch bonds using the BFP with live T cells (Wu *et al*, 2019). In conclusion, these apparently contradictory results can be reconciled if the binding of agonist pMHC to TCR induces active processes that enhance pMHC binding, thereby generating effective catch bonds.

Why might TCR/pMHC interactions with a lower affinity/faster k_off_ be more resistant to force than higher affinity interactions? One intuitive explanation is that the contact interface will be less intimate with low versus high‐affinity interactions. It follows that the notional distance between the energy minimum and the energy barrier along the dissociation pathway would be shorter for lower affinity interactions. Thus, x_β_, which has units of length and can be thought of as equivalent to this distance, will be shorter for low‐ versus high‐affinity interactions. Assuming that the height of the energy barrier decreases less than this distance for lower‐affinity interactions, shortening will make the slope up to the energy barrier steeper, and, since this slope can be considered to be equivalent to force, this implies greater force is needed to reach the barrier. While the unbinding pathway for protein/proteins interactions is likely to be complex and varying, it seems reasonable that mutations within a given TCR/pMHC complex that increase or decrease the affinity would increase or decrease respectively, the intimacy of the contact interface and thus x_β_ explaining our experimentally observed negative correlation. In contrast, given the large structural diversity between TCR/pMHC binding interfaces (Rudolph *et al*, 2006), and the fact that TCRs do not encounter foreign agonist pMHC during their development, it is difficult to envisage a molecular explanation for how high‐affinity interactions all form intrinsic catch bonds. Instead, our data suggest that catch bonds formed by agonists, such as OT‐I/OVA, will have unusually fast k_off_ so that they can only function as agonists by forming a catch bond that sufficiently slows their k^m^_off_ to induce TCR signals. The structural explanation for the x_β_‐k_off_ relationship described above cannot easily explain catch bonds and why they may be more common with low‐affinity interactions. One possible explanation is that lower affinity TCR/pMHC complexes possess more conformational flexibility within the binding interface, increasing the likelihood of conformational adjustments leading to new interactions during unbinding under force.

We have previously shown that the adhesion molecules CD2 and LFA‐1 improve T‐cell antigen discrimination. Here, we have found that the kinetic proofreading mechanism, when modified to include the effects of force, can explain the effect of CD2 and LFA‐1 on antigen discrimination by their ability to shield the TCR/pMHC interaction from forces. This is readily explained in the case of CD2 because CD2/CD58 interactions span the same distance as the TCR/pMHC interaction. This size compatibility enables them to closely colocalize with individual TCR/pMHC interactions within close contacts and hence share forces (Fig 4I, left and middle panels). Indeed, modelling confirms that receptor/ligand interactions can share forces when co‐localized on the nanometre scale (Pullen & Abel, 2017; Różycki & Weikl, 2021). Although CD2 increases discrimination by the 1G4 TCR, a recent study suggests that it decreases discrimination by the OT‐I TCR (Li *et al*, 2022). These data are consistent with our finding that, unlike the 1G4 TCR, the purified OT‐I TCR forms catch bonds with its agonist pMHC. Thus, force‐shielding by CD2 would accelerate dissociation of agonist pMHC from the OT‐I TCR. We note that signalling by CD2 (λ in our model) can increase antigen potency irrespective of the slip or catch nature of the bond. The recent demonstration that the binding of CD2 to its ligand on the same cell can enhance T‐cell activation by pMHC supports a role for CD2 signalling, since cis interactions would not provide force shielding of the TCR/pMHC interaction (Li *et al*, 2022). Taken together, this can explain why the net effect of CD2 on both the OT‐I and 1G4 is to increase potency but with a different magnitude depending on affinity.

Given that the LFA‐1/ICAM‐1 interaction spans a greater distance than the TCR/pMHC interaction (Springer, 1990) and does not intimately colocalize with it within the immunological synapse (Hashimoto‐Tane *et al*, 2016), it cannot reduce TCR/pMHC forces by the same CD2/CD58 force shielding mechanism. Instead, we suggest that LFA‐1/ICAM‐1 interactions reduce force on a larger scale, by acting as a diffusional barrier to exclude large glycocalyx molecules such as CD43 and CD45 (Allard *et al*, 2012; Freeman *et al*, 2016), and through their ability to reduce T‐cell mobility and/or stabilize lamellipodia and microvilli processes (e.g. Fig 4I, right panel). In support of this, LFA‐1/ICAM‐1 interactions have been shown to form “micro‐adhesion” rings surrounding areas of TCR/pMHC engagement (Hashimoto‐Tane *et al*, 2016). Furthermore, LFA‐1/ICAM‐1 interactions experience substantial forces (> 56 pN) at T‐cell interfaces, and increasing their ability to withstand forces enhances antigen discrimination (Ma *et al*, 2022). Direct measurements of the effect of engaging adhesion receptors on the force experienced by the TCR at T‐cell/APC interfaces are needed to test these predictions.

Our study has several limitations. First, the LFC assay cannot accurately apply forces below ~ 4 pN and therefore we cannot rule out catch bonds at forces below 4 pN. Second, forces were only applied along the normal TCR/pMHC binding axes, and therefore, we cannot rule out catch bonds with forces applied in other directions. It has been suggested that the TCR/pMHC interaction can be subjected to shear forces (Göhring *et al*, 2021). Third, we have used purified TCRαβ domains whereas in T cells the TCRαβ domains are intimately associated with CD3 signalling subunits. Therefore, it would be interesting to repeat the LFC assay using purified TCR‐CD3 complex and/or to repeat the BFP assays using TCR‐CD3 presented on membranes rather than live T cells.

In conclusion, we have shown that force sensitivity increases with affinity, which implies that force on the TCR/pMHC interaction impairs antigen discrimination. The fact an antibody/antigen interaction shows the same correlation suggests that this may be general feature of antigen receptors. Importantly, B‐cell antigen recognition also occurs at cellular interfaces where mechanical forces are likely to play an important role (Natkanski *et al*, 2013). Given the critical role of T and B cell antigen discrimination, we suggest that force shielding is functionally important as it provides an environment where antigen receptors experience a consistent level of force. The fact that CD2 and LFA‐1 are expressed on all T cells, and their ligands CD58 and ICAM‐1 are ubiquitously expressed, makes them suitable for such roles.

## Materials and Methods

### Laminar flow chamber assay

Glass slides were rinsed twice in absolute ethanol then washed in a “piranha” solution composed of 70% H_2_SO_4_, 15% water and 15% H_2_O_2_ for 10 min, then rinsed with 5 litres of deionized water. Glass slides were coated with poly‐L‐lysine (150–300 kDa, Sigma Aldrich, France) at 100 μg/ml in a 0.01 M phosphate solution, pH 7.4 for 10 min, then rinsed in PBS, then incubated with glutaraldehyde (2.5% in pH 9.5 0.1 M borate solution, Sigma Aldrich) for 10 min, then rinsed in PBS, then incubated in a 100 μg/ml biotinylated BSA solution in PBS (Sigma Aldrich), for 30 min, then rinsed in PBS, then incubated in a 10 μg/ml streptavidin solution in PBS (Sigma Aldrich), then rinsed in PBS. Glass slides were then mounted in a home‐made multi‐channel thermo‐regulated flow chamber device, forming nine independent chambers of 12 mm length and 2 × 0.250 mm^2^ section. Each chamber was then filled with a biotinylated pMHC solution in PBS with 0.1% BSA at chosen concentration, using cascade dilution to deposit in our 9‐chambers apparatus 8 different amounts of pMHC plus one chamber as a negative control. Microspheres (Dynal M‐450 tosly‐activated, Thermo Fisher, France) were rinsed three time in a 0.1 M borate solution, then incubated in a 200 μg/ml solution of anti‐6xHis‐tag antibody (Bio‐Rad, MCA1396, clone AD1.1.10, RRID: AB322084) overnight under constant agitation. Microspheres were rinsed three time in PBS/0.1% BSA then incubated prior to each experiment in a 200 μg/ml solution of 6xHis‐tag TCR.

Flow chamber experiments were performed using our automaton based on a Arduino Mega 2560 card (Arduino, Italy). The device forming nine independent chambers on a common glass slide was thermo‐regulated at 37°C by water circulation, set on an inverted microscope with a 10× lens (Leica, Germany) with a digital CCD camera (UEye, IDS Germany), and chamber entry was connected to the piping. For each independent chamber, the automaton performed cycles, each cycle being an experiment for a given shear flow. The automaton repeated cycles of microspheres agitation, microspheres injection in the chamber, launch of movie recording at 50 frames per seconds with M‐JPEG on‐the‐fly compression, and flow at a given shear for 90 s. Shear value was automatically modified for each new cycle until all chosen shear conditions had been recorded. The chamber was manually disconnected and next chamber was connected, then automaton was re‐launched. Raw data are in the form of movies of microspheres motion. A software suite written in Java as ImageJ plug‐ins retrieves microspheres trajectories and then detects microspheres arrest events using a velocity threshold and record arrests number, arrests duration and distance travelled by sedimented microspheres.

Binding linear densities are calculated as the ratio of the number of arrests on the distance travelled by microspheres sedimented on the chamber surface. Specific binding densities are calculated by subtracting control linear binding density from assay linear binding density for a given shear condition. Duration of arrests was pooled for experiments sharing identical TCR and pMHC molecules, identical amount of pMHC on the surface and identical shear rate, to build survival curves of the arrests. Data points that did not have more events than the corresponding control experiment were excluded. Each TCR‐pMHC bond was measured in 8 to 12 independent experiments. Single molecular bond observation was assessed using the usual flow chamber arguments: in an interval of deposited amounts of pMHC, linear binding density was increasing linearly from negative control value with the mount of deposited ligand; survival curves of bonds would not change in the same range of amounts of deposited pMHC, showing observation of similar binding events. For this binding density analysis, specific survival curves were calculated by subtracting, for each time step, the corresponding survival fraction of non‐specific arrests measured in control experiments.

The TCR, pMHC and the concentrations that were used are as follows:

1G4/NYE 9V/SLLMWITQV/0.25 mg/ml, 1G4/NYE 3A/SLAMWITQV/1.25 mg/ml, 1G4/NYE 4E5E8E/SLLEEITEV/5.00 mg/ml, 1G4/NYE 6V/SLLMWVTQV/0.50 mg/ml, 1G4/NYE 3Y/SLYMWITQV/1.00 mg/ml, 1G4/NYE 4D/SLLDWITQV/0.50 mg/ml, 1G4/NYE 6T/SLLMWTTQV/1.00 mg/ml, A6/Tax WT/LLFGYPVYV/0.13 mg/ml, A6/Tax 5F/LLFGWPVYV/1.00 mg/ml, A6/Tax 7T/LLFGYPTYV/5.00 mg/ml, A6/Tax 7R/LLFGYPRYV/0.25 ug/ml, A6/Tax 7Q/LLFGYPQYV/1.25 mg/ml, A6/Tax 5H/LLFGHPVYV/1.00 mg/ml, OT‐I/OVA/SIINFEKL/0.55 mg/ml.

### Data analysis

For each experiment, the survival and binding frequency was determined. Data from multiple experiments with small variations in velocity was pooled into the following velocity bins: [2, 10], [10, 20], [20, 40], [40, 60], [60, 80], [80, 120] μm/s. The average velocity was used to calculate the force. Data points were excluded from further analysis if the binding linear density of a sample was not different than the no pMHC control. The fold‐change was calculated for each velocity category, data points with a geo. mean ‐ geo. SEM of less than 1 were excluded (indicating that the binding events are mostly unspecific). Data were corrected for unspecific binding by subtracting no pMHC control data from the same velocity bin. The survival and binding frequency data was analysed using a custom Python pipeline (Python 3.9.7 AMD64, lmfit 1.0.3, matplotlib 3.4.3) and GraphPad Prism 9.4.1 (GraphPad Software). Experiments with less than 15 events in the interval (1 s, 2 s] were excluded. We used 3 different approaches to analyse the data. For each time point with at least one recorded event, the survival fraction was calculated. To calculate off‐rates, we determined the survival fraction at 1 s and 2 s: k_off_ = (−ln [s(t)])/t. In the rare cases where there was no event at exactly 1 s or 2 s, we used a subsequent event. Lastly, we fit a 1‐phase exponential curve with 3 free parameters to the first 5 s of the corrected survival data: s (t) = (A − B) exp (−k_off_ t) + B, where t is the time in seconds, B the baseline plateau and A the amplitude.

We used the resulting off‐rates to fit Bell's model (24) to each TCR/pMHC where visual inspection indicated that the data would follow such an exponential model: k_off_ (F) = k^0^_off_ exp (Fx_β_/k_B_T), where F is the force in pN, k^0^_off_ the zero‐force off‐rate (s^−1^; fitted parameter), x_β_ the force sensitivity (nm; fitted parameter), k_B_ the Boltzmann constant (0.0138 pN nm/K), and T the temperature (310.15 K).

### Protein expression and purification

Soluble OT‐I TCR construct consisted of the murine variable OT‐I domain and the human constant domain truncated above the trans‐membrane domain with an artificial interchain disulphide. Soluble 1G4 TCR (no artificial disulphide) and A6 TCR (with an artificial disulphide) were similarly truncated above the trans‐membrane domain (TRAC residue 93, TRBC2 residue 129). All TCRs contained a 6xHis‐tag on one chain to allow immobilization on beads for force experiments. TCR α and β chains were expressed in BL21 (DE3)‐RIPL Escherichia coli cells (Agilent Technologies) following induction with 0.15 mM IPTG. Inclusion bodies were isolated by disrupting cells with BugBuster (Merck) according to the manufacturer's instructions. Proteins were stored at −80°C until use. TCRs were refolded by adding 15 mg (OT‐I) or 30 mg (1G4 or A6) of each chain dropwise in 1 L cold refolding buffer (0.15 M Tris–HCl pH 8.0, 3 M urea, 0.2 M Arg‐HCl, 0.5 mM EDTA), followed by dialysis for 3 days in 10 L dialysis buffer (10 mM Tris–HCl pH 8.5), with a buffer change after day 1. After dialysis, the protein was filtered and purified using anion‐exchange chromatography (HiTrap Q column [GE Healthcare]) with a NaCl gradient, followed by concentration and purification by size exclusion chromatography (Superdex 200 column [GE Healthcare]) in HBS‐EP (0.01 M HEPES pH 7.4, 0.15 M NaCl, 3 mM EDTA, 0.005% v/v Tween20). TCR purity was checked by SDS–PAGE and concentrations were measured with a Nanodrop spectrophotometer (Thermo Fisher). Purified TCR were stored at 4°C and used for SPR and GCI measurements withing 24 h (OT‐I) or 1 month (1G4 and A6) after purification to avoid aggregation.

Soluble class I pMHCs bound to OVA peptide (OVA257–264; SIINFEKL) or A2 variant (SAINFEKL) were refolded and biotinylated by the NIH protein facility. We used mouse H‐2Kb heavy chain and human beta‐2 microglobulin, biotinylated on the C terminal of the heavy chain. pMHCs were aliquoted and stored at −80°C until use.

NYE (NYE‐ESO157–165) and Tax (HTLV‐1 Tax11–19) class I pMHCs were refolded in‐house. The heteroclitic 9V variant was used as index peptide for the 1G4 TCR, rather than the wild‐type 9C, due to its improved stability on MHC (Chen *et al*, 2005). Soluble human HLA‐A*0201 heavy chain (UniProt residues 25–298) with a C‐terminal AviTag/BirA recognition sequence and human beta‐2 microglobulin were expressed in Escherichia coli and isolated from inclusion bodies. Trimer was refolded by consecutively adding peptide, β2M and heavy chain into refolding buffer and incubating for 2–3 days at 4°C. Protein was filtered, concentrated using centrifugal filters, biotinylated (BirA biotin‐protein ligase bulk reaction kit [Avidity, USA]) and purified by size exclusion chromatography (Superdex 75 column [GE Healthcare]) in HBS‐EP. Purified protein was aliquoted and stored at −80°C until use.

Soluble extracellular domain (ECD) of human CD58 (UniProt residues 29–204 + AviTAG + 6xHis) and human CD86 (UniProt residues 24–238 + AviTAG + 6xHis) were produced in Freestyle 293F suspension cells (Thermo Fisher) according to the manufacturer's instructions. Ligands were biotinylated by co‐transfection (1:10) of a secreted BirA‐encoding plasmid (pTT3‐BirA‐FLAG) and adding 100 μM D‐biotin to the medium, as described previously (Parrott & Barry, 2001). All supernatants were 0.45 μm filtered and 100 μm PMSF was added. Proteins were purified using standard Ni‐NTA agarose columns. Proteins were further purified by size exclusion chromatography (Superdex 75 or 200 column [GE Healthcare]) in HBS‐EP; purified proteins were aliquoted and stored at −80°C until use.

### Surface plasmon resonance

TCR–pMHC interactions were analysed on a Biacore T200 instrument (GE Healthcare Life Sciences) at 37°C and a flow rate of 10 μl/min. Running buffer was HBS‐EP. Streptavidin was coupled to CM5 sensor chips using an amino coupling kit (GE Healthcare Life Sciences) to near saturation, typically 10,000–12,000 response units (RU). Biotinylated pMHCs (47 kDa) were injected into the experimental flow cells (FCs) for different lengths of time to produce desired immobilization levels (typically 500–1,500 RU), which were matched as closely as feasible in each chip. Usually, FC1 was a reference for FC2–FC4. Biotinylated CD58 ECD (24 kDa + 25 kDa glycosylation) was immobilized in FC1 at a level matching those of pMHCs. In some experiments, another FC was used as a reference. Excess streptavidin was blocked with two 40 s injections of 250 μM biotin (Avidity). Before injections of soluble 1G4 or A6 TCR (51 kDa), the chip surface was conditioned with 8 injections of the running buffer. Dilution series of TCRs were injected simultaneously in all FCs; the duration of injections (30–70 s) was the same for conditioning and TCR injections. After every 2–3 TCR injections, buffer was injected to generate data for double referencing. After the final TCR injection and an additional buffer injection, W6/32 antibody (10 μg/ml; BioLegend; Lot: B233942) was injected for 10 min.

TCR steady‐state binding was measured > 10 s post‐injection. In addition to subtracting the signal from the reference FC with immobilized CD58 (single referencing), all TCR binding data were double‐referenced vs. the average of the closest buffer injections before and after TCR injection. This allows to exclude small differences in signal between flow cells (e.g. drifts). TCR binding versus TCR concentration was fitted with the following model: B = Bmax [TCR]/(K_D_ +[TCR]), where B is the response/binding, Bmax the maximal binding (this parameter is either kept free or is fixed with the W6/32 derived Bmax), and [TCR] the injected TCR concentration. Maximal W6/32 binding (Rmax) was used to generate the empirical standard curve and to infer the Bmax of TCRs from the standard curve. Rmax was derived by fitting the W6/32 binding data after double referencing with the following, empirically chosen, model: R = Rmax t/(K_t_ + t), where t is time (s), R the sensogram response after single referencing, and K_t_ a nuisance parameter. The empirical standard curve only contained data where the ratio of the highest concentration of TCR to the fitted K_D_ value (obtained using the standard method with Bmax fitted) was 2.5 or more. This threshold ensured that the binding response curves saturated so that only accurate measurements of Bmax were included. All interactions were fit using both the fitted and constrained Bmax method. For constrained K_D_ above 20 μM we reported the constrained K_D_, otherwise we use the Bmax fitted K_D_. SPR data were analysed using GraphPad Prism 8 and 9 (GraphPad software) or using a custom Python script (Python v3.7 and lmfit v0.9.13).

### Surface plasmon resonance and grating‐coupled interferometry experiments for OT‐I TCR

Binding properties of OT‐I TCR interaction with OVA were measured by SPR on a Biacore S200 and T200 (GE Healthcare Life Sciences) using a CM5 sensor chip and by GCI on the WAVEsystem (Creoptix) with a 4PCP sensor chip. HBS‐EP was used as running buffer and all measurements were performed at 37°C. For protein immobilization, the sensor chip was saturated with streptavidin using an amine coupling kit (GE Healthcare Life Sciences). Biotinylated pMHCs were immobilize at various levels (100–300 RU for kinetics, 1,000–2,000 RU for affinity measurements). CD86 with matching immobilization levels were used as reference protein. Excess streptavidin was blocked with two 40 s injections of 500 μM biotin (Avidity), and the sensor was conditioned with at least eight injections of running buffer. TCR concentrations used varied between 50–150 μM.

Binding affinities were measured by equilibrium binding on a T200 Biacore instrument. TCR was injected at increasing concentrations at 30 μl/min with flow path 1‐2‐3‐4. Buffer was injected after every 2–3 TCR injections. K_D_ values were obtained by fitting a 1:1 Langmuir binding model (RUeq = RUmax [TCR]/(K_D_ + [TCR])) to double‐referenced equilibrium RU values.

For kinetic measurements by SPR we used a Biacore S200. Different TCR concentrations were injected at a flow rate of 30 μl/min. To minimize diffusion artifacts, TCR was injected separately in flow path 1–2 and 3–4. We obtained k_off_ by fitting a mono‐exponential to double‐referenced dissociation curves.

For kinetic measurements by GCI, we used a Creoptix waveRAPID. A single TCR concentration was injected multiple times using different length pulses for a total duration of 5 s, followed by a 50 s dissociation phase. To calibrate how the analyte (TCR) concentration changes over time during pulse injection, 0.5% DMSO is injected. Flow rate was set to of 100 μl/min per flow cell. We obtained k_off_ by fitting a mono‐exponential to double‐referenced dissociation curves.

### Coarse‐grained molecular dynamics simulations

We have used an implicit‐solvent coarse‐grained structure‐based model in which amino acid residues are represented by single beads (Różycki *et al*, 2014). The beads are tethered together into chains by harmonic springs. Non‐local interactions between the beads are introduced on the basis of the PDB structure of the protein complex under study. Specifically, interactions between the beads that form contacts in the PDB structure of the protein complex are described by the Lennard‐Jones potential. Interactions between the beads that do not form contacts in the PDB structure are purely repulsive and short‐ranged. Disulphide bonds are captured by harmonic springs between the specified Cys beads. Importantly, the amino acid contacts in PDB structures are identified using an overlap criterion applied to the coordinates of all heavy atoms in the structures. The pairs of amino acid residues that are very close sequentially, that is (i,i + 1) and (i,i + 2), are excluded from the set of contacts in the PDB structure. A detailed description of the coarse‐grained structure‐based model is given in (Różycki *et al*, 2014).

As input to our simulations, we used the PDB structures of the 1G4 and A6 TCR‐pMHC complexes with the PDB codes 2BNQ and 3QFJ, respectively. We considered five constructs of pMHC in complex with the 1G4 TCR, where (i) all contacts between the peptide and the TCR identified in the PDB structure (PDB code: 2BNQ) were included in the coarse‐grained model, (ii) contacts between the peptide residues 4 to 8 and the TCR identified in the PDB structure were excluded from the coarse‐grained model, (iii) only contacts between the peptide and the TCRa identified in the PDB structure were taken into account in the coarse‐grained model, (iv) only contacts between the peptide and the TCRβ identified in the PDB structure were taken into account in the coarse‐grained model, and (v) no contacts between the peptide and the TCR identified in the PDB structure were included in the coarse‐grained model. We also studied four constructs of pMHC in complex with the A6 TCR, where (i) all contacts between the peptide and the TCR identified in the PDB structure (PDB code: 3QFJ) were included in the coarse‐grained model, (ii) only contacts between the peptide and the TCRα identified in the PDB structure were taken into account in the coarse‐grained model, (iii) only contacts between the peptide and the TCRβ identified in the PDB structure were taken into account in the coarse‐grained model, and (iv) no contacts between the peptide and the TCR identified in the PDB structure were included in the coarse‐grained model.

We performed molecular dynamics simulations of the coarse‐grained model using the Langevin thermostat. All of the simulations were started from native states corresponding to the PDB structures. Stretching of the TCR‐pMHC complexes was implemented by attaching harmonic springs to two beads: the first one to the C‐terminus of the TCRβ and the second one to the C‐terminus of the MHC. The first of the springs was fixed in space and the second one was moved with a constant speed v. We performed the simulations with v ranging from 0.0005 to 0.005 Å/t, where the time unit t is estimated to be of the order of 1 ns (Szymczak & Cieplak, 2006). To prevent unfolding of the individual chains within the TCR‐MHC complexes, we replaced the intra‐molecular contacts by harmonic springs. The inter‐molecular contacts, however, were still described by the Lennard‐Jones potentials, as previously introduced (Różycki *et al*, 2014). This modification resulted in F(t) traces with single peaks (Fig 3A and B) that were identified to coincide with TCR‐pMHC dissociation events. We monitored the response force F acting on the pulling spring (Fig 3A) and determined the average force Fmax at which the TCR‐pMHC complexes break apart. The average was obtained from 20 independent trajectories (Fig 3B). The dependence of Fmax on v follows the Bell‐Evans formula (Schwesinger *et al*, 2000): F = (k_B_T / x_β_)ln (x_β_κ v / (k_B_T k_off_)), which yields the values of parameters k_off_ and x_β_ (Fig 3C). Here, k_B_ is the Boltzmann constant, T is the absolute temperature, and κ = 0.12 k_B_T/Å^2^ is the stiffness of the harmonic springs attached to the C‐termini of MHC and TCRβ (the product of v and κ equals to the loading rate).

### Kinetic proofreading with molecular forces

All calculations in Fig 4 were performed with the kinetic proofreading model modified to include the membrane off‐rate under force. At steady‐state, the concentration of signalling TCR in state N is calculated to be, C_N_ = (1 + k^m^_off_ (F)/k_p_)^−*N*^ C_T_ where, CT = (1/2) (L_0_ + R_0_+ k^m^_off_ (F)/k_on_ − ((L_0_ + R_0_+ k^m^_off_ (F)/k_on_)^2^ − 4L_0_R_0_)^1/2^). In this model, L_0_ and R_0_ are the total concentration of pMHC and TCR, respectively, k_on_ is the on‐rate and k^m^_off_ (F) is the membrane off‐rate under force (F). We used the empirical relationship determine for the 1G4 TCR for all calculations (Fig 2F), k^m^_off_ (F) = k_on_K_D_ exp ((−0.029log_10_(K_D_)–0.11)F/4.2797), where k_on_ was taken to be 0.0447 μM^−1^ s^−1^, which is the average k_on_ value for the subset of pMHCs where kinetic parameters were available (Pettmann *et al*, 2021). We fixed the value of *N* to 2.46 and k_p_ to 2.15 s^−1^ to the value we obtained for the plate data when providing adhesion ligands (fig 5 in Pettmann *et al*, 2021). Potency (P) was defined as the concentration of pMHC (L_0_) required to achieve a value of C_N_ of 10^−4^. Antigen sensitivity was defined as the value of P for the highest affinity antigen and antigen discrimination was defined as the logarithm of the fold‐change in P over the logarithm of the fold‐change in k_off_ for the highest affinity ligand over the lowest affinity ligand in Fig 4C.

To directly fit the model to experimental potency data, we solved for the concentration of pMHC required to obtain a threshold value of CN (see Pettmann *et al*, 2021 for complete derivation), P = λ + *N* log (1 + k^m^_off_ (F)/k_p_), where λ is proportional to the threshold value of C_N_, *N* is the number of proofreading steps, k_p_ is the rate of each step, and k^m^_off_ (F) is defined above. In this fit, the only free parameters were F and λ. We fit the model to the plate data (pMHC alone, pMHC + CD58, and pMHC + ICAM‐1) using Prism (GraphPad) using a different value of F for each condition (local force) and either a single value of λ (global threshold) or a different value of λ for each condition (local threshold).

## Author contributions

**Johannes Pettmann:** Conceptualization; data curation; formal analysis; investigation; visualization; methodology; writing – original draft; writing – review and editing. **Lama Awada:** Data curation; investigation; formal analysis; methodology; writing – original draft; writing – review and editing. **Bartosz Różycki:** Formal analysis; investigation; visualization; data curation; methodology; writing – review and editing. **Anna Huhn:** Data curation; formal analysis; investigation; writing – review and editing. **Sara Faour:** Data curation; formal analysis; investigation. **Mikhail Kutuzov:** Data curation; formal analysis; investigation; writing – review and editing. **Laurent Limozin:** Investigation; writing – review and editing. **Thomas R Weikl:** Formal analysis; investigation; writing – review and editing. **P Anton van der Merwe:** Supervision; investigation; writing – original draft; writing – review and editing. **Philippe Robert:** Conceptualization; data curation; formal analysis; supervision; funding acquisition; investigation; visualization; methodology; writing – original draft; project administration; writing – review and editing. **Omer Dushek:** Conceptualization; supervision; funding acquisition; investigation; visualization; writing – original draft; project administration; writing – review and editing.

## Disclosure and competing interest statements

The authors declare that they have no conflict of interest.

## Supporting information



Appendix

Expanded View Figures PDF

Movie EV1

Source Data for Expanded View and Appendix

PDF+

Source Data for Figure 2

Source Data for Figure 3

Source Data for Figure 4

## Data Availability

The molecular dynamics data of this article are available in the open research data repository Edmond at http://doi.org/10.17617/3.2VLJ6X
