# Supplementary material for: Mechanical forces impair antigen discrimination by reducing differences in T‐cell receptor/peptide–MHC off‐rates
Source: EMBO J. 2022 Dec 9;42(7):e111841. doi: 10.15252/embj.2022111841 (PMC10068313; doi:10.15252/embj.2022111841)
Supplement: Supplementary file 1 — Appendix [file EMBJ-42-e111841-s001.pdf]

Appendix

Table of Contents

Appendix Figures - Page 2-14

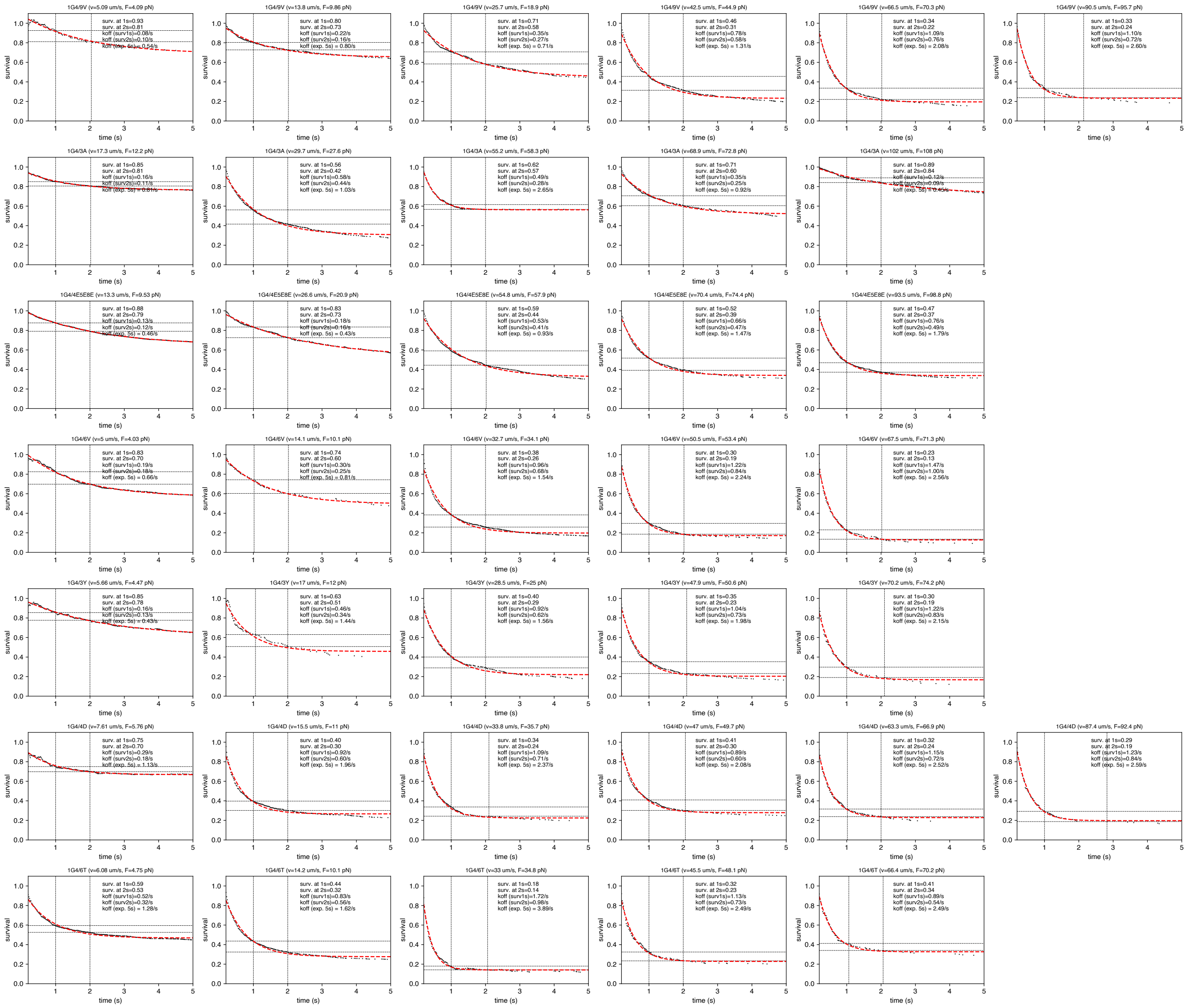

Appendix Figure S1. Survival analysis for the 1G4 TCR with different pMHCs at different flow velocities/forces.
The applied force increases from left to right across the columns and each row is a different pMHC (indicated in panel title). The off-rate is estimated by the survival at 1 s, survival at 2 s, or by an exponential fit of the first 5 s (values are indicated). See main text and methods for details.

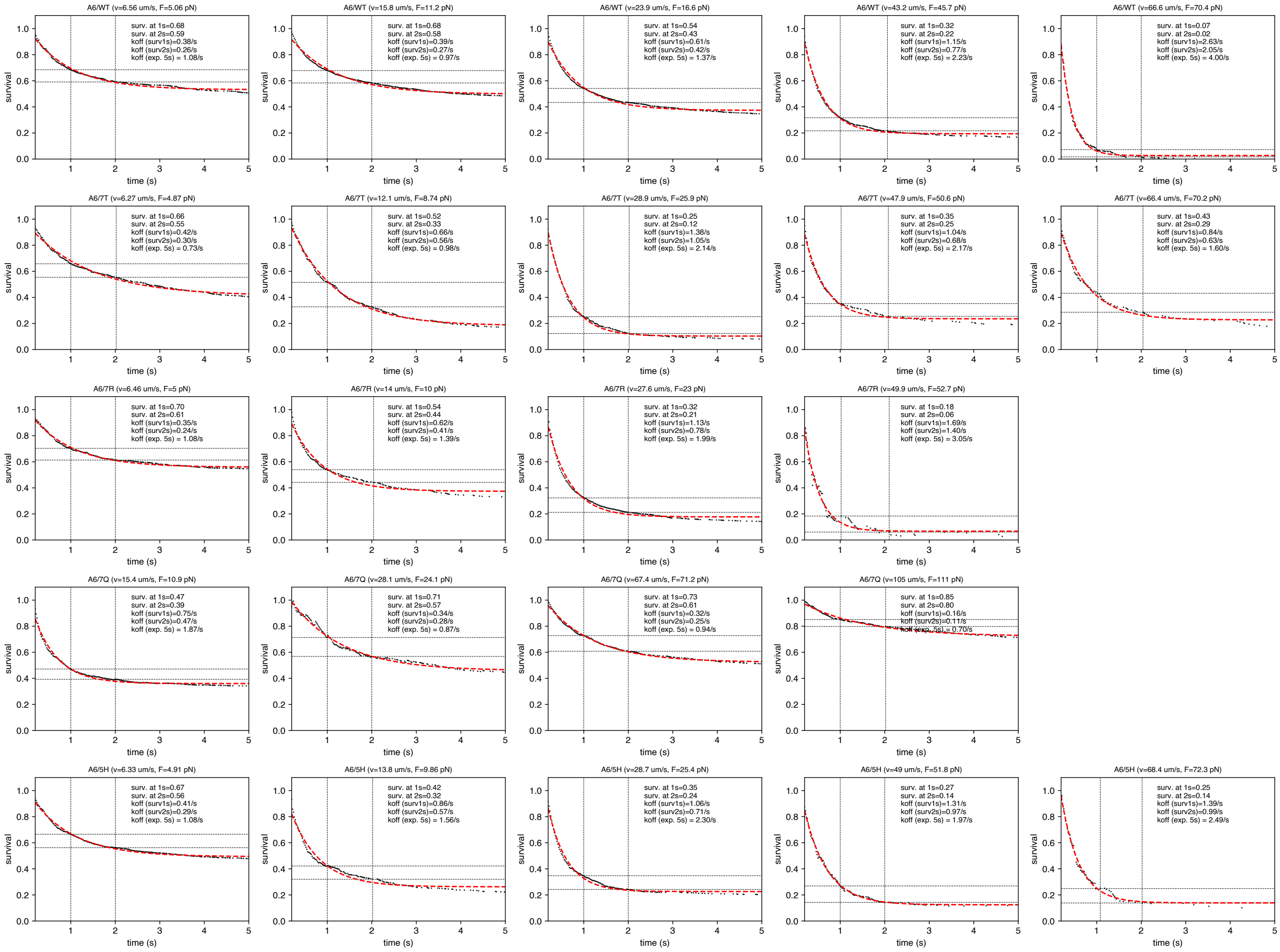

Appendix Figure S2. Survival analysis for the A6 TCR with different pMHCs at different flow velocities/forces. The applied force increases from left to right across the columns and each row is a different pMHC (indicated in panel title). The off-rate is estimated by the survival at 1 s, survival at 2 s, or by an exponential fit of the first 5 s (values are indicated). See main text and methods for details.

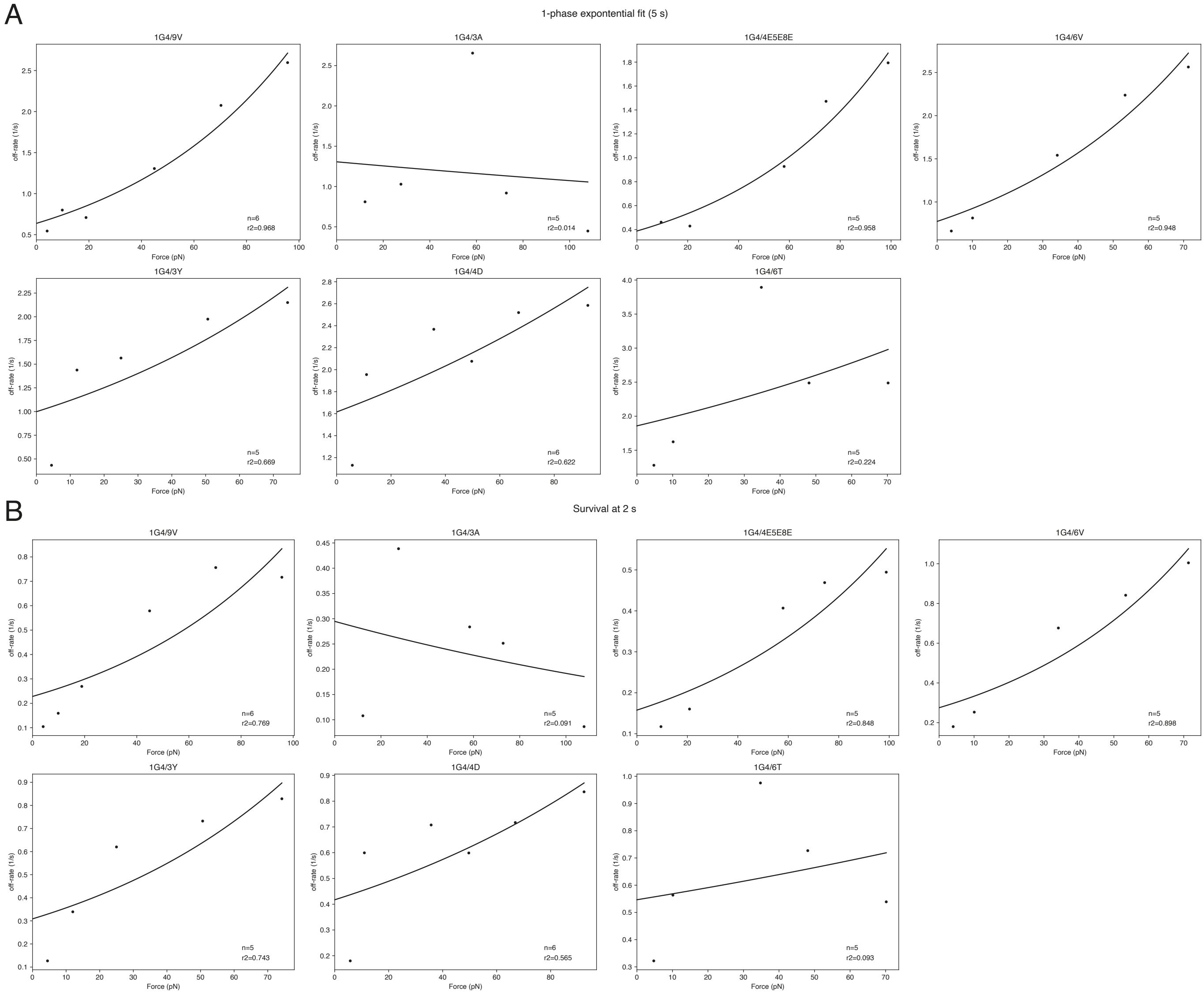

Appendix Figure S3. Off-rate over force for the 1G4 TCR fitted with Bell's model using off-rates calculated by (A) exponential fit or (B) survival at 2 s (see Appendix Figure S1).

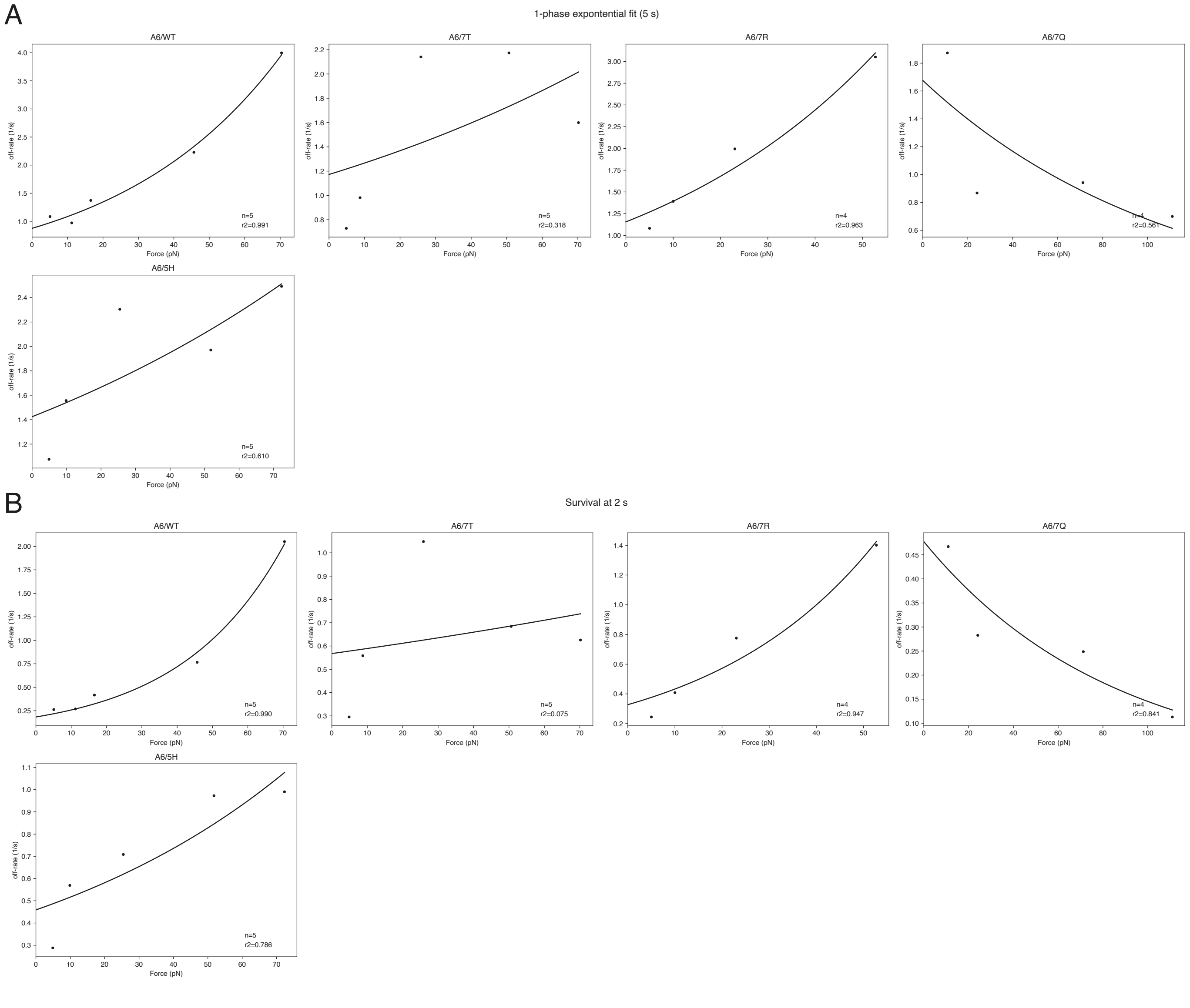

Appendix Figure S4. Off-rate over force for the A6 TCR fitted with Bell's model using off-rates calculated by (A) exponential fit or (B) survival at 2 s (see Appendix Figure S2).

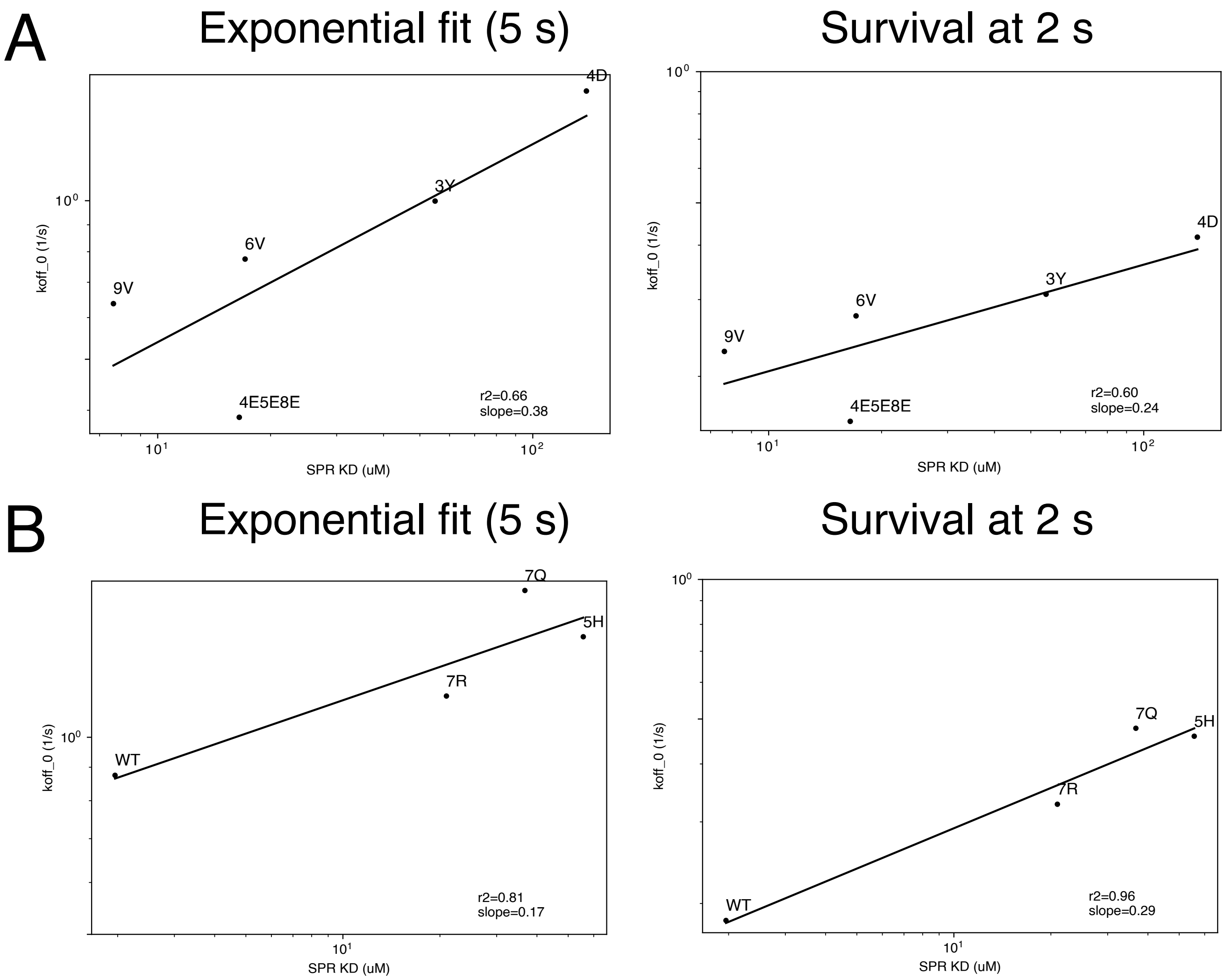

Appendix Figure S5. Correlations between zero-force off-rate determined by Bell's model using two different analysis methods (Appendix Figure S1 & Figure S2)) and the SPR K_D for the (A) 1G4 and (B) A6 TCR.

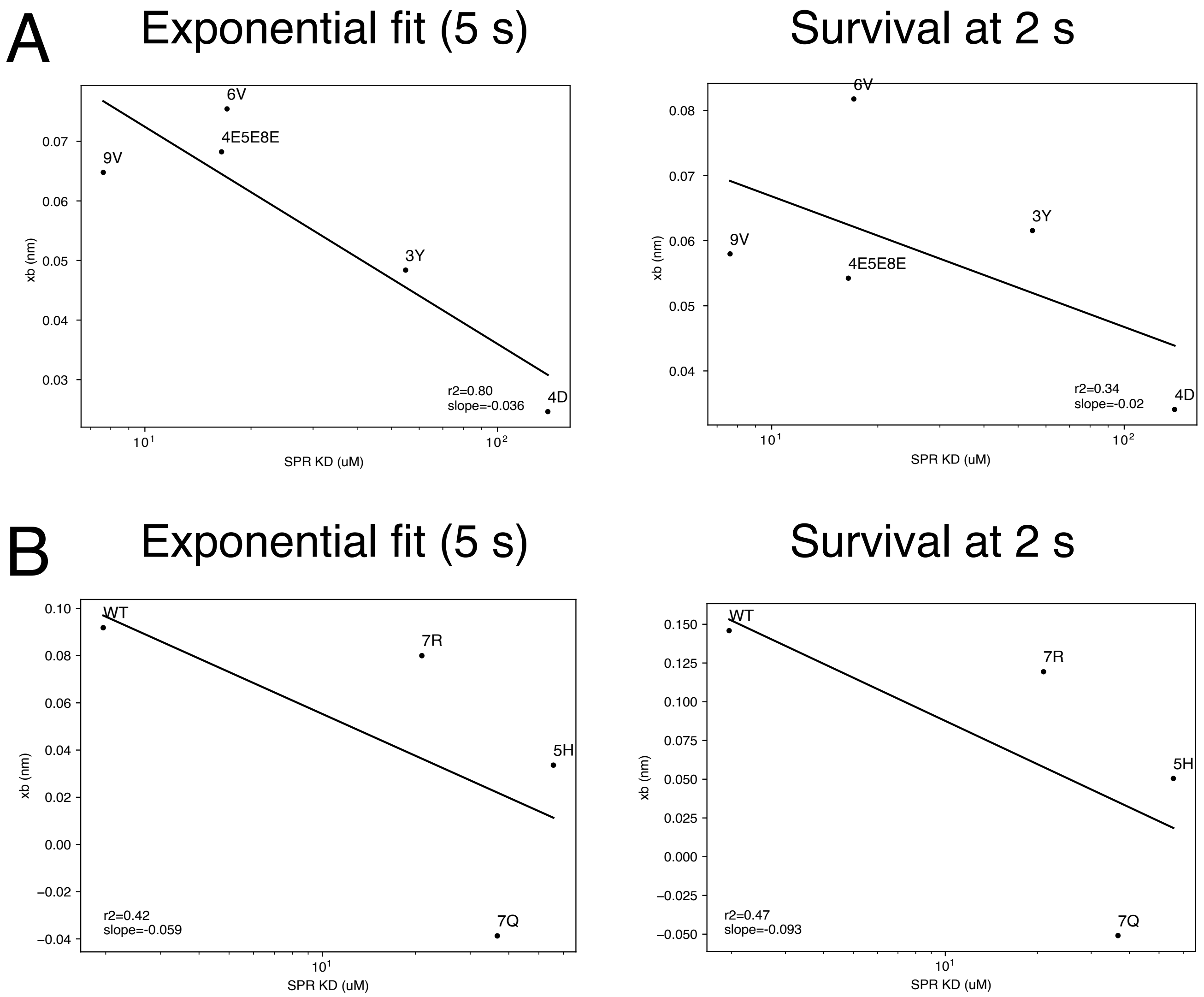

Appendix Figure S6. Correlations between the force sensitivity parameter (x_β) determined by Bell's model using two different analysis methods (Appendix Figure S1 & Figure S2)) and the SPR K_D for the (A) 1G4 and (B) A6 TCR.

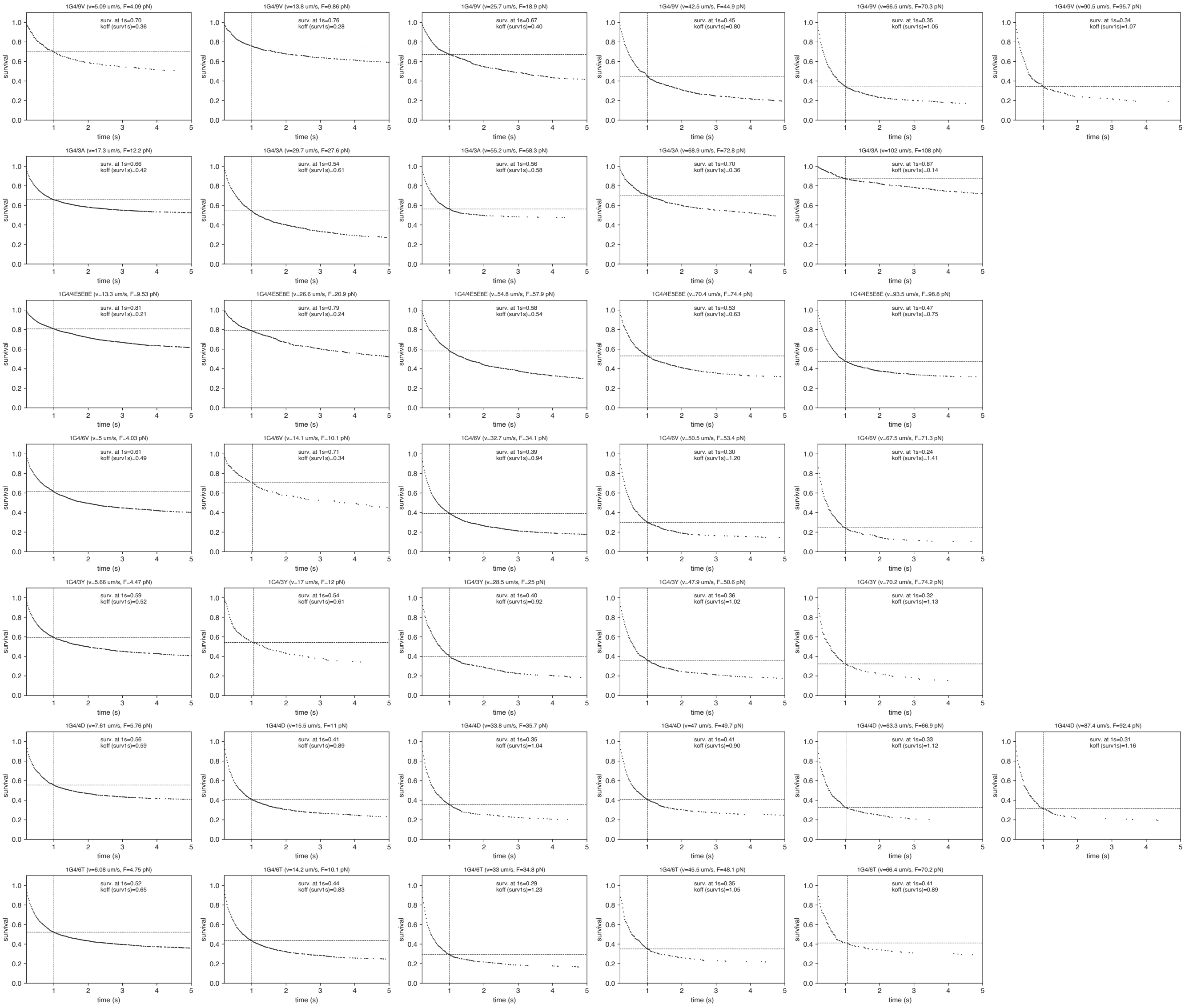

Appendix Figure S7. Raw survival curves for the 1G4 TCR (without correction for non-specific binding).
Figure analogous to Appendix Figure S1 except the survival curves have not been corrected for non-specific binding.

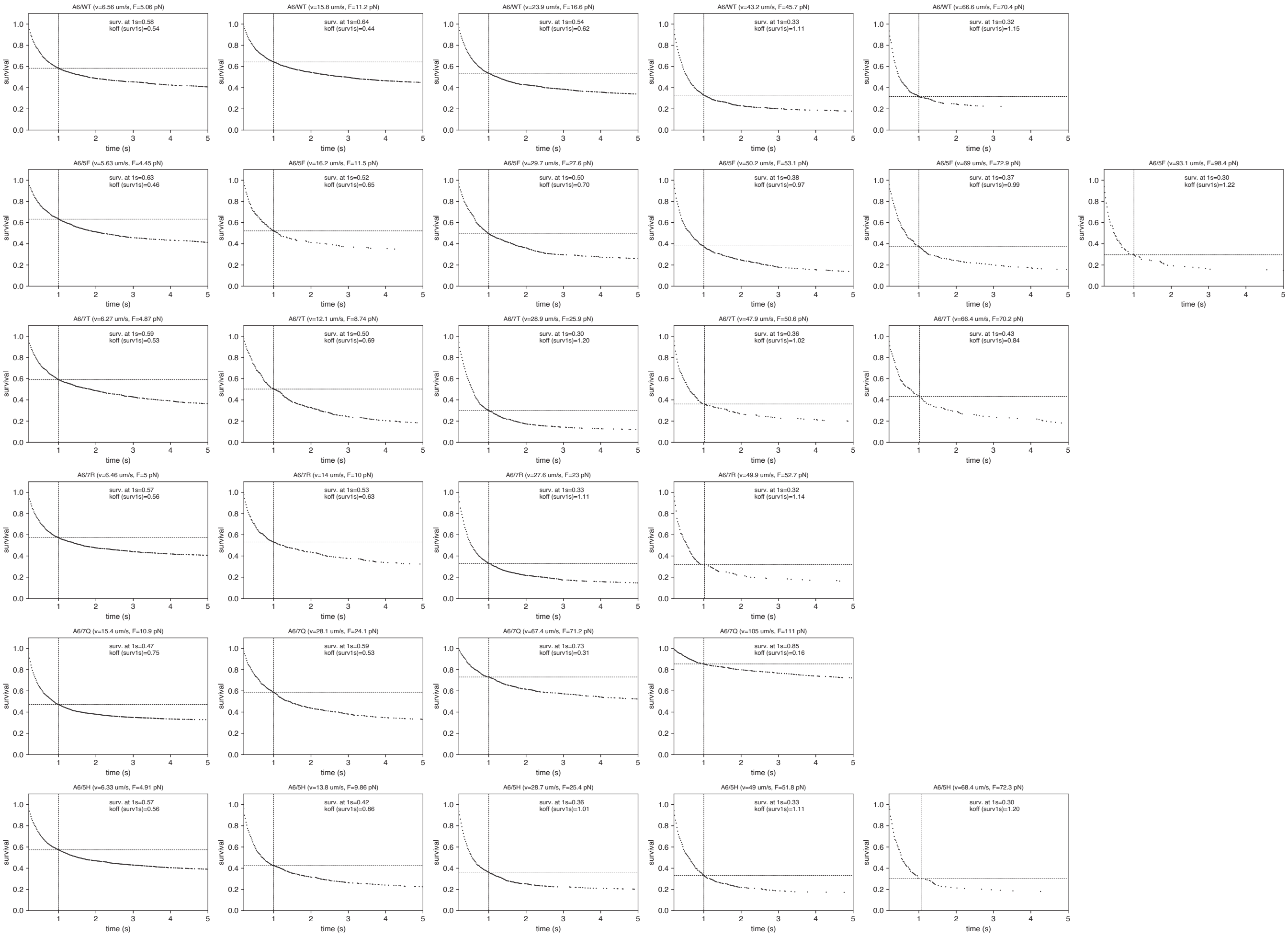

Appendix Figure S8. Raw survival curves for the A6 TCR (without correction for non-specific binding).
Figure analogous to Appendix Figure S2 except the survival curves have not been corrected for non-specific binding.

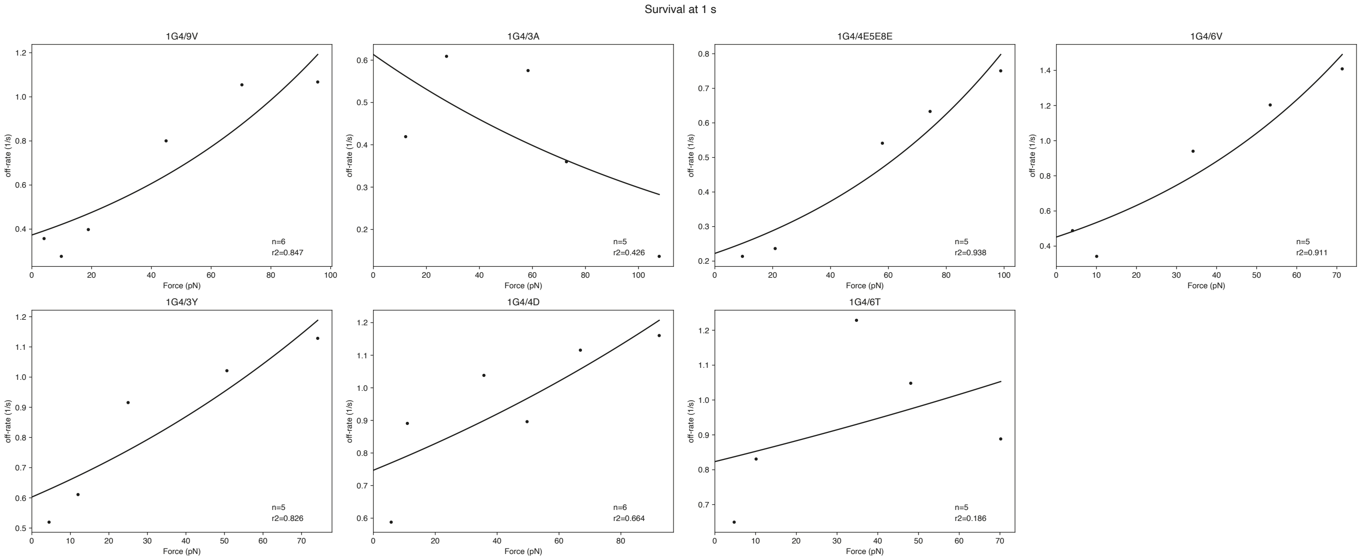

Appendix Figure S9. Off-rate over force for the 1G4 TCR fitted by Bell's model using data from Appendix Figure S7 (without correction for non-specific binding).
Off-rates were calculated from survival at 1 s.

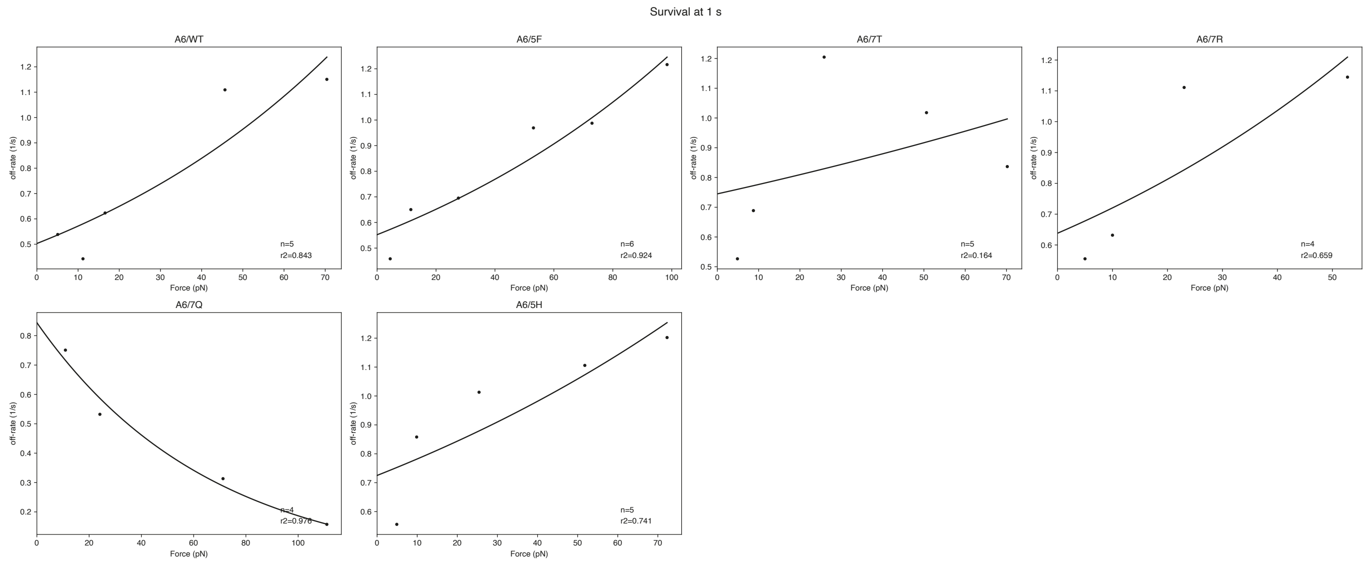

Appendix Figure S10. Off-rate over force for the A6 TCR fitted by Bell's model using data from Appendix Figure S8 (without correction for non-specific binding).

Off-rates were calculated from survival at 1 s.

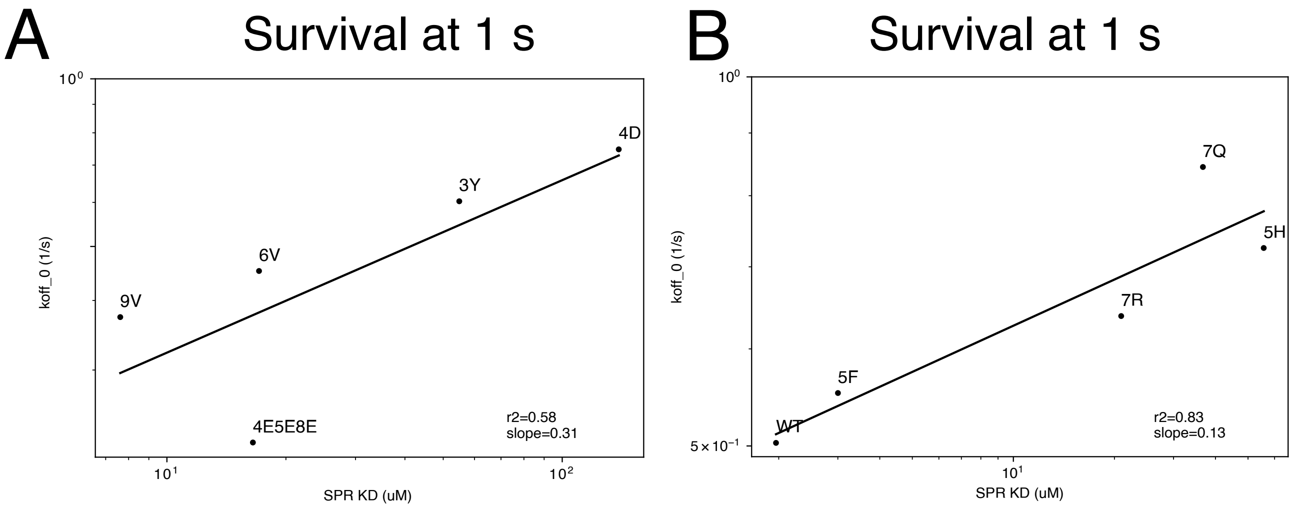

Appendix Figure S11. Correlations between zero-force off-rate and SPR K_D for the (A) 1G4 and (B) A6 TCRs (without correction for non-specific binding, see Appendix Figure S9 & Figure S10).

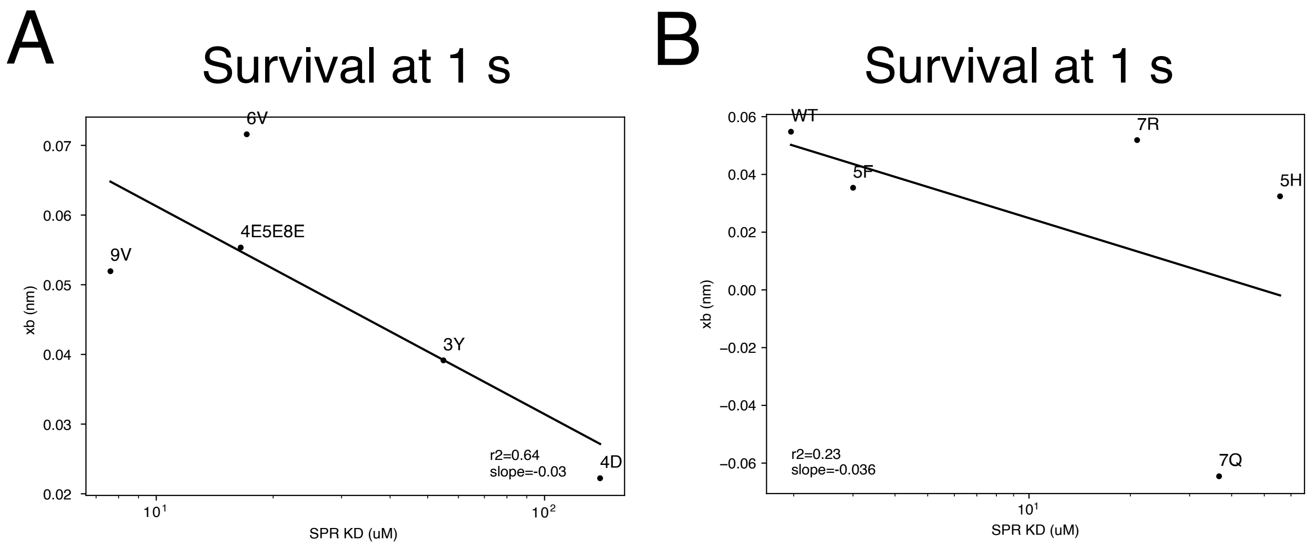

Appendix Figure S12. Correlations between the force sensitivity x_β and SPR K_D for the (A) 1G4 and (B) A6 TCRs (without correction for non-specific binding, see Appendix Figure S9 & Figure S10).

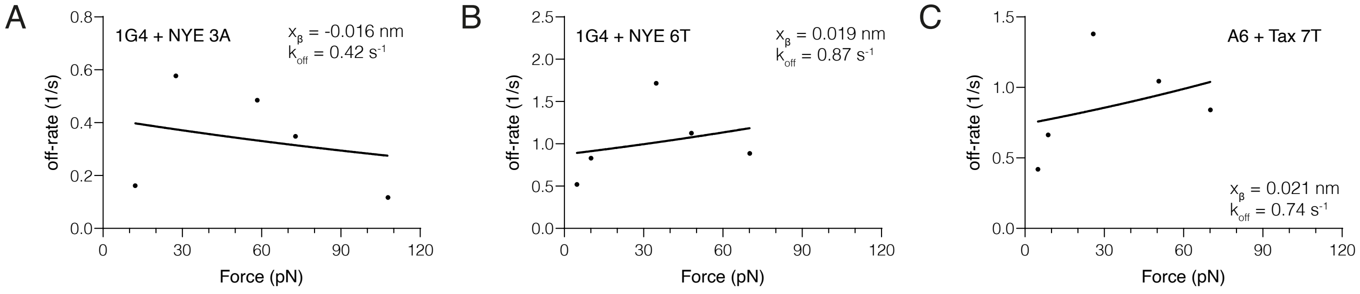

Appendix Figure S13. Non-canonical TCR/pMHC bonds.
(A–C) Data fitted with Bell's model. Since all of them did not show a classical force-response, as predicted by Bell's model, we excluded them from any correlation. Notably, they all exhibit slip bond behavior at low forces. The number of independent experiments that were combined to produce the estimated off-rates are: 9 (1G4/3A), 9 (1G4/6T), 11 (A6/7T). Data shown is from fitting off-rates obtained from the survival at 1 s analysis.

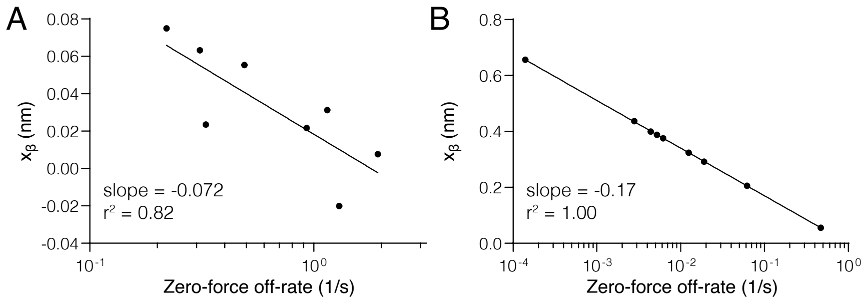

Appendix Figure S14. Negative x_β k_{off} correlation is reproduced in two other studies.
(A) We previously generated data at 25 ℃ with a similar correlation using the 1G4 TCR and peptide variants, as well as, MHC molecules with different mutations (Robert et al., 2012). **(B)** Schwesinger et al. found a negative correlation for different antibodies binding the same antigens when measured by atomic force microscopy (Schwesinger et al., 2000).

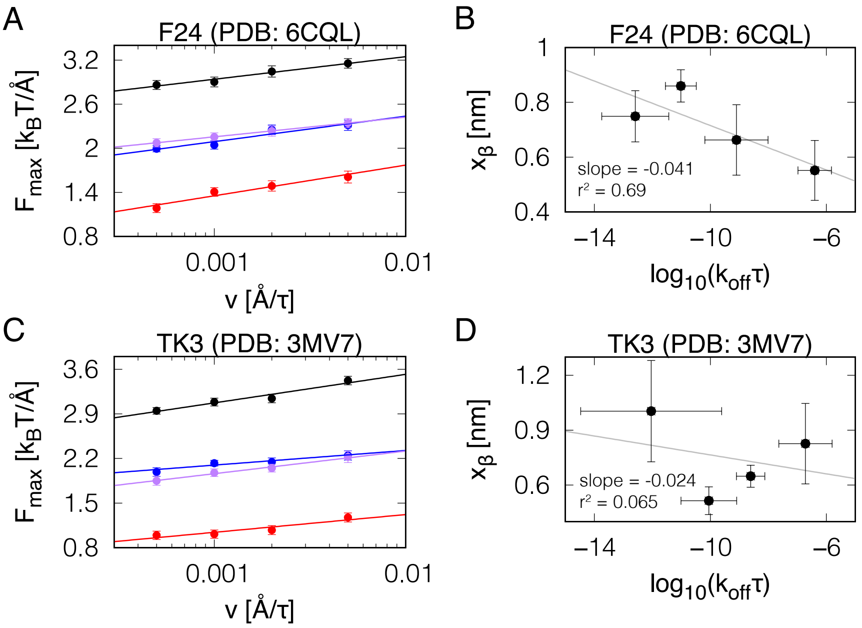

Appendix Figure S15. Negative x_β k_{off} correlation is reproduced in two other TCRs.
Analogous to Figure 3D–G but for two other pMHCs in complexes with the F24 TCR **(A–B)** and the TK3 TCR **(C–D)**. The negative correlation between x_β and log(k_{off}) is observed also for these TCR-pMHC complexes (B and D).

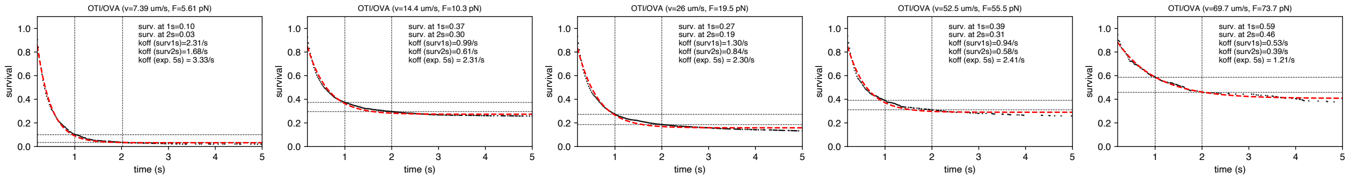

Appendix Figure S16. Survival analysis of the OT-I TCR interacting with the OVA pMHC under different applied forces.
The applied force increases from left to right (indicated in panel title). The off-rate is calculated using the survival at 1 s, survival at 2 s, or an exponential fit.

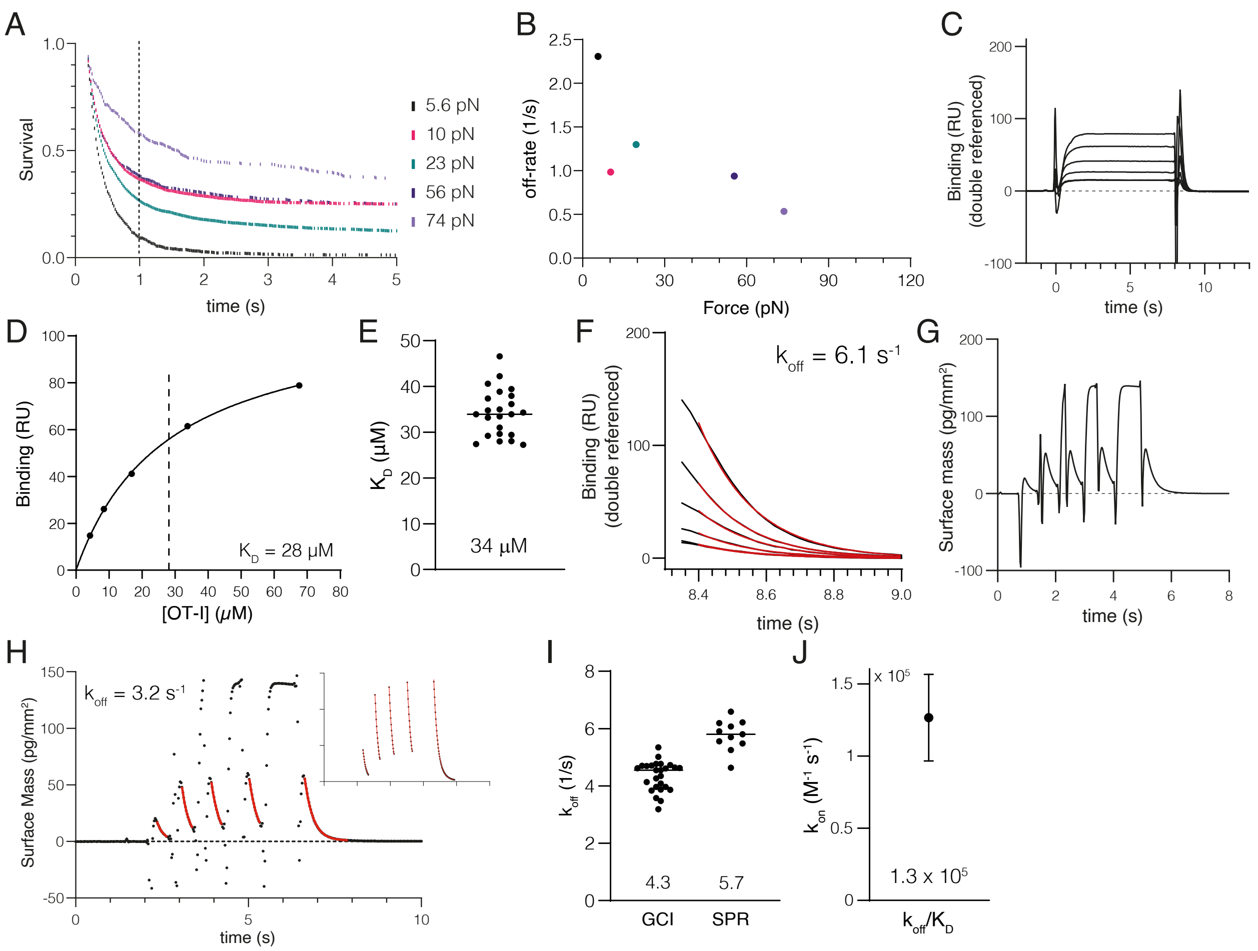

Appendix Figure S17. The OT-I TCR binds OVA with low affinity and a catch bond.
(A) Example of bead survival over time at different velocities/forces. The time to measure survival (1 s) is shown as dotted line. **(B)** Off-rates at different applied forces. Colors correspond to forces in (A). The number of independent experiments performed on different days that were combined to produce the estimated off-rates are N=11. **(C)** Representative surface plasmon resonance (SPR) sensogram of OT-I TCR binding immobilized OVA pMHC at five different concentrations of TCR. **(D)** Steady-state RU from (A) plotted over the TCR concentration fitted by a one-site specific binding model to determine K_D (solid line). **(E)** Fitted K_D values from multiple experiments (N=23). **(F)** Dissociation phase from (A) fitted with a one phase exponential decay model to determine k_{off} (solid red lines). **(G)** Representative grating-coupled interferometry (GCI) sensogram of OT-I TCR binding immobilized OVA pMHC at one concentration of TCR injected for increasing durations. **(H)** Fit of the entire sensogram data from (G) determined k_{off} (red line). **(I)** Fitted k_{off} values from four and two independent experiments with different TCR and/or pMHC concentrations using GCI (N=26) or SPR (N=11), respectively. **(J)** Calculated on-rate (k_{on}) using the fitted values of k_{off} (4.3 s^{-1}) and K_D (34 μM).

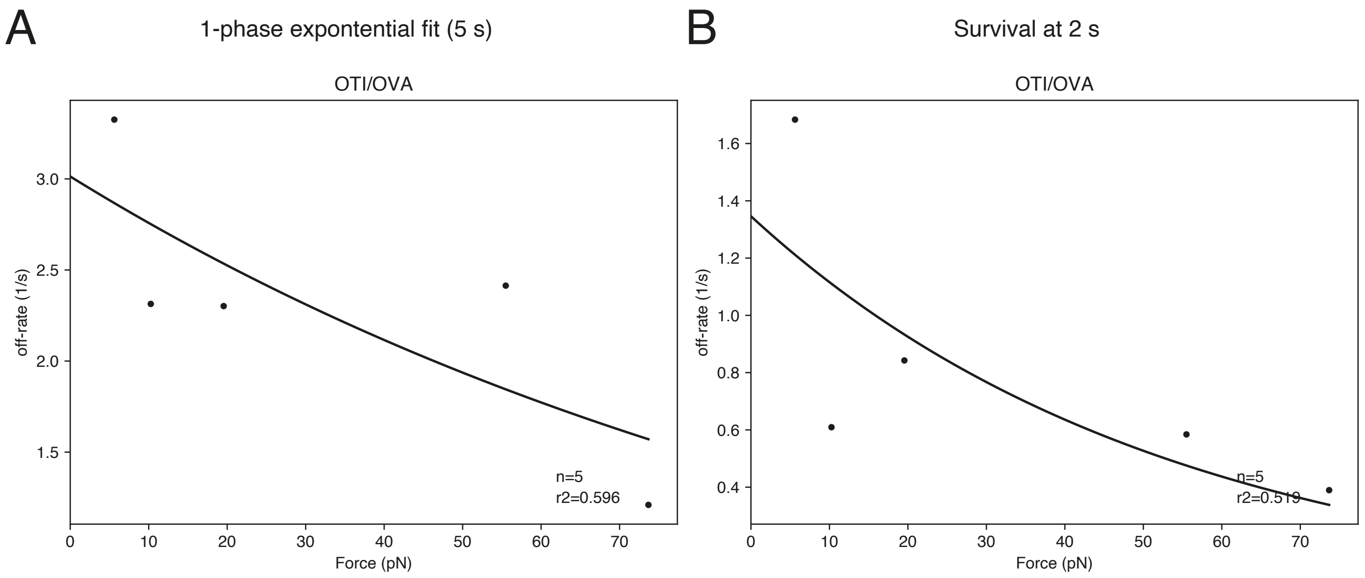

Appendix Figure S18. Off-rate over force for the OT-I/OVA interaction using two different methods to calculate the off-rate. The off-rate is calculated using **(A)** an exponential fit or **(B)** survival at 2 s. The off-rates are taken from Appendix Figure S16. The off-rate calculated using survival at 1 s is shown in Appendix Figure S17B.
