# Supplementary material for: Mechanical forces impair antigen discrimination by reducing differences in T‐cell receptor/peptide–MHC off‐rates
Source: EMBO J. 2022 Dec 9;42(7):e111841. doi: 10.15252/embj.2022111841 (PMC10068313; doi:10.15252/embj.2022111841)
Supplement: Supplementary file 2 — Expanded View Figures PDF [file EMBJ-42-e111841-s005.pdf]

Expanded View Figures

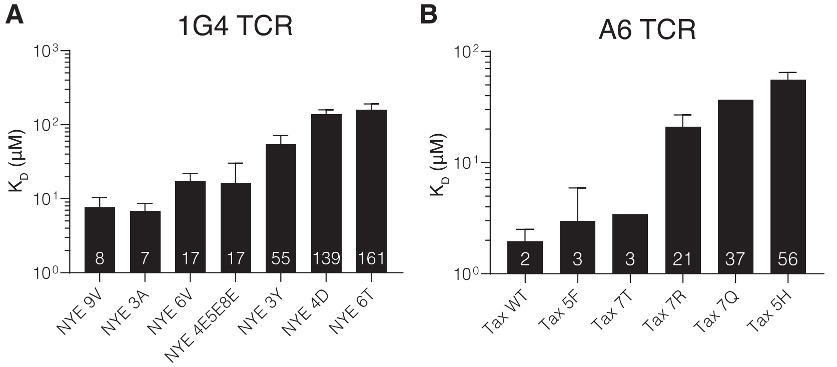

Figure EV1. Dissociation constants of TCR/pMHC bond measured by SPR.

A SPR dissociation constants for the 1G4 TCR (N = 2–10).

B SPR dissociation constants for the A6 TCR (N = 1–6).

Data information: Shown are geometric means with geo. SDs from the N independent experiments conducted on different days with the numbers in the bar indicating the geometric mean. Data is partially reproduced from Fig 1G and H of our previous measurements (Pettmann *et al*, 2021) with additional repeats and pMHC variants.
Source data are available online for this figure.

 © 2022 The Authors

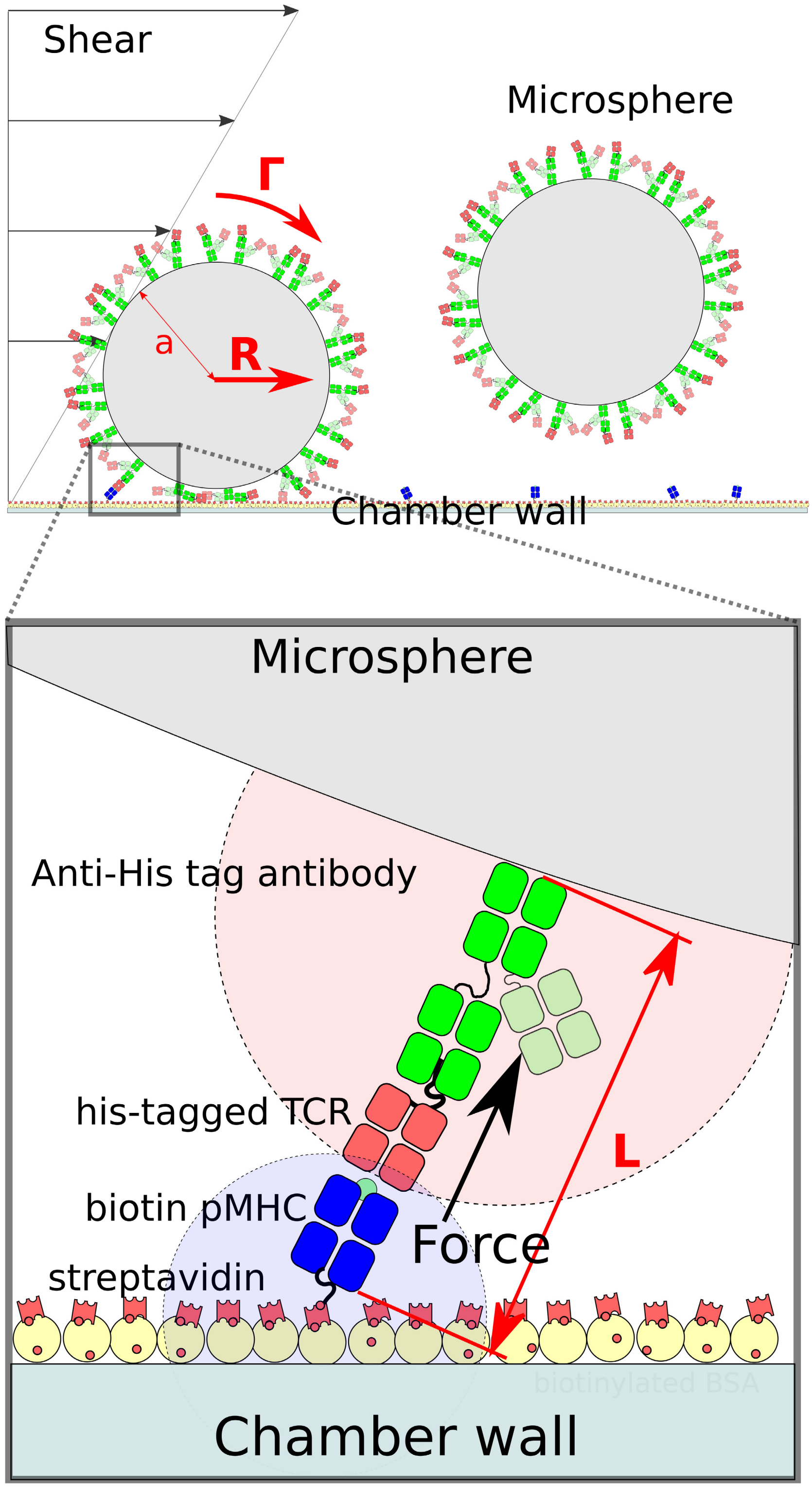

Figure EV2. Flow velocity resolves into a normal force on the TCR/pMHC as a result of anchoring flexibility.

Biotinylated pMHC and antibody-bound TCR have rotational freedom as a result of a flexible linker between the avi-tag and the pMHC and between the TCR and the his-tag that the antibody binds. This allows their binding site to diffuse in volumes represented by potions of disc in light blue for the pMHC and light red for TCR. Near to the surface of the chamber, the flow velocity is given by a first-order approximation: $v(z) = Gz$, where z is the distance to the surface in nm and G is the shear rate constants in s^{-1}. The shear stress T_s (in N/m^2 or pascals) applied corresponds to the force applied by the flow per surface unit and is calculated by the product between G and the viscosity of the medium (μ) in Pa/s: $T_s = \mu G$. Taking into account the dimensions of the LFC section lxH, shear rate can be determined for a given flow Q using the formula: $G \approx 6Q/H^2$. When the microsphere makes a link with the surface the flow creates a hydrodynamic force (R) given by the equation: $R \approx 32\mu a^2 G = 1.7005 \times 6\pi\mu a^2 G$ where a is the radius of the microsphere. The microsphere is also subjected to a torque force (Γ): $\Gamma \approx 0.9440 \times 4\mu a^3 G$. In addition, a lever effect increases the force applied to the interaction, so the total force applied (F) is given by the equation: $F \approx (R + \Gamma/a)\sqrt{(a/2L)}$ where L is the length of the formed bond, that is, the length of the link. Additional details can be found in (Pierres *et al*, 1995).

Figure EV3. Linear binding density correlation indicates assay operation in single molecule bond regime.

A Linear binding densities for all tested TCR/pMHC combinations. A linear correlation indicates a single-molecule binding regime.
B Example of survival curves of the same TCR/pMHC interaction with different pMHC immobilization levels. Shown is 1G4/NYE 6T.

Data information: Data points in (A) represent mean and SD from independent experiments using 1G4 TCR binding NYE 9V ($N = 7$), NYE 3A ($N = 9$), NYE 6V ($N = 8$), NYE 3Y ($N = 10$), NYE 4D ($N = 6$), NYE 6T ($N = 9$), NYE 4E5E8E ($N = 8$) and A6 TCR binding Tax WT ($N = 9$), Tax 5F ($N = 11$), Tax 7T ($N = 11$), Tax 7R ($N = 8$), Tax 7Q ($N = 9$), Tax 5H ($N = 8$), and the OT-I TCR binding OVA ($N = 11$).
Source data are available online for this figure.

 © 2022 The Authors

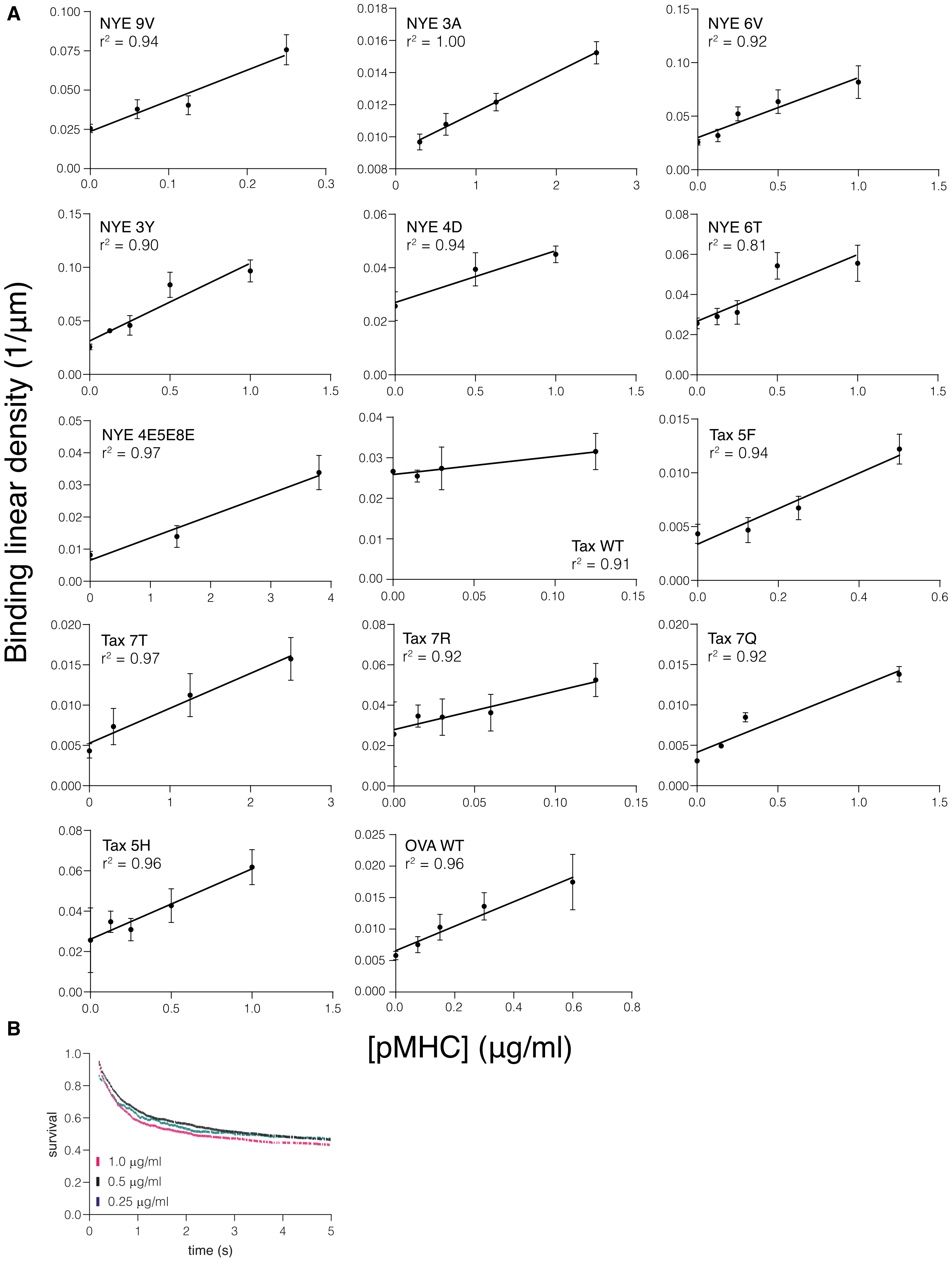
A
NYE 9V
r^2 = 0.94
NYE 3A
r^2 = 1.00
NYE 6V
r^2 = 0.92
NYE 3Y
r^2 = 0.90
NYE 4D
r^2 = 0.94
NYE 6T
r^2 = 0.81
NYE 4E5E8E
r^2 = 0.97
Tax WT
r^2 = 0.91
Tax 5F
r^2 = 0.94
Tax 7T
r^2 = 0.97
Tax 7R
r^2 = 0.92
Tax 7Q
r^2 = 0.92
Tax 5H
r^2 = 0.96
OVA WT
r^2 = 0.96
Binding linear density (1/µm)
[pMHC] (µg/ml)
B
survival
time (s)
1.0 µg/ml
0.5 µg/ml
0.25 µg/ml

Figure EV3.

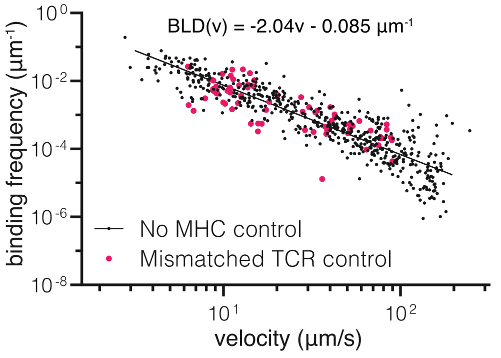

Figure EV4. Binding linear density for control surfaces over flow velocity.

Binding linear density (BLD) of TCR-coated beads over surfaces without pMHC (black – no MHC control) or surfaces with a pMHC that does not match the TCR on the bead (pink – mismatch). Each dot represents one experiment/flow cell. Mismatched control is pooled data from experiments with 1G4/OVA, 1G4/Tax WT, 1G4/Tax 7R, A6/NYE 4D, A6/NYE 6V, and A6/OVA. No pMHC control was fitted with a linear curve in loglog space.

Source data are available online for this figure.

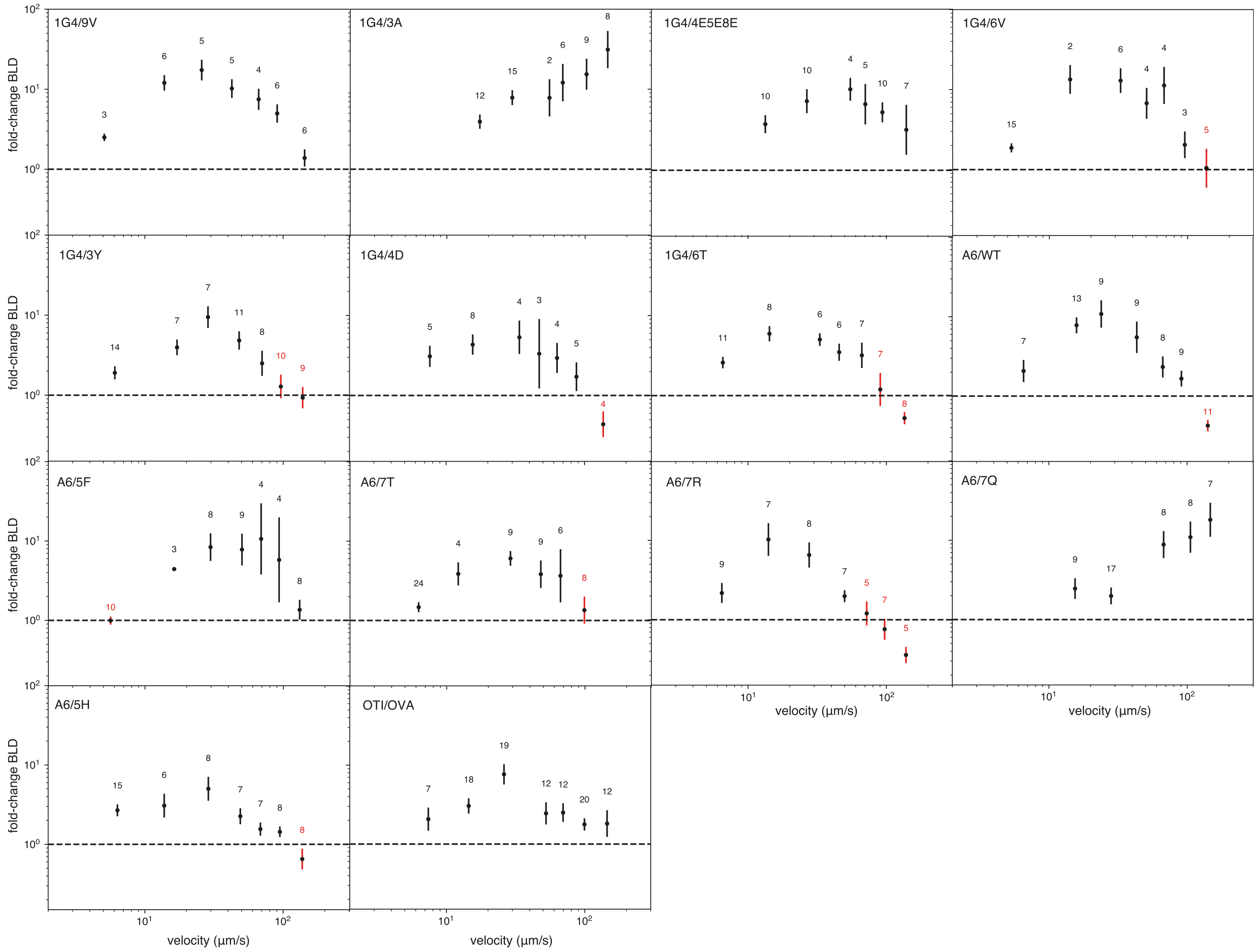

Figure EV5. Specific TCR/pMHC interactions show higher binding linear densities at most flow velocities.

The ratio of BLD between the indicated samples and the no pMHC control. Points mark the geometric mean with geo. SEM at different velocity bins. Velocity categories where the lower error bar < 1 were excluded (red). The number of experiments binned in each velocity category is indicated above each data point.

Source data are available online for this figure.

 © 2022 The Authors
