# Supplementary material for: Mechanical forces impair antigen discrimination by reducing differences in T‐cell receptor/peptide–MHC off‐rates
Source: EMBO J. 2022 Dec 9;42(7):e111841. doi: 10.15252/embj.2022111841 (PMC10068313; doi:10.15252/embj.2022111841)
Supplement: Supplementary file 3 — Movie EV1 [file EMBJ-42-e111841-s002.zip › Movie EV1.docx]

**Movie EV1. Automated algorithm detects bead arrests.** An example of microspheres coated with the A6 TCR injected over a surface coated with the 7T pMHC using flow velocities that generate 7 pN force on the TCR/pMHC interaction. The movie is in real-time with 50 fps showing a 640x320 subframe of a 2048x544 pixel field of view. Green crosses indicate arrests detected with a start and end whereas red crosses indicate arrests detected with a start but with uncertainty with the end (and hence duration) because it reaches the end of the movie or because of interrupted tracking (e.g. collision with another bead). Arrests are only included when the bead velocity prior to arrest is consistent with the flow velocity for the particular condition ensuring that data is included only when the applied force can be verified.
