# Supplementary material for: Mechanical forces impair antigen discrimination by reducing differences in T‐cell receptor/peptide–MHC off‐rates
Source: EMBO J. 2022 Dec 9;42(7):e111841. doi: 10.15252/embj.2022111841 (PMC10068313; doi:10.15252/embj.2022111841)
Supplement: Supplementary file 9 [file EMBJ-42-e111841-s003.pdf]

THE EMBO JOURNAL

Mechanical forces impair antigen discrimination by reducing differences in T cell receptor/peptide-MHC off-rates

Johannes Pettmann, Lama Awada, Bartosz Różycki, Anna Huhn, Sara Faour, Mikhail Kutuzov, Laurent Limozin, Thomas Weikl, Anton van der Merwe, Philippe Robert, and Omer Dushek
DOI: 10.15252/embj.2022111841

Corresponding author(s): Omer Dushek (omer.dushek@path.ox.ac.uk) , Anton van der Merwe (anton.vandermerwe@path.ox.ac.uk), Philippe Robert (philippe.robert@inserm.fr)

Review Timeline:

Submission Date:	8th Jun 22
Editorial Decision:	16th Aug 22
Revision Received:	5th Oct 22
Editorial Decision:	3rd Nov 22
Revision Received:	8th Nov 22
Accepted:	16th Nov 22

Editor: Karin Dumstrei

Transaction Report:

(Note: With the exception of the correction of typographical or spelling errors that could be a source of ambiguity, letters and reports are not edited. Depending on transfer agreements, referee reports obtained elsewhere may or may not be included in this compilation. Referee reports are anonymous unless the Referee chooses to sign their reports.)

1st Editorial Decision 16th Aug 2022

Dear Omer,

Thank you for submitting your point-by-point response. I have now had a chance to take a look at it. I appreciate the proposed changes and would like to invite you to submit a revised manuscript.

When preparing your letter of response to the referees' comments, please bear in mind that this will form part of the Review Process File, and will therefore be available online to the community. For more details on our Transparent Editorial Process, please visit our website: https://www.embopress.org/page/journal/14602075/authorguide#transparentprocess

I thank you for the opportunity to consider your work for publication. I look forward to your revision.

Yours sincerely,

Karin Dumstrei, PhD
Senior Editor
The EMBO Journal

Instructions for preparing your revised manuscript:

Guide For Authors: https://www.embopress.org/page/journal/14602075/authorguide

I have attached a PDF with helpful tips on how to prepare the revised version.

We realize that it is difficult to revise to a specific deadline. In the interest of protecting the conceptual advance provided by the work, we recommend a revision within 3 months (14th Nov 2022).

As a matter of policy, competing manuscripts published during this period will not negatively impact on our assessment of the conceptual advance presented by your study. However, please contact me as soon as possible upon publication of any related work, to discuss how to proceed.

If you require more time to complete the revisions let me know as as I can grant an extension.

Use the link below to submit your revision:

https://emboj.msubmit.net/cgi-bin/main.plex

Referee #1:

"Mechanical forces impair antigen discrimination by reducing differences in T cell receptor off-rates", by Pettmann et al., combines computational modeling with cell free biophysical measurements to investigate the force sensitivity of several TCR-pMHC interactions. The authors find, unexpectedly, that most of the interactions exhibit slip bond behavior and that higher affinity interactions are especially sensitive to the application of force. These observations run counter to prior results from the biomembrane force system. The authors then apply molecular dynamics simulations to validate the slip bond behavior and force sensitivity of several TCR-pMHC pairs of interest. In an interesting aside, they demonstrate that the OT-1 TCR, long thought to recognize its cognate pMHC with high affinity, is actually a low affinity binder with a very fast off-rate, and that similar to some other low affinity binders, it forms catch bonds. They finish their study by exploring the hypothesis that accessory receptor-ligand interactions, specifically CD2-CD58 and LFA-1-ICAM, might promote antigen discrimination by shielding TCR-pMHC interactions from applied force. This analysis exploits functional data from a previous study (Pettmann et al., Elife 2021).

The results presented here are eye opening because prior work had suggested that many TCRs form catch bonds and therefore that the application of force enhances TCR ligand discrimination. The authors of this study come to the exact opposite conclusion. They also provide an interesting potential basis for the CD2 and LFA-1 as "force shielding" receptors. I found this paper to be insightful and thought provoking, and as such I feel that it represents an important contribution to a rapidly evolving field. I really have only one suggestion for improvement.

If the force shielding hypothesis is correct, one would predict that CD2-CD58 and/or LFA-1-ICAM interactions would affect ligand discrimination by slip bond receptors differently than ligand discrimination for catch bond receptors. The authors have both slip

bond (e.g. 1G4) and catch bond (e.g. OT-1) receptors in hand, and they are therefore in a position to test this prediction.

Referee #2:

Detailed comments on the ms. titled "Mechanical forces impair antigen discrimination by reducing differences in T cell receptor off-rates" by Pettmann et al. (for submission to the authors)

Pettmann et al. combine theoretical modelling with a laminar flow chamber (LFC) assay, molecular dynamics simulations as well as previously published T cell activation assays to assess the role of mechanical forces exerted on T cell antigen receptors (TCRs) bound to peptide-loaded MHC molecules (pMHCs) on T-cell antigen discrimination. Based on their experimental data, calculations and simulation results they conclude that low affinity TCR-pMHC interactions are, at least when compared to higher affinity TCR-pMHC interactions, less susceptible to disruption by mechanical forces. The authors then reason that antigen discrimination by T cells suffers from TCR-pMHC exerted forces - unlike what has previously been proposed - and benefits from cell-cell adhesion mediated for example by LFA-1 and CD2.
While I find the chosen approach stimulating and some of the data shown peculiar, I am, as I will state in more detail below, not yet convinced by the evidence provided. My reservation is primarily based on my concerns regarding (i) the choice of force ranges applied, (ii) the power and validity of the laminar flow chamber assay and (iii) the conclusions drawn from the resulting experiments.

(i) Choice of force range applied and force modalities
Previous studies which have focused on forces applied to individual TCRs have indicated values ranging between less than 2 pN to 15 pN. The authors may want to state more clearly why they have focused their analysis on forces ranging between 5 and > 100 pN. I consider this an important issue, because the chosen force range appears largely above the observed physiological range, and hence I question the relevance of the reported findings for a better understanding of T-cell recognition. Surely, bulk-force measurements (e.g. traction force microscopy) have invoked such a range, but why would the authors consider them as a guiding range for their stated single molecule force measurements?
Another aspect concerns the direction of applied forces. The authors mention and indicate in their graphs pulling forces, more or less in line with the TCR-pMHC length axis. As far as I can tell, the LFC-assay is clearly based on the exertion of shear-forces, which (a) may or may not come to register in synaptic setting and (b) may furthermore exert an influence on the overall TCR-pMHC stability that differs from that resulting from normal forces. In fact, the molecular dynamics simulations assume altogether normal forces. In my view this incongruency needs to be resolved.

(ii) Power and validity of the laminar flow chamber assay
There are a number of oddities I perceive when examining the survival-against-time plots in figure 2B. First of all, the plots do not appear to follow (at least in their current form) a single exponential function. I suggest that the authors include such a fit, or alternatively they render the data in form of a semi-exponential plot to give reference to single exponential decay. Another aspect that confuses me is why the survival value does not approach 0 for all plots but takes instead a different value for every force value applied. Moreover, there is no perceivable difference between the 67 pN and 91 pN force plot. Subtracting an off-set, which appears to differ from measurement to measurement (for reasons to be further explained) would change the results plotted in figure 2c with implications for the overall message.
Also, why did the authors not display and account for later decay behaviour since there are still a great number of surviving bonds after 5 seconds of recording. Is the decay bi-or multi-phasic by any chance? How often have these experiments been repeated, and would it be possible to indicate a confidence interval?
I could not find any answers to my questions. In case they have been provided somewhere in the ms. they should be placed front and centre to facilitate the reading process.

(iii) Validity of the conclusions drawn from the LFC-based experiments
I am hesitant to accept the validity of the numerical analysis of koff0 and Xβ as shown for example in figure 2c. First of all, I do not necessarily see single exponential decay in figure 2b (see above), a prerequisite for further analysis. Secondly, there appears to be an anomaly at 14 pN forces, which, if true, indicates catch-bond behaviour. What do the authors then make of this if they formulate for the entire function a slip-bond property? Would the combined data not suggest the existence of a catch-slip bond as put forward by Zhu and colleagues? If true, how could such behavior be described with Xβ being a constant? Would catch-slip bond behaviour not indicate that certain events, which guide the binding behaviour, are not accounted for by the rather simple model?
Furthermore, I have doubts regarding the sensitivity of the LFC-based assay which, according to the authors, truthfully reflects single bond behaviour, even for low affinity interactions. What exactly is the noise level in this assay resulting from (a) non-specific bead-glass interactions and (b) di- or multivalent interactions? I am not aware a single negative control (e.g. TCR-mismatched pMHC or pMHC-mismatched TCR) to serve as reference. I find it insufficient to refer to published literature for method validation and would feel more confident about the conclusions drawn if I had been presented with a negative control carried out in parallel with any experiment shown.
I also think it would be appropriate to show the survival over time plots for all experiments (including exponential fit) and to feature the camera recordings online. Creating a solid foundation with primary data-related evidence appears critically important,

especially if the study reports a finding that goes against intuition (e.g. (a) the catch bond behaviour of A6 + Tax 7Q at 110 pN with a KD of ~ 35 µM and a koff0 of 0.84s-1 or (b) the catch bond behaviour of OT-1 + OVA at 110 pN with a KD of 28 µM and a koff0 of 1.3s-1).
How reliable are the NYE 4D measurements? I would assume that given the reduced on-rate, witnessing the occurrence of a single bond may be rather challenging. The scenario appears even more dismal for lowest affinity ligands such as endogenous ligands.
Last but not least, more detailed screening of the force range between 0 and 10 pN would be highly informative (see above). But does the LFC-based assay actually support such measurements (maybe through the use of smaller beads)? Such analysis would refocus the study towards physiologically more relevant force ranges.

Referee #3:

Pettmann et al. used computational modeling and flow-chamber-based and solution-based binding assays to investigate how mechanical forces affect TCR antigen recognition. They unexpectedly found lower-affinity pMHCs were more resistant to mechanical force than higher-affinity interactions. And this lower-affinities pairs form weak slip or catch bonds, while the higher ones forms strong slip bonds. Based on these characterizations, they suggest force may impair TCR antigen discrimination and ahdesion receptors provide force-shielding role. The computational modeling part is beautiful, but the flow chamber assay has significant technical concerns. So, I feel these conclusions are not strong enough supported by their experiments due to technical concenerns and other comments listed below, their overall conclusion are over interpretated. I do not think this form is suitable for EMBO J.

Major concerns and questions:
1. In the abstract, the statement "...but how force impacts TCR/pMHC off-rates remains unclear" seem not very right, as there are multiple published papers from different groups have clearly demonstrated force can regulate TCR/pMHC dissociation using highly sensitive single-molcule assays (e.g. biomembrane force probe and optical tweezers from Lang, Ellis, Zhu, Garcia, and Chen groups); and in the introduction, authors kept repeating similar statements, which are not appropriate. In the introduction, they also mentioned "how force impact TCR pmhc interaction koff(m) and antigen discrimination is controversial". I highly suggest authors need to carefully revisit those papers and accordingly revise these statements.
2. In the 1st paragraph of introduction, author mentioned ref15 catch bond can be abolished purified form. I went back to look at this reference, I didn't find out related data to support this statement. Can author re-clarify this point?
3. And they further suggest "some catch bond ... may be secondary to TCR signaling rather than intrinsic to TCR/pmHC interaction". Several papers have clearly demonstrated from Ellis and Zhu groups showing that catch bond is clearly essential for triggering TCR signaling. And also the TCR/CD3 complex sitting on the membrane provides an critical biophysical regulation on TCR/pMHC interaction, compared to purified forms. Davis, Zhu, Ellis, Chen, and Garcia's groups have clearly shown these in-situ TCR/pMHC binding are more important than the "intrinsic binding" author claimed for TCR triggering and antigen discrimination. I not very clear why authors still kept focusing on "intrinsic ones". I suggest authors should at least study purified TCR/CD3 complex binding with pMHC if possible if they would like to characterize "intrinsic binding kinetics".
4. Technical concerns of flow chamber assay used in this work is a critical issue of this work. Authors used a camera with low temporal resolution (50Hz based on their methods) to capture the TCR/pMHC binding which are of fast kinetics. Based on data obtained by this slow capturing camera, their temporal resolution is much lower compared to those in the optical tweezer and biomembrane force probe assay (Zhu, Ellis, Lang groups).Such that, they missed many fast binding events, leading to significantly bias their final conclusions. I would suggest authors use faster cameras to redo their experiments with at least CD3 complex associated TCRs.
5. Another technical concern, I don't know why authors set a 1s cut off in the analysis of their tethering lifetimes from flow-chamber assay. This cut off would also impact the final average lifetimes or koff(m) very much such that the force-dependent TCR/pMHC bond lifetimes would change very much. I do not think this cut off is appropriate.
6. Use flow chamber assay to study single-molecule binding is challenging, although author claimed they did at the single-molecule level. I am not very clear how they achieved this single-molecule level. I strongly suggest authors to perform single-molecule binding assay either with optical tweezer or biomembrane force probe to confirm their results (like Zhu and McEver group did before on selectin/ligand binding), otherwise current results are very ambiguous.
7. The lower bound of flow-chamber assay is hard to reach below 10pN range like bioforce assay, I feel author may miss this critical regime, so they obtained very different data from optical tweezer and biomembrane force assay. Actually in their Fig.S6B,Fig.2C,D,G, in the lower bound, their data kind of showing very little catch trends. Again, I suggest they should also repeat their work with single-molecule binding assay.
8. Other concerns of their MD simulation. As they used coarse grained MD simulation instead of all-atom simulations like Garcia and Chen group did before, such that there are some intra-molecular conformational changes are not able to be observed. This might affect the rupture force and Fmax value from the simulation. So, I suggest authors need to repeat these simulations with all-atom MD. How to define and calculate Fmax, as this is very important for calculating Xb and koff.
9. Regarding the data on the OT-1/OVA interaction depicted in Fig.S6, what is the authors' criteria for the definition of low-affinity TCRs? Davis and Zhu groups, two nature papers have clearly shown the in-situ binding affinity is more appropriate than in-solution ones for TCR.
10. In the discussion part, many statements are over-interpreted given their data not convinced enough. I strongly suggest authors to revise the discussion accordingly.

Minor comments:

1. On page 8, line 10,the rate of each step of kinetic proofreading model in brackets is omitted.
2. Page 8, paragraph 2, line 4, the symbol of rate seems missing.
3. How was the global threshold λ selected in this study?

Response to Reviewers

Referee #1

"Mechanical forces impair antigen discrimination by reducing differences in T cell receptor off-rates", by Pettmann et al., combines computational modeling with cell free biophysical measurements to investigate the force sensitivity of several TCR-pMHC interactions. The authors find, unexpectedly, that most of the interactions exhibit slip bond behavior and that higher affinity interactions are especially sensitive to the application of force. These observations run counter to prior results from the biomembrane force system. The authors then apply molecular dynamics simulations to validate the slip bond behavior and force sensitivity of several TCR-pMHC pairs of interest. In an interesting aside, they demonstrate that the OT-1 TCR, long thought to recognize its cognate pMHC with high affinity, is actually a low affinity binder with a very fast off-rate, and that similar to some other low affinity binders, it forms catch bonds. They finish their study by exploring the hypothesis that accessory receptor-ligand interactions, specifically CD2-CD58 and LFA-1-ICAM, might promote antigen discrimination by shielding TCR-pMHC interactions from applied force. This analysis exploits functional data from a previous study (Pettmann et al., Elife 2021).

The results presented here are eye opening because prior work had suggested that many TCRs form catch bonds and therefore that the application of force enhances TCR ligand discrimination. The authors of this study come to the exact opposite conclusion. They also provide an interesting potential basis for the CD2 and LFA-1 as "force shielding" receptors. I found this paper to be insightful and thought provoking, and as such I feel that it represents an important contribution to a rapidly evolving field. I really have only one suggestion for improvement.

We thank the reviewer for taking the time to read our manuscript and for suggesting the functional experiment below.

If the force shielding hypothesis is correct, one would predict that CD2-CD58 and/or LFA-1-ICAM interactions would affect ligand discrimination by slip bond receptors differently than ligand discrimination for catch bond receptors. The authors have both slip bond (e.g. 1G4) and catch bond (e.g. OT-1) receptors in hand, and they are therefore in a position to test this prediction.

When contacting a collaborator to discuss experiments to use the OT-I TCR, they pointed out that these experiments have recently been published for the first time last week (Li et al (2022) Science Immunology; https://www.science.org/doi/10.1126/sciimmunol.abn6373). They stimulated OT-I CD8+ T cells from WT or CD2 KO mice finding that CD2 KO had “a more pronounced defect [was noted] with the low-affinity agonistic peptide G4” compared to the high-affinity agonistic OVA peptide N4 (Figure 1D and E of their work). In contrast to the data with the 1G4 TCR where CD2 has a larger impact on the higher-affinity antigen, this work shows that CD2 has a larger impact on the lower-affinity antigen.

We note that CD2 has both an adhesion function and a signalling function (as discussed in our manuscript). The signalling function can presumably work even when CD2 is engaged in cis with CD48/CD58 on the same T cell and does not require close co-localisation with the TCR/pMHC interaction and it will have a positive role independent of the catch/slip nature of

the interaction. Our interpretation of Li et al is that they are providing evidence that the signalling function of CD2 can take place when ligated in cis and has a positive role for both high and low affinity ligands (i.e. for both catch and slip bonds).

We have revised the discussion to highlighting this result with OT-I.

Referee #2

Detailed comments on the ms. titled "Mechanical forces impair antigen discrimination by reducing differences in T cell receptor off-rates" by Pettmann et al. (for submission to the authors)

Pettmann et al. combine theoretical modelling with a laminar flow chamber (LFC) assay, molecular dynamics simulations as well as previously published T cell activation assays to assess the role of mechanical forces exerted on T cell antigen receptors (TCRs) bound to peptide-loaded MHC molecules (pMHCs) on T-cell antigen discrimination. Based on their experimental data, calculations and simulation results they conclude that low affinity TCR-pMHC interactions are, at least when compared to higher affinity TCR-pMHC interactions, less susceptible to disruption by mechanical forces. The authors then reason that antigen discrimination by T cells suffers from TCR-pMHC exerted forces - unlike what has previously been proposed - and benefits from cell-cell adhesion mediated for example by LFA-1 and CD2.

While I find the chosen approach stimulating and some of the data shown peculiar, I am, as I will state in more detail below, not yet convinced by the evidence provided. My reservation is primarily based on my concerns regarding (i) the choice of force ranges applied, (ii) the power and validity of the laminar flow chamber assay and (iii) the conclusions drawn from the resulting experiments.

We thank the reviewer for taking the time to read our manuscript and for providing constructive comments to improve it.

(i) Choice of force range applied and force modalities
Previous studies which have focused on forces applied to individual TCRs have indicated values ranging between less than 2 pN to 15 pN. The authors may want to state more clearly why they have focused their analysis on forces ranging between 5 and > 100 pN. I consider this an important issue, because the chosen force range appears largely above the observed physiological range, and hence I question the relevance of the reported findings for a better understanding of T-cell recognition. Surely, bulk-force measurements (e.g. traction force microscopy) have invoked such a range, but why would the authors consider them as a guiding range for their stated single molecule force measurements?

To our knowledge, there are no direct measurements of the force experienced by TCR/pMHC at T cell/APC interfaces making it difficult to identify a physiological range. The study that most closely measured applied forces on the TCR/pMHC interaction is by Gerhard Schtuz (Göhring et al (2021) Nature Communications) but they were unable to detect any forces above 2 pN (limit of their detector) on the TCR/pMHC when presenting both pMHC and ICAM-1 on mobile bilayers. In this condition, T cells were robustly activated allowing them to conclude that an applied force is not necessary for TCR triggering or T cell activation (consistent with our proposed model). On the other hand, Marco Fritzsche (Colin-York et al (2019) Cell Reports) inferred forces of 150 pN using traction force microscopy when presenting immobile pMHC in isolation. Their argument is that the traction forces were wholly mediated by TCR/pMHC interactions because no other ligands were presented. A

limitation is that this 150 pN force is an upper limit because it may have been shared across multiple TCR/pMHC interaction and therefore, the actual forces on individual TCR/pMHC interaction may be lower.

We agree with the reviewer that the community using the biomembrane force probe with live T cells has focused on artificially applying external forces in the range of 2 to 30 pN. However, as explained above, much larger forces can be generated and force is not required to activate T cells.

We have revised the introduction to highlight the force ranges that have been measured.

Another aspect concerns the direction of applied forces. The authors mention and indicate in their graphs pulling forces, more or less in line with the TCR-pMHC length axis. As far as I can tell, the LFC-assay is clearly based on the exertion of shear-forces, which (a) may or may not come to register in synaptic setting and (b) may furthermore exert an influence on the overall TCR-pMHC stability that differs from that resulting from normal forces. In fact, the molecular dynamics simulations assume altogether normal forces. In my view this incongruency needs to be resolved.

While a shear force is applied on the microsphere in the laminar flow chamber, the force exerted on the TCR-pMHC bond is of normal orientation because of the flexibility in the anchor of both molecules (see Figure below).

The pMHC is biotinylated on a BirA sequence located 14 amino acids downstream of a linker on the C-terminal of the HLA-A2 heavy-chain sequence. The TCR is linked to the microsphere surface through a proximal His-Tag bound through an amino-acids linker to an anti-His antibody possessing inherent Fab-Fc joint flexibility, itself covalently bound to the microsphere surface. Collectively, this allows for rotational movement of both molecules on their anchored surface so that the flow velocity on the microsphere translates into a force along the TCR/pMHC interaction axes (i.e. bond formation oriented along the force axis).

We have introduced text in the results with a reference to Extended View Figure S2 to explain how shear forces are resolved into normal forces including citations to previous work.

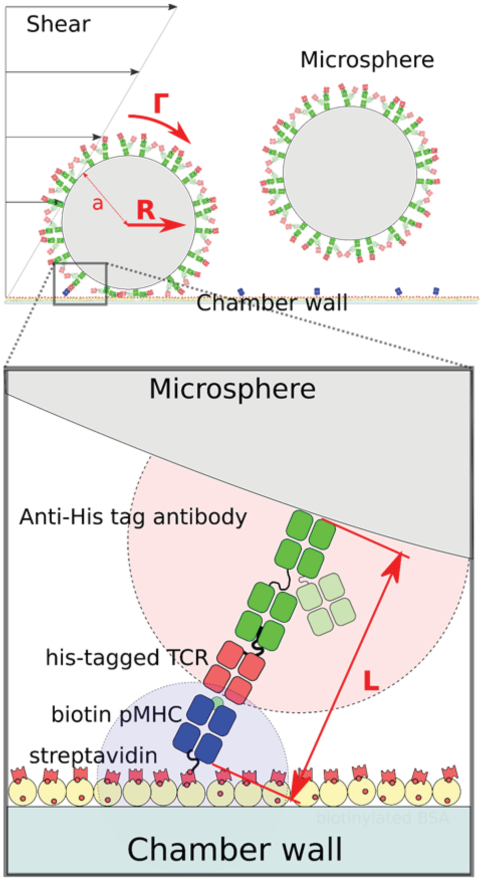

Details on how shear force is converted to normal force on the TCR/pMHC:
Biotinylated pMHC and antibody-bound TCR have rotational freedom allowing their binding site to diffuse in volumes represented by potions of disc in light blue for the pMHC and light red for TCR. Near to the surface of the chamber, the flow velocity is given by a first order approximation: v(z) = Gz Where z is the distance to the surface in nm and G is a constant called shear rate in s^{-1}. The shear stress Ts (in N/m2 or pascals) applied corresponds to the force applied by the flow per surface unit and is calculated by the product between G and the viscosity of the medium (μ) in Pa/s: □□ = μ□. Taking into account the dimensions of the LFC section ı x H, shear rate can be determined for a given flow Q using the formula: □ = □□ □□□. When the microsphere makes a link with the surface the flow creates a hydrodynamic force (R) given by the equation: R = 32μa 2G = =1.7005×6πμa 2G where a is the radius of the microsphere. The microsphere is also subjected to a torque force (Γ): Γ = 0.9440 × 4μa 3G In addition, a lever effect increases the force applied to the interaction, so the total force applied (F) is given by the equation: □ = □ + (□ □) √□/□□ where L is the length of the formed bond, i.e., the length of the link. (A. Pierres, A.-M. Benoliel, and P. Bongrand, “Measuring the Lifetime of Bonds Made between Surface-linked Molecules,” J. Biol. Chem., vol. 270, no. 44, pp. 26586–26592, Nov. 1995).

(ii) Power and validity of the laminar flow chamber assay
There are a number of oddities I perceive when examining the survival-against-time plots in figure 2B. First of all, the plots do not appear to follow (at least in their current form) a single

exponential function. I suggest that the authors include such a fit, or alternatively they render the data in form of a semi-exponential plot to give reference to single exponential decay.

The reviewer is correct that the survival curves do not follow a single exponential. This was discussed in our previous paper for TCR/pMHC interactions (Robert et al (2012) Biophysical Journal, PMID: 22339861), including different analysis methods (including exponential fits). Note that a single exponential is not always observed when using the biomembrane force probe (e.g. Fig S2A of Liu et al (2014) Cell, PMID: 30420628).

We have repeated the entire analysis by fitting a single exponential (but with a baseline plateau parameter) and find that our conclusions are unchanged. This is discussed in the results and shown in the Appendix.

Another aspect that confuses me is why the survival value does not approach 0 for all plots but takes instead a different value for every force value applied.

This is related to the above point on why the survival curves do not follow a single exponential. We first observed this in our 2012 publication for TCR/pMHC interaction (Robert et al (2012) Biophysical Journal, PMID: 22339861) and the field refers to this as “history dependence of bond dissociation”. It is discussed in our previous work and references therein.

We have now revised the results to include a reference to our previous paper.

Moreover, there is no perceivable difference between the 67 pN and 91 pN force plot. Subtracting an off-set, which appears to differ from measurement to measurement (for reasons to be further explained) would change the results plotted in figure 2c with implications for the overall message.

We have analysed the data using several methods, including an exponential with a fitted baseline plateau parameter (i.e. off-set) that can be different for different datasets and find that our conclusions are unchanged.

Also, why did the authors not display and account for later decay behaviour since there are still a great number of surviving bonds after 5 seconds of recording. Is the decay bi-or multi-phasic by any chance?

A limitation of the LFC is that, by continually flowing microspheres over the surface, two microspheres can come into proximity and in this situation, we cannot tell them apart. The chances that this takes place after a bead arrest increases with time and as a result, on longer timescales, we can only produce a lower bound for arrest durations. By focusing on the earlier part of the survival curve, we are able to avoid this complication. We note our analysis does reproduce similar values to those measured by SPR (at low forces or extrapolated) validating our method and that we have used the same time window in our previous work (Robert et al (2012) Biophysical Journal, PMID: 22339861).

We have revised the results section to explain this limitation of the LFC and why we have focused on the first 5 seconds.

How often have these experiments been repeated, and would it be possible to indicate a confidence interval?

In the caption of Figure 2, we state “The number of independent experiments performed on different days that were combined to produce the estimated off-rates are: 7 (1G4/9V), 8 (1G4/6V), 8 (1G4/4E5E8E), 10 (1G4/3Y), 6 (1G4/4D), 9 (A6/Tax WT), 11 (A6/5F), 9 (A6/7Q), 8 (A6/7R), 8 (A6/5H).”

We have revised the results section to explicitly state the number of experiments performed overall in our study.

I could not find any answers to my questions. In case they have been provided somewhere in the ms. they should be placed front and centre to facilitate the reading process.

(iii) Validity of the conclusions drawn from the LFC-based experiments
I am hesitant to accept the validity of the numerical analysis of koff0 and Xβ as shown for example in figure 2c. First of all, I do not necessarily see single exponential decay in figure 2b (see above), a prerequisite for further analysis.

We agree that a single exponential decay cannot explain the survival curves shown in Figure 2B on the timescale of 5 seconds. However, it is not clear to us why a single exponential decay in the data is required for further analysis. The analysis we presented does not depend on the precise distribution of the survival distribution and moreover, we can fit a single exponential decay provided it includes a baseline plateau and this analysis produces the same conclusions. We note that multi-phasic survival curves are not unique to our LFC assay but have also been observed by independent groups using different assays (e.g. Fig S2A of Liu et al (2014) Cell, PMID: 30420628).

We have revised the manuscript to include analysis using an exponential fit and find that our conclusions are unchanged.

Secondly, there appears to be an anomaly at 14 pN forces, which, if true, indicates catch-bond behaviour. What do the authors then make of this if they formulate for the entire function a slip-bond property? Would the combined data not suggest the existence of a catch-slip bond as put forward by Zhu and colleagues? If true, how could such behavior be described with Xβ being a constant? Would catch-slip bond behaviour not indicate that certain events, which guide the binding behaviour, are not accounted for by the rather simple model?

We agree that in 3 of the 10 interactions in Figure 2 (of the submitted manuscript), the 14 pN measurements produces an off-rate that is below the value at 5 pN. However, the effect size is very small (and much smaller than that reported by Zhu and colleagues for catch bonds) and within the general variation of the data.

In response to other comments, we have now also included an analysis where we subtract non-specific binding. Our overall conclusions do not change but we noted that the small decrease in the off-rate at 14 pN for the 3 interactions is reduced (1 of 3) or abolished (2 of 3). We have moved the original analysis from the submitted paper into the Appendix and display the corrected data in the main text.

Furthermore, I have doubts regarding the sensitivity of the LFC-based assay which, according to the authors, truthfully reflects single bond behaviour, even for low affinity interactions. What exactly is the noise level in this assay resulting from (a) non-specific bead-glass interactions and (b) di- or multivalent interactions? I am not aware a single negative control (e.g. TCR-mismatched pMHC or pMHC-mismatched TCR) to serve as reference. I find it insufficient to refer to published literature for method validation and would feel more confident about the conclusions drawn if I had been presented with a negative control carried out in parallel with any experiment shown.

We referred to our previous published work for these controls to avoid showing them again but we understand the reviewer's concern and have now included all of these controls in the manuscript.

(a) The noise level from non-specific binding can range from 0 to about 50% with the larger amount of non-specific binding appearing at very low and very high velocities. This is a result of non-specific interactions between microspheres and the flow chamber surface whereby beads can appear to arrest non-specifically and this places a lower bound on the force we can apply in the assay to 5 pN (see also response to the last comment below). At very high velocities, the encounter duration between TCR and pMHC is short reducing the probability of binding.

    We have included a discussion of this when we first describe the LFC and have included supplementary figures showing this over flow velocity.

(b) We perform all experiments in the single-bond regime by immobilising very low levels of pMHC on the surface.
    (i) We can directly assess this by showing that when you lower the pMHC density on the surface, the survival curve is unchanged (Fig S1). On the other hand, when multivalent interactions are present, the survival curves are sensitive to pMHC densities and this is readily observed at higher densities. This is a standard method for determining that an interaction is taking place based on single bonds and it is also used by the biomembrane force probe community. For example, in Liu B, Chen W, Evavold B, Zhu C (2014) Cell, Cheng Zhu and colleagues explain "To ensure that most binding events were mediated by single bonds, adhesion frequencies (number of adhesions divided by number of contacts) were kept low (<20%) by adjusting the pMHC density on the probe bead (Chesla et al., 1998) (Figures 1F and S1A)."
    (ii) In addition, the laminar flow chamber offers another direct assessment of observation of single bonds by measuring the number of binding events relative to the distance travelled by microspheres (itself determining the number of ligand-receptor encounters.). This value is named "linear binding

density” in the text. If we observe single binding events, the linear binding densities are expected to increase linearly with the amount of deposited ligands. By contrast, in multiple bonds regimen, linear binding density will increase as a power law (with power <1) of the amount of deposited ligand. We find a linear increase in the density regime that we use to make our measurements (Fig S1). The combination of both controls (unchanging survival and binding densities proportional to ligand deposited) were explicitly described in the first publications using laminar flow chamber as a single bond measuring method (Measuring the lifetime of bonds made between surface-linked molecules.Pierres A, Benoliel AM, Bongrand P. J Biol Chem. 1995 Nov 3;270(44):26586-92. doi: 10.1074/jbc.270.44.26586.) This control is also included (Fig S1).

(iii) We also show that the predicted zero-force off-rate from the LFC is similar to the value obtained using SPR, again confirming that we are in the single-bond regime. This was also reported in our previous work on TCR/pMHC (see Robert, Aleksic, Dushek et al (2012) Biophysical Journal, PMID: 22833746).

(iv) Lastly, we can reproduce the catch bonds observed for the OT-I TCR.

We agree that measurements for lower-affinity interactions are more difficult because the short arrest duration of the beads cannot be distinguished from background. A similar situation takes place at large forces for all ligands and again, data at these high forces cannot be reliably included.

We now include control data for surfaces without pMHC or when the pMHC is mismatched to the TCR and the fold-change in BLD for specific interactions under various flow rates (Figure EV4,EV5). We have also revised the entire analysis to subtract this non-specific binding and can confirm that all of our conclusions are unchanged. We include the original data submitted with the manuscript in the Appendix. The result section has been updated.

I also think it would be appropriate to show the survival over time plots for all experiments (including exponential fit) and to feature the camera recordings online. Creating a solid foundation with primary data-related evidence appears critically important, especially if the study reports a finding that goes against intuition (e.g. (a) the catch bond behaviour of A6 + Tax 7Q at 110 pN with a KD of ~ 35 µM and a koff0 of 0.84s-1 or (b) the catch bond behaviour of OT-1 + OVA at 110 pN with a KD of 28 µM and a koff0 of 1.3s-1).

We have included the survival curves over time for all experiments showing survival at 1 s, 2 s, and exponential fit. All the bond lifetime data is present in survival curves.

We have included a supplementary video showing an example of the camera recording. We note that it is difficult to obtain intuition by looking at the videos. Like the biomembrane force probe, it is a niche technique and therefore, the data is not in a structured format that is often re-used. We have not been contacted previously by anyone asking for the data and note that the full dataset is multiple terabytes. We suggest that we continue to make the data available upon request and can provide the reviewer with any data they require.

How reliable are the NYE 4D measurements? I would assume that given the reduced on-rate, witnessing the occurrence of a single bond may be rather challenging. The scenario appears even more dismal for lowest affinity ligands such as endogenous ligands.

We agree that it is more difficult to collect data for lower-affinity ligands. The multiple controls we have in place determine whether an interaction (specific TCR/pMHC at a specific flow velocity) can be included in the analysis (EV Figure S5 shows data points in red that were not included for example). However, the data for NYE 4D passed our quality control checks and we include all survival curves, including for NYE 4D, in the Appendix Figures.

Last but not least, more detailed screening of the force range between 0 and 10 pN would be highly informative (see above). But does the LFC-based assay actually support such measurements (maybe through the use of smaller beads)? Such analysis would refocus the study towards physiologically more relevant force ranges.

Currently, the sensitivity of the assay does not allow us to reduce forces below 5 pN. Below this value, the signal-to-noise ratio reduces very quickly as many bead arrests non-specifically (see comment above regarding controls). Reducing the microsphere diameter is not a simple solution because microsphere weight is critical to set the average distance between the microsphere surface and the flow chamber surface; currently, we use iron oxide doped polystyrene microspheres (Dynabeads) because their higher density allows for small yet heavy beads. We have not found a way to produce data at lower forces at the moment. We refer to our response to the very first comment about the physiological force range.

We have revised the first results section to highlight the force range that can be used in the LFC assay.

Referee #3

Pettmann et al. used computational modeling and flow-chamber-based and solution-based binding assays to investigate how mechanical forces affect TCR antigen recognition. They unexpectedly found lower-affinity pMHCs were more resistant to mechanical force than higher-affinity interactions. And this lower-affinities pairs form weak slip or catch bonds, while the higher ones forms strong slip bonds. Based on these characterizations, they suggest force may impair TCR antigen discrimination and ahdesion receptors provide force-shielding role. The computational modeling part is beautiful, but the flow chamber assay has significant technical concerns. So, I feel these conclusions are not strong enough supported by their experiments due to technical conceneerns and other comments listed below, their overall conclusion are over interpretated. I do not think this form is suitable for EMBO J.

Major concerns and questions:

1. In the abstract, the statement "...but how force impacts TCR/pMHC off-rates remains unclear" seem not very right, as there are multiple published papers from different groups have clearly demonstrated force can regulate TCR/pMHC dissociation using highly sensitive single-molcule assays (e.g. biomembrane force probe and optical tweezers from Lang, Ellis, Zhu, Garcia, and Chen groups); and in the introduction, authors kept repeating similar statements, which are not appropriate. In the introduction, they also mentioned "how force impact TCR pmhc interaction koff(m) and antigen discrimination is controversial". I highly suggest authors need to carefully revisit those papers and accordingly revise these statements.

We apologise for the confusion.

We argue that those statements are correct when applied to TCR/pMHC interactions because the majority of the published data cited by the reviewer refer to assays with live T cells. Therefore, how force impacts **T cell** interaction with pMHC has been measured but very little work has been performed in a cell-free system to study how force impacts the **T cell receptor** interaction with pMHC. We argue in the manuscript that this is a key difference between published work and this work.

We have revised the introduction to more explicitly convey the distinction between how force impacts the TCR/pMHC off-rates (our work) and how force impacts adhesion between T cells and pMHC (what is measured in the majority of studies).

2. In the 1st paragraph of introduction, author mentioned ref15 catch bond can be abolished purified form. I went back to look at this reference, I didn't find out related data to support this statement. Can author re-clarify this point?

We apologise for this. The correct reference is PMID: 31315981 and this is now revised.

3. And they further suggest "some catch bond ... may be secondary to TCR signaling rather than intrinsic to TCR/pmHC interaction". Several papers have clearly demonstrated from Ellis and Zhu groups showing that catch bond is clearly essential for triggering TCR

signaling. And also the TCR/CD3 complex sitting on the membrane provides an critical biophysical regulation on TCR/pMHC interaction, compared to purified forms. Davis, Zhu, Ellis, Chen, and Garcia's groups have clearly shown these in-situ TCR/pMHC binding are more important than the "intrinsic binding" author claimed for TCR triggering and antigen discrimination. I not very clear why authors still kept focusing on "intrinsic ones". I suggest authors should at least study purified TCR/CD3 complex binding with pMHC if possible if they would like to characterize "intrinsic binding kinetics".

We apologise for the confusion.

We agree that when using live T cells, a catch bond is always observed for ligands that activate T cells. The community has interpreted this to mean that catch bonds are essential for triggering. We are trying to contribute to the discussion by pointing out that another interpretation is that these ligands induce signalling that increases the ability of T cells to bind the ligand, and hence observe an apparent/effective catch bond. We are trying to contribute to the discussion to highlight this point and that cell-free experiments are needed to parse out whether the catch bond is intrinsic to the TCR/pMHC (i.e. does not require any active process) or whether it requires TCR signallling.

Indeed, the studies quoted by the reviewer have shown that inhibiting T cell processes can impact the TCR/pMHC interactions (e.g. cytoskeleton disruption impacts 2D kinetics, see Huppa et al (2010) Nature, Huang et al (2010) Nature). Moreover, when the biomembrane force probe is used to compare the OT-I/OVA interaction using T cells vs a cell-free system, there is a clear and dramatic reduction in the magnitude of the catch bond (Liu et al (2015) European Journal of Immunology).

The response to comment 1 above and the revised introduction should clarify the difference between our study (cell-free, intrinsic TCR/pMHC interaction) and published work (using live T cells, catch bond is between a T cell and pMHC, i.e. not intrinsic to the TCR/pMHC interaction).

Binding studies with purified TCR/CD3 complex have yet to be performed by any laboratory as it is presently difficult to maintain a high concentration of monomeric TCR-CD3 complexes required for these measurements. We note that catch bonds do not require the full TCR/CD3 complex, which is illustrated by the catch bond formed by OT-I TCR (present work, Cheng Zhu’s work), N15 TCR (Lang’s work, PMID: 25605925), and the recent work by Garcia where he argues that the CD3 complex is not important for catch bond formation because they can convert slip bonds to catch bonds by modest mutations to the CDR2 loops, albeit with the BFP assay using live T cells (PMID: 35389803).

4. Technical concerns of flow chamber assay used in this work is a critical issue of this work. Authors used a camera with low temporal resolution (50Hz based on their methods) to capture the TCR/pMHC binding which are of fast kinetics. Based on data obtained by this slow capturing camera, their temporal resolution is much lower compared to those in the optical tweezer and biomembrane force probe assay (Zhu, Ellis, Lang groups).Such that, they missed many fast binding events, leading to significantly bias their final conclusions. I

would suggest authors use faster cameras to redo their experiments with at least CD3 complex associated TCRs.

The typical TCR/pMHC binding kinetics are on the order of 0.1-10 /s (i.e. 0.1-10 Hz) and therefore, our 50 Hz camera is appropriate for detecting these interactions. We are not aware of any reported TCR/pMHC interactions with koff faster than 50 Hz. The groups of Zhu, Ellis, and Lang report off-rates (or lifetimes) within 50 Hz.

See previous response for comment on using TCR-CD3 to measure affinity/kinetics/force.

5. Another technical concern, I don't know why authors set a 1s cut off in the analysis of their tethering lifetimes from flow-chamber assay. This cut off would also impact the final average lifetimes or koff(m) very much such that the force-dependent TCR/pMHC bond lifetimes would change very much. I do not think this cut off is appropriate.

We have revised the results to include multiple analyses methods, including calculations based on 1s, 2s and an exponential fit. We find that our conclusions are unchanged. This was also requested by Reviewer 2.

6. Use flow chamber assay to study single-molecule binding is challenging, although author claimed they did at the single-molecule level. I am not very clear how they achieved this single-molecule level. I strongly suggest authors to perform single-molecule binding assay either with optical tweezer or biomembrane force probe to confirm their results (like Zhu and McEver group did before on selectin/ligand binding), otherwise current results are very ambiguous.

Flow chamber is a pioneering method in single bond measurement, and it was used to measure the first observed catch bond (W. E. Thomas *et al,* Cell, vol. 109, no. 7, pp. 913–923, Jun. 2002.). It was also used to measure bond lifetime of selectin-ligand bond (Masson-Gadais *et al.*,. *J Cell Sci* **112 (Pt 14)**, 2335–2345 (1999).), streptavidin-biotin bond (A. Pierres *et al.*, *Biophys J* **82**, 3214–3223 (2002).) integrin-ligand bond(10), antigen antibody bonds (P. Robert *et al.*, *Biophys J* **96**, 4642–4650 (2009); V. L. Schiavo *et al.*, *PLoS One* **7**, e44070 (2012)., P. Robert *et al.*, *Biophys J* **100**, 2642–2651 (2011)), CD2-CD48 bonds(A. Pierres *et al.*,*Proc. Natl. Acad. Sci. U. S. A.* **93**, 15114–15118 (1996)) and more recently TCR-pMHC bonds (P. Robert, *et al.*, *Biophys J* **102**, 248–257 (2012); L. Limozin, *et al.*, *Proc. Natl. Acad. Sci. U. S. A.* **116**, 16943–16948 (2019)).

We have several controls to ensure that we are in the single-molecule regime (see also the answer to Reviewer 2 on the same topic). We perform all experiments in the single-bond regime by immobilising very low levels of pMHC on the surface.

(i) We can directly assess this by showing that when you lower the pMHC density on the surface, the survival curve is unchanged (Fig S1). On the other hand, when multivalent interactions are present, the survival curves are sensitive to pMHC densities and this is readily observed at higher densities. This is a standard method for determining that an interaction is taking place based on single bonds and it is also used by the bioforce probe community

For example, in Liu B, Chen W, Evavold B, Zhu C (2014) Cell, Cheng Zhu and colleagues explain “To ensure that most binding events were mediated by single bonds, adhesion frequencies (number of adhesions divided by number of contacts) were kept low (<20%) by adjusting the pMHC density on the probe bead (Chesla et al., 1998) (Figures 1F and S1A).”

(ii) In addition, the laminar flow chamber offers another direct assessment of observation of single bonds by measuring the number of binding events relative to the distance travelled by microspheres (itself determining the number of ligand-receptor encounters.). This value is named “linear binding density” in the text. If we observe single binding events, the linear binding densities are expected to increase linearly with the amount of deposited ligands. By contrast, in multiple bonds regimen, linear binding density will increase as a power law (with power <1) of the amount of deposited ligand. We find a linear increase in the density regime that we use to make our measurements (Fig S1). The combination of both controls (unchanging survival and binding densities proportional to ligand deposited) were explicitly described in the first publications using laminar flow chamber as a single bond measuring method (Measuring the lifetime of bonds made between surface-linked molecules.Pierres A, Benoliel AM, Bongrand P. J Biol Chem. 1995 Nov 3;270(44):26586-92. doi: 10.1074/jbc.270.44.26586.) This control is also included (Fig S1).

(iii) We also show that the predicted zero-force off-rate from the LFC is similar to the value obtained using SPR, again confirming that we are in the single-bond regime. This was also reported in our previous work on TCR/pMHC (see Robert, Aleksic, Dushek et al (2012) Biophysical Journal, PMID: 22833746).

(iv) Lastly, we can reproduce the catch bonds observed for the OT-I TCR.

We have expanded the discussion of controls in the first results section.

7. The lower bound of flow-chamber assay is hard to reach below 10pN range like bioforce assay, I feel author may miss this critical regime, so they obtained very different data from optical tweezer and biomembrane force assay. Actually in their Fig.S6B,Fig.2C,D,G, in the lower bound, their data kind of showing very little catch trends. Again, I suggest they should also repeat their work with single-molecule binding assay.

As explained above, all the experiments have been performed in the single molecule regime.

We agree that in 3 of the 10 interactions in Figure 2 (of the submitted manuscript), the 14 pN measurements produces an off-rate that is below the value at 5 pN. However, the effect size is very small (and much smaller than that reported by Zhu and colleagues for catch bonds) and within the general variation of the data.

In response to other comments, we have now also included an analysis where we subtract non-specific binding. Our overall conclusions do not change but we now observe that the small decrease in off-rate at 14 pN for the 3 interactions is reduced (1 of 3) or abolished (2 of 3). We have moved the original analysis from the submitted paper into the Appendix and display the corrected data in the main text.

8. Other concerns of their MD simulation. As they used coarse grained MD simulation instead of all-atom simulations like Garcia and Chen group did before, such that there are some intra-molecular conformational changes are not able to be observed. This might affect the rupture force and Fmax value from the simulation. So, I suggest authors need to repeat these simulations with all-atom MD. How to define and calculate Fmax, as this is very important for calculating Xb and koff.

We considered using all atom simulations at the outset but unfortunately, it is computationally not possible. To understand this, consider that 400 trajectories (independent simulations) were required to produce Fig 3E. In standard all atom simulations, very few trajectories are performed and for example, all of the conclusions by the Garcia group (Sibener et al (2018) Cell) relied on a single trajectory (i.e. one MD simulations was performed for each applied force direction, which they show in the main text and do not show/reference any repeats or statistics). This is not uncommon for all atom MD simulations because of their computational cost.

The second point we considered is that all-atom simulations typically simulate hundreds of nanoseconds but the TCR/pMHC lifetime is on the order of 0.1-10 seconds even under force. To obtain unbinding on the nanosecond timescales, extremely large pulling velocities are typically used. For example, Wu et al used pulling speeds that are 200-2000 larger and Sibener et al used a pulling speed that is 1000-10,000 larger than the one used in the present work. Lastly, to obtain the force sensitivity from simulations, we need to vary the pulling speed over an order magnitude and at the lower end, it is expected that the simulations would take even longer.

The key advance of our structure-based coarse-grained simulations is that we were able to compare the same output from our experimental data and the simulations (negative correlation between x_beta and koff). In the Garcia (Sibener et al (2018) Cell) and Chen (Wu et al (2019) Molecular Cell) publications they argued for a catch bond in their simulations not by measuring off-rates but by examining hydrogen bonds and making inferences. However, the overall bond lifetime depends on other factors.

While we agree that our structure-based simulations coarse-grain the structure, we would argue that the key feature we report (negative correlation between x_beta and koff) are robust to the coarse-graining and have been preserved. Had we not observed this correlation, we could not rule out that the coarse-graining process may have removed them.

We have revised the discussion to include a paragraph comparing all-atom and coarse-grained simulations explaining why we were unable to use all-atom at present and suggest that this will be important when it is computationally feasible.

9. Regarding the data on the OT-1/OVA interaction depicted in Fig.S6, what is the authors' criteria for the definition of low-affinity TCRs? Davis and Zhu groups, two nature papers have clearly shown the in-situ binding affinity is more appropriate than in-solution ones for TCR.

We examined published data on TCR/pMHC interactions for MHC-I and found that the OT-I/OVA interaction has the fastest 3D koff that has ever been reported: “Our analysis shows that the OT-I/OVA interaction, which forms a catch bond, has an unusually fast off-rate for an agonist TCR/pMHC interaction (35), 13-17-fold faster than the 1G4/9V interaction (1).”

We are not clear what the reviewer means by ‘in-situ binding affinity is more appropriate’. The in-situ binding is assessed with live T cells and, because active T cell signalling can feedback to promote TCR/pMHC binding, these measurements are not intrinsic to the TCR/pMHC protein binding interface. For example, inhibiting the cytoskeleton reduced the 2D affinity observed in the Cheng Zhu (Fig S10 in https://pubmed.ncbi.nlm.nih.gov/20357766/), Mark Davis (Fig 2 in https://pubmed.ncbi.nlm.nih.gov/20164930/), and Jay Groves (Fig 5 in https://www.pnas.org/doi/10.1073/pnas.1613140114) publications.

Again, we apologise for the confusion and have revised the introduction to emphasise the concept of an affinity when using purified proteins compared to an “effective or apparent” affinity when using live T cells where active processes can contribute to the measurement.

10. In the discussion part, many statements are over-interpreted given their data not convinced enough. I strongly suggest authors to revise the discussion accordingly.

We have revised parts of the discussion. We note that without specific comments, it is difficult to know precisely the issue(s) the reviewer has concern over.

Minor comments:

1. On page 8, line 10,the rate of each step of kinetic proofreading model in brackets is omitted.

    Fixed.

2. Page 8, paragraph 2, line 4, the symbol of rate seems missing.

    This appears to be the same comment as the one above.

3. How was the global threshold λ selected in this study?

    This was a fitting parameter (it was not selected a priori).

Dear Omer,

Thank you for submitting your revised manuscript to The EMBO Journal. Your study has now been seen by referees #2 and 3 and their comments are provided below.

While referee #2 is satisfied with the introduced changes, referee #3 is not convinced that the analysis "as is" provides enough insight for consideration here.

I have discussed the comments further with referee #2. We both appreciate the raised points and see where the referee is coming from. However, we also find that the analysis provides important insight that will be of value to the field and stimulate further research on this topic.

I would therefore like to ask you to submit a revised version by addressing the raised concerns with text changes either in the point-by-point response or in the MS text. Will you also make sure that you have a balanced discussion where you indicate the limitations of the analysis and what the data can tell you and what it can't.

When you submit the revised version will you also take care of the following editorial points:

- Please correct the reference format to EMBO journal style

- We need a Disclosure and competing interests statement

- The movie needs to be renamed to Movie EV1 and called out in the MS text. Please zip the movie with the legend together.

- The source data needs to be reorganized. Main figures: Should be one file/folder per figure zipped. The EV figures should be zipped together and the appendix figures zipped together and then the EV and Appendix files zipped in one file. See also https://www.embopress.org/pb-assets/embo-site/Guide%20for%20SourceData%20Submission-1666879490817.pdf

- We include a synopsis of the paper that is visible on the html file (see http://emboj.embopress.org/). Can you provide me with a general summary statement and 3-5 bullet points that capture the key findings of the paper?

- I also need a summary figure for the synopsis. The size should be 550 wide by [200-400] high (pixels).

- Our publisher has also done their pre-publication check on your manuscript. When you log into the manuscript submission system you will see the file "Data Edited Manuscript file". Take a look at the word file and the comments regarding the figure legends and respond to the issues.

That should be all - let me know if you have any further questions

With best wishes

Karin

Karin Dumstrei, PhD
Senior Editor
The EMBO Journal

Instructions for preparing your revised manuscript:

Guide For Authors: https://www.embopress.org/page/journal/14602075/authorguide

We realize that it is difficult to revise to a specific deadline. In the interest of protecting the conceptual advance provided by the work, we recommend a revision within 3 months (1st Feb 2023).

Use the link below to submit your revision:

https://emboj.msubmit.net/cgi-bin/main.plex

Referee #2:

I very much appreciate the responses of Pettmann et al. to my previous comments. All issues raised have been resolved in the revised version of the ms..

This is a very elegant and important study which I consider a much needed contribution to ongoing discussions in the field. I wholeheartedly recommend publication in EMBO Journal.

Referee #3:

In general, I do not think authors have really addressed my major concerns, especially the technical concerns of "wet expeirments" as well as "wet data" quality, analysis and interprepation. so their data can not well support their conclusion. More importantly, I do not really think its scientific advances of this study is significant enough for EMBO J.

Major concers are still remains:
1. In Fig.2B, data quality and analysis reamin to be concerned to me. How could the survivall rate be larger than 1.0 when force is 4.1pN? compared to original data in the first submission, the distribution looks normal to me at this force. After authors "so-called" corrected their data after revision, I feel more concerns about the data quality.
2. I feel also very much concerned about the fiting in fig.2C-G panels (force vs off-rate). Authors use Bell model to fit their data. As Bell model only has one dissociation pathway, it is well known that this model is unable to discribe the catch bond behavior, unless using two state models like Evans (2004 PNAS) or Zhu group did before for selectin's studies. Moreover, for demonstating the goodness of the fitting, at least you should present these data in a semi log to show the data are linearly fit. I don't think many conditions are not well fitted by bell model. Especially, for Fig.2G, A6/Tax7Q panel, I do not think this data can be fitted by bell model? the xb they got is negative , would it be meaningful? If can't be fitted by bell model, authors should really think of other models instead of Bell's. for example, you can look at "Shiwen Guo et al., Communication Biology, 2019;
3. I still hold my significant concerns about their flow chamber assay to characterize single-molecule dissociation kinetics, although they cited several previous flow chamber assay papers in the reponses. The pulling force generated in the assay to the TCR/pMHC bond is really dependent on flow's shear velocity, beads diameter, and molecular length etc. when force increases (c.f. Fig.S1,S2 and standard fluidic mechanics analysis), they needs to increase shear velocity (Fig.S2) given fixed bead size and buffer types (i.e., contant buffer's viscocity). Inevitablly, they would increased the force loading rate at least 10 times more from low force regime to high force regime, given constant molecular stiffness. In contrast, other single-molecule biomechanical assays, they can well control the loading rate in constant. Several papers (Evans, PNAS 2004;Marshall et al., BJ, 2005; Krishna et al., JBC,2011;) clearly shown that force loading rate could affect ligand dissoicaiton kinetics. I'm not sure how would their velocity change affect the their force-dependent kinetics and their extrapulated zero-force off-rate and xb. This uncertainty might also affect their conclusion that force impairs antigen discrimination of TCR. As I said before, the best way is authors to use single-molecule assay to test their TCR-pMHC system and compare with flow-chamber data by themselves. But they directly negelect my suggestion , I don't know why. At least they should test one pair to TCR-pMHC bond.
Regarding authors' response to my 1st and 3rd comments, I still don't agree their argument. TCR and CD3 are highly complexed together, especially after recently complexed structure are revealed by Huang and Davis group (Nature,2019; Mol Cell 2022; Cell 2022). TCR/CD3s are clearly tightly associated as a machinery, strongly suggesting that TCR recognition would be regulated by associated CD3. Furthermore, authors also realized that Liu et al., 2015 clearly showed reduced catch bond for a cell-free TCR, further suggesting the importance of cd3 complex for TCR catch bond formation and TCR antigen recognition. So, if we really want to reveal how TCR recognize antigen and how mechanical force regulates this recognition, testing complexed TCR/CD3 binding with pMHC is necessary. So only looking at abTCR (purified form) binding with pMHC to answer TCR recognition problem seems less scientifically meaningful.

Other issues:
There are still several issues regarding writing more precisely and correctly. they should be more objectively report what has been published , what is known, what is unknown. For example, in abstract, they wrote" how force impacts the TCR/pMHC off-rate remains unclera.". that is not true. Even they selfs have also cited ref.15 : "It is notable that the magnitude of these catch-bonds is appreciably reduced (15) or abolished (16) when applying force to purified forms of the same TCRs."

Response to Reviewers

Omer Dushek
Professor of Molecular Immunology
Wellcome Trust Senior Fellow

Sir William Dunn School of Pathology
University of Oxford
South Parks Road, Oxford, OX1 3RE
P. +44 1865 275576
E. omer.dushek@path.ox.ac.uk

Wednesday November 9, 2022

Dear Dr. Karin Dumstrei,

We respectfully resubmit our revised manuscript, 'Mechanical forces impair antigen discrimination by reducing differences in T cell receptor off-rates', for your considering in the *EMBO Journal*.

We have added additional limitations to the discussion and have provided a point-by-point response below.

We greatly appreciate that you have taken the time to discuss the comments of reviewer 3 with reviewer 2.

We thank you and the three reviewers for taking the time to read our manuscript and for providing constructive comments that have improved it.

Best wishes,

Professor P. Anton van der Merwe
Doctor Philippe Robert
Professor Omer Dushek

Referee #2:

I very much appreciate the responses of Pettmann et al. to my previous comments. All issues raised have been resolved in the revised version of the ms..

This is a very elegant and important study which I consider a much needed contribution to ongoing discussions in the field. I wholeheartedly recommend publication in EMBO Journal.

We thank the reviewer for taking the time to read our revised manuscript.

Referee #3:

In general, I do not think authors have really addressed my major concerns, especially the technical concerns of "wet expeirments" as well as "wet data" quality, analysis and interprepation. so their data can not well support their conclusion. More importantly, I do not really think its scientific advances of this study is significant enough for EMBO J.

We thank the reviewer for taking the time to read our revised manuscript.

Major concers are still remains:
1. In Fig.2B, data quality and analysis reamin to be concerned to me. How could the survivall rate be larger than 1.0 when force is 4.1pN? compared to original data in the first submission, the distribution looks normal to me at this force. After authors "so-called" corrected their data after revision, I feel more concerns about the data quality.

The corrected survival distribution can be larger than 1 because we are now subtracting the survival distribution of non-specific interactions (this control was requested by Reviewer 2). Under very low forces (4.1 pN), it is possible for the number of non-specific interactions to be similar to the number of specific interactions and as a result, the survival can appear to be larger than 1. As expected, even at these low forces, the survival is quickly below 1 because there are more specific interactions that have longer survival durations. We note that all of the non-corrected data in the original submission is included in the appendix.

2. I feel also very much concerned about the fiting in fig.2C-G panels (force vs off-rate). Authors use Bell model to fit their data. As Bell model only has one dissociation pathway, it is well known that this model is unable to discribe the catch bond behavior, unless using two state models like Evans (2004 PNAS) or Zhu group did before for selectin's studies. Moreover, for demonstating the goodness of the fitting, at least you should present these data in a semi log to show the data are linearly fit. I don't think many conditions are not well fitted by bell model. Especially, for Fig.2G, A6/Tax7Q panel, I do not think this data can be fitted by bell model? the xb they got is negative , would it be meaningful? If can't be fitted by bell model, authors should really think of other models instead of Bell's. for example, you can look at "Shiwen Guo et al., Communication Biology, 2019;

Bell's model is an empirical model that can describe slip (xb > 0) and catch (xb < 0) bonds. This is demonstrated in Figure 1B of our manuscript. In general, the data points appear above and below the fit of Bell's model (i.e. residuals are randomly distributed), which is a

hallmark of a good fit. We agree that Bell's model can only capture data where off-rates increase or decrease as force is increased and as a result, we were unable to include 3 interactions in our analysis (Appendix Figure S13). Importantly, the key conclusion that we are making is that forces disproportionately impact the off-rate of higher-affinity interactions compared to lower-affinity interactions, which is clearly seen in the data even before fitting Bell's model. We first explain this point in the text before discussing the result of Bell's model (compare NYE 6V to 4D in Fig 2D or Tax WT to 5H in Fig 2G for example). We have used Bell's model as a way of summarizing this observation using xb (Fig 2F and 2I). We also note that a control of fitting Bell's model is that the extrapolated zero-force off-rate obtained from fitting Bell's model is correlated to the solution affinity as measured by an independent instrument, namely SPR (Fig 2E,F). Therefore, Bell's model is not necessary to observe our key conclusion and the fit of Bell's model does produce reasonable estimates for the off-rate at zero-force.

3. I still hold my significant concerns about their flow chamber assay to characterize single-molecule dissociation kinetics, although they cited several previous flow chamber assay papers in the reponses. The pulling force generated in the assay to the TCR/pMHC bond is really dependent on flow's shear velocity, beads diameter, and molecular length etc. when force increases (c.f. Fig.S1,S2 and standard fluidic mechanics analysis), they needs to increase shear velocity (Fig.S2) given fixed bead size and buffer types (i.e., contant buffer's viscocity). Inevitablly, they would increased the force loading rate at least 10 times more from low force regime to high force regime, given constant molecular stiffness. In contrast, other single-molecule biomechanical assays, they can well control the loading rate in constant. Several papers (Evans, PNAS 2004;Marshall et al., BJ, 2005; Krishna et al., JBC,2011;) clearly shown that force loading rate could affect ligand dissoicaiton kinetics. I'm not sure how would their velocity change affect the their force-dependent kinetics and their extrapulated zero-force off-rate and xb. This uncertainty might also affect their conclusion that force impairs antigen discrimination of TCR. As I said before, the best way is authors to use single-molecule assay to test their TCR-pMHC system and compare with flow-chamber data by themselves. But they directly negelect my suggestion , I don't know why. At least they should test one pair to TCR-pMHC bond.

In AFM and BFP, loading rates are an inevitable consequence of the use of springs as means of force measurement. In LFC (present study), there is an order of magnitude delay between bond kinetics and any force application because of the stretching (straightening) of the ligand and receptor and their associated linkers (consider that there is a ~32 nm long assembly (see Fig EV2 for a schematic) and velocities ranging from 10 to 100 μm/sec, the stretching/straightening duration ranges from 0.3 to 3 ms). Thus, the force is applied <u>only</u> when the assembly is fully stretched/straightened: the loading rate itself is dependent only on the spring constant of the assembly. It follows that force is applied instantly on a bond of which spring constant is an intrinsic parameter (if the antibody linker, invariant in our study and structurally very close to TCR and MHC molecules, is put aside). Taken together, our experimental system does not have the complication of a progressively applied force when we assess bond behaviour at a given force.

We note that the BFP and AFM force ramps durations are in the order of magnitude of one or several hundreds of ms; contact times between both surfaces before pulling are in the

same range in these methods. It is therefore difficult to assert with certainty what effect on bond lifetime is the consequence of loading rate itself or of other possibilities occurring during force ramp: first, bonds may mature during these hundred of millisecond long durations; second, bond lifetimes are in similar order of magnitude and early breaking of a fraction of them in a time-dependent manner may occur. In LFC, the intrinsic loading is restricted to very short durations with fewer potential artefacts. Lastly, we have used the OT-I TCR, which has heavily been used by the BFP community and have produced the observed catch bond.

4. Regarding authors' response to my 1st and 3rd comments, I still don't agree their argument. TCR and CD3 are highly complexed together, especially after recently complexed structure are revealed by Huang and Davis group (Nature,2019; Mol Cell 2022; Cell 2022). TCR/CD3s are clearly tightly associated as a machinery, strongly suggesting that TCR recognition would be regulated by associated CD3. Furthermore, authors also realized that Liu et al., 2015 clearly showed reduced catch bond for a cell-free TCR, further suggesting the importance of cd3 complex for TCR catch bond formation and TCR antigen recognition. So, if we really want to reveal how TCR recognize antigen and how mechanical force regulates this recognition, testing complexed TCR/CD3 binding with pMHC is necessary. So only looking at abTCR (purified form) binding with pMHC to answer TCR recognition problem seems less scientifically meaningful.

We note that the full TCR-CD3 complex is not necessary to observe a catch bond. First, in our study we have found a catch bond for A6 binding 7Q and OT-I binding OVA using purified TCRa/b without CD3. In the case of A6, we note that the TCR itself was unchanged but rather the ligand was varied, which suggests that catch bonds can be observed as a result of differences in the contact interface between TCR and pMHC (without any impact of CD3). This has also recently been suggested by Chris Garcia (Zhao et al (2022) Science) who used the BFP assay on live T cells to show that changes in the CDR2 loops can change a TCR from exhibiting a slip bond to a catch bond.

The differences between LFC using purified proteins (present study) and the BFP using live T cells are:
1) Use of the full TCR-CD3 complex in BFP (as pointed out by the reviewer), and
2) The repeated use of live T cells in BFP, which means that TCR clustering, cytoskeleton rearrangements, signalling feedbacks, etc can also explain differences between our assays.

We have revised the discussion to include a paragraph on limitations of our assay and included the reviewers point on CD3 and have suggested that future BFP experiments are conducted using the full TCR-CD3 complex on membranes rather than intact live T cells that allow signalling.

Other issues:
There are still several issues regarding writing more precisely and correctly. they should be more objectively report what has been published , what is known, what is unknown. For example, in abstract, they wrote" how force impacts the TCR/pMHC off-rate remains unclera.". that is not true. Even they selfs have also cited ref.15 : "It is notable that the

magnitude of these catch-bonds is appreciably reduced (15) or abolished (16) when applying force to purified forms of the same TCRs."

Although the reviewer notes ‘several issues’, only a single one is explained and we have addressed it by changing “unclear” to “debated” in the abstract.

2nd Revision - Editorial Decision 16th Nov 2022

Dear Omer,

Thanks for submitting your revised manuscript to The EMBO Journal. I have now looked at everything and all looks good.

I am therefore very pleased to accept the manuscript for publication here.

With best wishes

Karin

Karin Dumstrei, PhD
Senior Editor
The EMBO Journal

Please note that it is EMBO Journal policy for the transcript of the editorial process (containing referee reports and your response letter) to be published as an online supplement to each paper. If you do NOT want this, you will need to inform the Editorial Office via email immediately. More information is available here: https://www.embopress.org/page/journal/14602075/authorguide#transparentprocess

Your manuscript will be processed for publication in the journal by EMBO Press. Manuscripts in the PDF and electronic editions of The EMBO Journal will be copy edited, and you will be provided with page proofs prior to publication. Please note that supplementary information is not included in the proofs.

You will be contacted by Wiley Author Services to complete licensing and payment information. The required 'Page Charges Authorization Form' is available here: https://www.embopress.org/pb-assets/embo-site/tej_apc.pdf - please download and complete the form and return to embopressproduction@wiley.com

EMBO Press participates in many Publish and Read agreements that allow authors to publish Open Access with reduced/no publication charges. Check your eligibility: https://authorservices.wiley.com/author-resources/Journal-Authors/open-access/affiliation-policies-payments/index.html

Should you be planning a Press Release on your article, please get in contact with embojournal@wiley.com as early as possible, in order to coordinate publication and release dates.

If you have any questions, please do not hesitate to call or email the Editorial Office. Thank you for your contribution to The EMBO Journal.

** Click here to be directed to your login page: https://emboj.msubmit.net

EMBO Press Author Checklist

Corresponding Author Name: Omer Dushek	
Journal Submitted to: EMBO Journal	
Manuscript Number: EMBOJ-2022-111841	

USEFUL LINKS FOR COMPLETING THIS FORM
The EMBO Journal - Author Guidelines
EMBO Reports - Author Guidelines
Molecular Systems Biology - Author Guidelines
EMBO Molecular Medicine - Author Guidelines

Reporting Checklist for Life Science Articles (updated January
This checklist is adapted from Materials Design Analysis Reporting (MDAR) Checklist for Authors. MDAR establishes a minimum set of requirements in transparent reporting in the life sciences (see Statement of Task: 10.31222/osf.io/9sm4x). Please follow the journal's guidelines in preparing your
Please note that a copy of this checklist will be published alongside your article.

Abridged guidelines for figures
1. Data
The data shown in figures should satisfy the following conditions:
- the data were obtained and processed according to the field's best practice and are presented to reflect the results of the experiments in an accurate and unbiased manner.
- ideally, figure panels should include only measurements that are directly comparable to each other and obtained with the same assay.
- plots include clearly labeled error bars for independent experiments and sample sizes. Unless justified, error bars should not be shown for technical
- if n<5, the individual data points from each experiment should be plotted. Any statistical test employed should be justified.
- Source Data should be included to report the data underlying figures according to the guidelines set out in the authorship guidelines on Data

2. Captions
Each figure caption should contain the following information, for each panel where they are relevant:
- a specification of the experimental system investigated (eg cell line, species name).
- the assay(s) and method(s) used to carry out the reported observations and measurements.
- an explicit mention of the biological and chemical entity(ies) that are being measured.
- an explicit mention of the biological and chemical entity(ies) that are altered/varied/perturbed in a controlled manner.
- the exact sample size (n) for each experimental group/condition, given as a number, not a range;
- a description of the sample collection allowing the reader to understand whether the samples represent technical or biological replicates (including how many animals, litters, cultures, etc.).
- a statement of how many times the experiment shown was independently replicated in the laboratory.
- definitions of statistical methods and measures:
  - common tests, such as t-test (please specify whether paired vs. unpaired), simple χ2 tests, Wilcoxon and Mann-Whitney tests, can be unambiguously identified by name only, but more complex techniques should be described in the methods section;
  - are tests one-sided or two-sided?
  - are there adjustments for multiple comparisons?
  - exact statistical test results, e.g., P values = x but not P values < x;
  - definition of 'center values' as median or average;
  - definition of error bars as s.d. or s.e.m.

Please complete ALL of the questions below.
Select "Not Applicable" only when the requested information is not relevant for your study.

Materials

Newly Created Materials	Information included in the manuscript?	In which section is the information available? (Reagents and Tools Table, Materials and Methods, Figures, Data Availability Section)
New materials and reagents need to be available; do any restrictions apply?	Not Applicable	

Antibodies	Information included in the manuscript?	In which section is the information available? (Reagents and Tools Table, Materials and Methods, Figures, Data Availability Section)
For **antibodies** provide the following information:<br>- Commercial antibodies: RRID (if possible) or supplier name, catalogue number and or/clone number<br>- Non-commercial: RRID or citation	Yes	Methods (Laminar flow assay, 1 antibody is used in the study)

DNA and RNA sequences	Information included in the manuscript?	In which section is the information available? (Reagents and Tools Table, Materials and Methods, Figures, Data Availability Section)
**Short novel DNA or RNA including primers, probes**: provide the sequences.	Not Applicable	

Cell materials	Information included in the manuscript?	In which section is the information available? (Reagents and Tools Table, Materials and Methods, Figures, Data Availability Section)
**Cell lines:** Provide species information, strain. Provide accession number in repository **OR** supplier name, catalog number, clone number, and/**OR** RRID.	Not Applicable	
**Primary cultures:** Provide species, strain, sex of origin, genetic modification status.	Not Applicable	
Report if the cell lines were recently **authenticated** (e.g., by STR profiling) and tested for mycoplasma contamination.	Not Applicable	

Experimental animals	Information included in the manuscript?	In which section is the information available? (Reagents and Tools Table, Materials and Methods, Figures, Data Availability Section)
**Laboratory animals or Model organisms:** Provide species, strain, sex, age, genetic modification status. Provide accession number in repository **OR** supplier name, catalog number, clone number, **OR** RRID.	Not Applicable	
**Animal observed in or captured from the field:** Provide species, sex, and age where possible.	Not Applicable	
Please detail **housing and husbandry conditions**.	Not Applicable	

Plants and microbes	Information included in the manuscript?	In which section is the information available? (Reagents and Tools Table, Materials and Methods, Figures, Data Availability Section)
**Plants:** provide species and strain, ecotype and cultivar where relevant, unique accession number if available, and source (including location for collected wild specimens).	Not Applicable	
**Microbes:** provide species and strain, unique accession number if available, and source.	Yes	Methods (protein expression and purification)

Human research participants	Information included in the manuscript?	In which section is the information available? (Reagents and Tools Table, Materials and Methods, Figures, Data Availability Section)
If collected and within the bounds of privacy constraints report on age, sex and gender or ethnicity for all study participants.	Not Applicable	

Core facilities	Information included in the manuscript?	In which section is the information available? (Reagents and Tools Table, Materials and Methods, Figures, Data Availability Section)
If your work benefited from core facilities, was their service mentioned in the acknowledgments section?	Not Applicable	

Design

Study protocol	Information included in the manuscript?	In which section is the information available? (Reagents and Tools Table, Materials and Methods, Figures, Data Availability Section)
If study protocol has been **pre-registered, provide DOI in the manuscript**. For clinical trials, provide the trial registration number **OR** cite DOI.	Not Applicable	
Report the **clinical trial registration number** (at ClinicalTrials.gov or equivalent), where applicable.	Not Applicable	

Laboratory protocol	Information included in the manuscript?	In which section is the information available? (Reagents and Tools Table, Materials and Methods, Figures, Data Availability Section)
Provide DOI OR other citation details if **external detailed step-by-step protocols** are available.	Not Applicable	

Experimental study design and statistics	Information included in the manuscript?	In which section is the information available? (Reagents and Tools Table, Materials and Methods, Figures, Data Availability Section)
Include a statement about **sample size** estimate even if no statistical methods were used.	Not Applicable	
Were any steps taken to minimize the effects of subjective bias when allocating animals/samples to treatment (e.g. **randomization procedure**)? If yes, have they been described?	Not Applicable	
Include a statement about **blinding** even if no blinding was done.	Not Applicable	
Describe **inclusion/exclusion criteria** if samples or animals were excluded from the analysis. Were the criteria pre-established?<br><br>If sample or data points were omitted from analysis, report if this was due to attrition or intentional exclusion and provide justification.	Yes	Methods (Data Analysis)
For every figure, are **statistical tests** justified as appropriate? Do the data meet the assumptions of the tests (e.g., normal distribution)? Describe any methods used to assess it. Is there an estimate of variation within each group of data? Is the variance similar between the groups that are being statistically compared?	Yes	Figure (Fig 4G,H)

Sample definition and in-laboratory replication	Information included in the manuscript?	In which section is the information available? (Reagents and Tools Table, Materials and Methods, Figures, Data Availability Section)
In the figure legends: state number of times the experiment was **replicated** in laboratory.	Yes	Figure (Fig 2,3)
In the figure legends: define whether data describe **technical or biological replicates**.	Not Applicable	

Ethics

Ethics	Information included in the manuscript?	In which section is the information available? (Reagents and Tools Table, Materials and Methods, Figures, Data Availability Section)
Studies involving **human participants**: State details of **authority granting ethics approval** (IRB or equivalent committee(s), provide reference number for approval.	Not Applicable	
Studies involving **human participants**: Include a statement confirming that **informed consent** was obtained from all subjects and that the experiments conformed to the principles set out in the WMA Declaration of Helsinki and the Department of Health and Human Services Belmont Report.	Not Applicable	
Studies involving **human participants:** For publication of **patient photos**, include a statement confirming that consent to publish was obtained.	Not Applicable	
Studies involving experimental **animals**: State details of **authority granting ethics approva**l (IRB or equivalent committee(s), provide reference number for approval. Include a statement of compliance with ethical regulations.	Not Applicable	
Studies involving **specimen and field samples:** State if relevant **permits** obtained, provide details of authority approving study; if none were required, explain why.	Not Applicable	

Dual Use Research of Concern (DURC)	Information included in the manuscript?	In which section is the information available? (Reagents and Tools Table, Materials and Methods, Figures, Data Availability Section)
Could your study fall under dual use research restrictions? Please check biosecurity documents and list of **select agents and toxins** (CDC): https://www.selectagents.gov/sat/list.htm	Not Applicable	
If you used a select agent, is the security level of the lab appropriate and reported in the manuscript?	Not Applicable	
If a study is subject to dual use research of concern regulations, is the name of the **authority granting approval and reference number** for the regulatory approval provided in the manuscript?	Not Applicable	

Reporting

The MDAR framework recommends adoption of discipline-specific guidelines, established and endorsed through community initiatives. Journals have their own policy about requiring specific guidelines and recommendations to complement MDAR.

Adherence to community standards	Information included in the manuscript?	In which section is the information available? (Reagents and Tools Table, Materials and Methods, Figures, Data Availability Section)
State if relevant guidelines or checklists (e.g., **ICMJE, MIBBI, ARRIVE, PRISMA**) have been followed or provided.	Not Applicable	
For **tumor marker prognostic studies,** we recommend that you follow the **REMARK** reporting guidelines (see link list at top right). See author guidelines, under 'Reporting Guidelines'. Please confirm you have followed these guidelines.	Not Applicable	
For **phase II and III randomized controlled trials**, please refer to the **CONSORT** flow diagram (see link list at top right) and submit the CONSORT checklist (see link list at top right) with your submission. See author guidelines, under 'Reporting Guidelines'. Please confirm you have submitted this list.	Not Applicable	

Data Availability

Data availability	Information included in the manuscript?	In which section is the information available? (Reagents and Tools Table, Materials and Methods, Figures, Data Availability Section)
Have **primary datasets** been deposited according to the journal's guidelines (see 'Data Deposition' section) and the respective accession numbers provided in the Data Availability Section?	Not Applicable	
Were **human clinical and genomic datasets** deposited in a public access-controlled repository in accordance to ethical obligations to the patients and to the applicable consent agreement?	Not Applicable	
Are **computational models** that are central and integral to a study available without restrictions in a machine-readable form? Were the relevant accession numbers or links provided?	Not Applicable	
If publicly available data were reused, provide the respective **data citations in the reference list**.	Yes	Figure (Fig 4F)
